# Synthesis, Characterization, Biological Evaluation and In Silico Studies of Some New Thiazole Derivatives

**DOI:** 10.3390/ph19091468

**Published:** 2026-09-16

**Authors:** Tuğçe Çaklılı, Yusuf Sıcak, Somdatta Y. Chaudhari, Suraj N. Mali, Dilaycan Çam, Gülin Gümüşbulut Şener, Mehmet Öztürk, Mert Ülgen, Fatih Tok

**Affiliations:** 1 Department of Pharmaceutical Chemistry, Faculty of Pharmacy, Acıbadem Mehmet Ali Aydınlar University, İstanbul 34752, Türkiye; tugce.caklili@acibadem.edu.tr (T.Ç.); mert.ulgen@acibadem.edu.tr (M.Ü.); 2 Institute of Health Sciences, Marmara University, İstanbul 34865, Türkiye; 3 Department of Medicinal and Aromatic Plants, Köyceğiz Vocational School, Mugla Sitki Kocman University, Muğla 48800, Türkiye; ysicak@gmail.com; 4 Department of Pharmaceutical Chemistry, PES’s Modern College of Pharmacy, Pune 411044, Maharashtra, India; somu.chaudhari@gmail.com; 5 School of Pharmacy, D. Y. Patil University, Navi Mumbai 400706, Maharashtra, India; suraj.mali@dypatil.edu; 6 Department of Chemistry, Faculty of Sciences, Mugla Sitki Kocman University, Muğla 48000, Türkiye; dilaycancam@gmail.com (D.Ç.); gulingumusbulut@posta.mu.edu.tr (G.G.Ş.); mehmetozturk@mu.edu.tr (M.Ö.); 7 Faculty of Chemistry and Chemical Technology, Farabi University, Almaty 050040, Kazakhstan; 8 Department of Pharmaceutical Chemistry, Faculty of Pharmacy, Marmara University, İstanbul 34854, Türkiye

**Keywords:** thiazole, benzodioxole, antioxidant activity, anticholinesterase activity, α-amylase inhibition activity, α-glycosidase inhibition activity, molecular docking

## Abstract

**Background/Objectives**: The thiazole ring is one of the core structures frequently favored in the design of new active compounds. **Methods**: A series of thiazole derivatives bearing a benzodioxole scaffold (1-9) were synthesized and evaluated for their antioxidant, anticholinesterase, and antidiabetic activities. **Results**: Antioxidant assays (ABTS and DPPH) revealed that compounds bearing 4-trifluoromethyl (compound **8**) and phenyl (compound **9**) moieties exhibited the strongest radical scavenging activities, attributed to efficient hydrogen atom transfer and extended π-conjugation, respectively. Enzyme inhibition studies showed that electron-withdrawing substituents such as 4-SO_2_CH_3_ (compound **3**) and 3,4-diCl (compound **5**) enhanced both AChE/BChE and α-amylase/α-glucosidase inhibition, surpassing the activity of acarbose in antidiabetic assays, while electron-donating groups reduced potency. Molecular docking and MD studies have shed light on the interactions of the active compounds. In addition, in silico ADMET predictions for the compounds have been performed. **Conclusions**: These results indicate that the thiazole-benzodioxole scaffold is a promising framework for developing multifunctional agents with combined neuroprotective, antidiabetic, and antioxidant activities.

## 1. Introduction

Alzheimer’s disease (AD) and Type 2 Diabetes Mellitus (T2DM) are two major pathologies whose incidence is rapidly increasing worldwide [1,2]. Recent epidemiological and pathophysiological studies have revealed a strong biological relationship between T2DM and AD due to shared cellular abnormalities such as insulin resistance [3], accumulation of advanced glycation end products (AGEs) [4], and severe oxidative stress [5]. AD is frequently referred to as “Type 3 Diabetes” [6]. In the pathogenesis of AD, the disruption of cholinergic neurotransmission in the cerebral cortex and hippocampus is one of the primary reasons for cognitive decline. Therefore, the inhibition of acetylcholinesterase (AChE) and butyrylcholinesterase (BChE) enzymes, which catalyze the hydrolysis of acetylcholine, remains the most widely accepted cornerstone therapeutic approach in the symptomatic treatment of the disease [7]. On the other hand, the primary aim in the clinical management of T2DM is to slow down the hydrolysis of dietary carbohydrates by inhibiting the α-amylase and α-glucosidase enzymes in the gastrointestinal tract, thereby controlling postprandial hyperglycemia. Furthermore, both T2DM and AD share fundamental underlying pathomechanisms, notably the overproduction of reactive oxygen species (ROS) and chronic oxidative stress, which severely exacerbate neurodegeneration and insulin resistance [8].

Given the complex, multifactorial nature of these intertwined pathologies, the traditional “one-target, one-drug” pharmacological paradigm often falls short of providing comprehensive therapeutic efficacy [9]. Consequently, the multi-target-directed ligand (MTDL) has become a key component of modern medicinal chemistry as a rational approach to overcome these limitations [10]. By covalently integrating distinct pharmacophoric moieties into a single molecular scaffold, MTDLs can simultaneously modulate multiple biological targets—such as cholinesterases, carbohydrate-hydrolyzing enzymes, and free radicals—thereby offering a synergistic therapeutic effect while minimizing the risk of drug–drug interactions and adverse side effects commonly associated with polypharmacy regimens. In the rational design of novel MTDLs, heterocyclic systems are extensively utilized due to their favorable pharmacokinetic profiles and versatile receptor-binding capabilities [11]. The 1,3-benzodioxole scaffold is a privileged structure in drug discovery, recognized for its excellent lipophilicity, which facilitates blood-brain barrier penetration [12]. Recent biological evaluations have highlighted the profound antidiabetic potential of benzodioxole derivatives, demonstrating exceptional α-amylase and α-glucosidase inhibitory activities by forming critical hydrogen bonds and hydrophobic interactions with key amino acid residues in the enzyme active sites, occasionally achieving sub-micromolar IC_50_ values [13]. Similarly, the thiazole ring is a highly versatile pharmacophore known to establish robust π–π and cation–π interactions with both the catalytic active site (CAS) and the peripheral anionic site (PAS) of cholinesterases, while also exhibiting potent inhibitory effects against α-glucosidase [14]. For this reason, the high biological potential that can be achieved by combining 1,3-benzodioxole and thiazole rings in the design of new MTDLs is of critical importance. Linking these two distinct pharmacophores (the 1,3-benzodioxole and thiazole rings) via a hydrazone (azomethine) bridge provides the conformational flexibility necessary for optimal spatial fit within various enzyme pockets. Furthermore, thanks to its potential electron donor-acceptor and transition metal chelating properties, it also significantly enhances the molecule’s overall anticholinesterase and antioxidant capacity [15].

Motivated by these structural considerations and the continuous drive to discover potent polypharmacological agents, the design, synthesis, and biological evaluation of a novel series of 1,3-benzodioxole-hydrazone-thiazole hybrids are reported herein. The target compounds were synthesized utilizing 3,4-methylenedioxyacetophenone as the starting material, yielding an ethylidenehydrazinyl linker that connects the benzodioxole moiety to a diverse array of 4-arylthiazole rings. The terminal thiazole ring was constructed using various substituted phenacyl bromides. This synthetic strategy allowed for systematic modifications of the substituents on the phenyl ring, enabling a comprehensive structure-activity relationship (SAR) investigation. The newly synthesized derivatives were evaluated in vitro for their anticholinesterase (AChE and BChE), antidiabetic (α-amylase and α-glucosidase), and antioxidant activities. This study aims to provide valuable structural insights for the development of multi-target drugs for the treatment of Alzheimer’s disease (AD) and type 2 diabetes (T2DM).

## 2. Results and Discussion

### 2.1. Chemistry

**Scheme 1:**
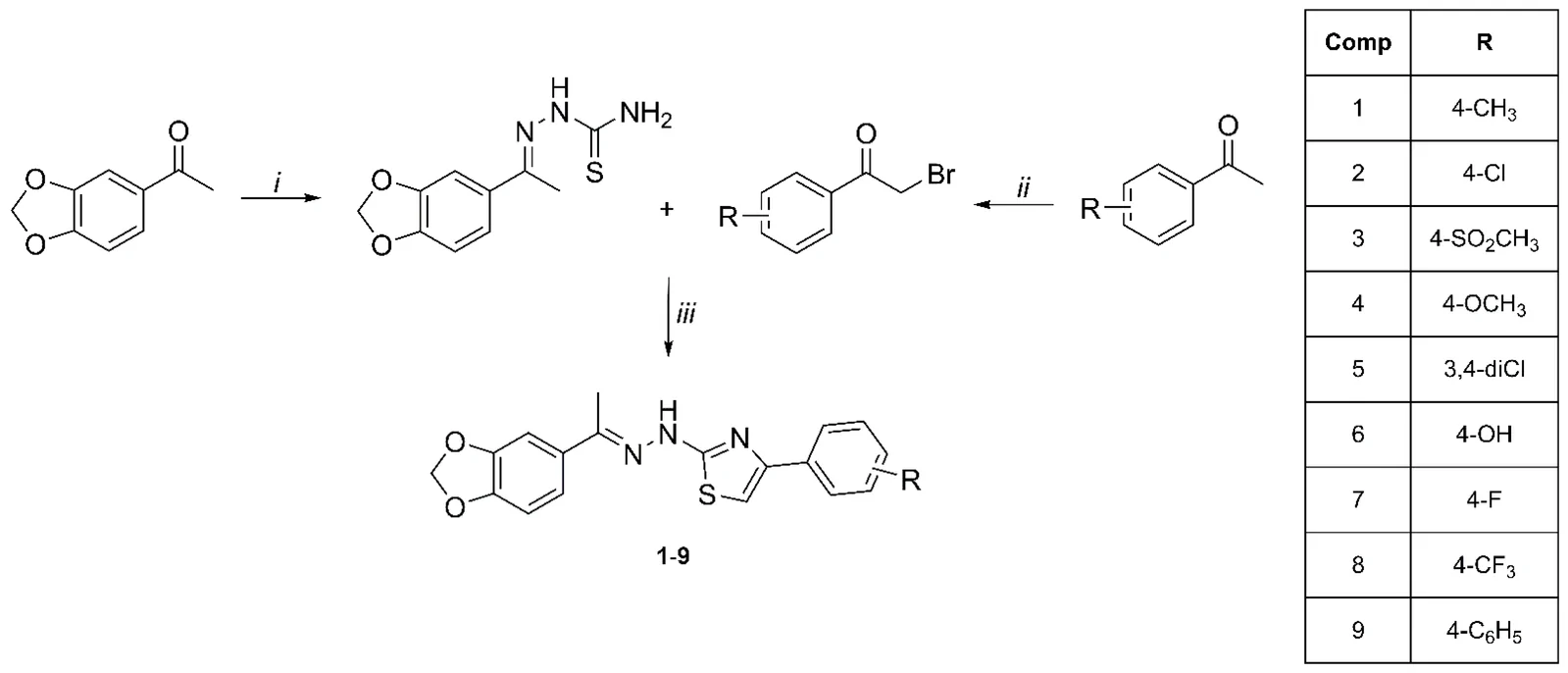
The synthesis pathway of thiazole compounds (**1**-**9**). Reagents and conditions: (*i*) Thiosemicarbazide, ethanol, 95 °C, reflux, 10 h; (*ii*) ether, AlCl_3_, Br_2_, 0 °C, stir; (*iii*) ethanol, CH_3_COOH, 95 °C, reflux, 10 h.

In this study, thiazole rings were synthesized using the Hantzsch thiazole synthesis. According to this method, thioamide derivatives and α-haloketone derivatives are reacted in an organic solvent under acid catalysis [16]. As the thioamide derivative, 2-(1-(benzo[*d*][1,3]dioxol-5-yl)ethylidene)hydrazine-1-carbothioamide was obtained by the condensation reaction of 1-(benzo[*d*][1,3]dioxol-5-yl)ethan-1-one and thiosemicarbazide, the first stage of which is a nucleophilic addition reaction. The α-haloketone derivatives, phenacyl bromides, were obtained by the bromination of acetophenones. The final thiazole compounds (**1**-**9**) were synthesized via nucleophilic attack, cyclization, and dehydration reactions of 2-(1-(benzo[*d*][1,3]dioxol-5-yl)ethylidene)hydrazine-1-carbothioamide with phenacyl bromides, respectively (Scheme 1). The target thiazole compounds (**1**-**9**) were obtained in high yields ranging from 76% to 95%. HPLC was used to determine compound purity, while IR, ^1^H-NMR, ^13^C-NMR, and MS data were used to elucidate their structures. All IR, ^1^H-NMR, ^13^C-NMR, and MS spectra were given in Supplementary Materials as Figures S1–S45.

In the IR spectra of the thiazole compounds (**1**-**9**), the N-H and C=N absorption bands were observed in the 3213–3323 cm^−1^ and 1606–1622 cm^−1^ ranges, respectively. The characteristic O-CH_2_-O and C-S-C absorption bands of the benzo[*d*][1,3]dioxole and thiazole rings were detected in the 1028–1259 cm^−1^ and 744–773 cm^−1^ ranges, respectively.

Previous studies have reported that the hydrazone group can give multiple peaks in NMR spectra due to its *E* and *Z* isomers [17,18]. Therefore, in the ^1^H-NMR spectra, the NH proton of the hydrazone resonated as two separate singlets in the 11.53–12.09 ppm range. The NH protons of compounds **4** and **9**, however, were not observed in the spectrum because they were replaced by deuterium from the solvent. The methylene protons of the [1,3]dioxole ring were detected as a multiplet in the 6.01–6.28 ppm range.

The HSQC spectrum of compound **3**, one of the most active compounds, was recorded and analyzed (Figure S46). The methyl substituent with a methylsulfonyl structure, as the R group, produced contours at 3.27 ppm and 44.0 ppm in the HSQC spectrum. The methyl substituent attached to the azomethine group produced another contour at 2.27–2.56 ppm and 14.7 ppm. The methylenedioxy group was attributed to the contour at 6.07 ppm and 101.9 ppm. Due to the *E* and *Z* isomers of the compounds, the peaks that should have been observed as singlets were split.

In the ^13^C-NMR spectra of the compounds, the carbon atoms at the 2nd and 5th positions of the thiazole ring were identified in the ranges of 163.7–169.4 ppm and 105.9–107.2 ppm, respectively. The methylene carbon of the [1,3]dioxole ring was recorded in the 101.8–102.4 ppm range. The imine (CH=N) carbon of the hydrazone group was obtained in the 148.1–148.8 ppm range.

### 2.2. Biological Activity

Antioxidant activity results of thiazole compounds were given in Table 1. According to the ABTS assay results, the activity ranking of the synthesized compounds was approximately **9** > **8** > **6** > **7** > **5** > **4** > **2** > **3** > **1**, while BHA showed the strongest activity as the reference antioxidant. Among the series, compound **9** bearing a 4-phenyl substituent exhibited the highest ABTS radical scavenging activity (IC_50_ = 9.28 µM), which may be attributed to the extended π-conjugation provided by the additional phenyl ring, facilitating stabilization of radical intermediates. Similarly, compound **8** containing a 4-CF_3_ group (IC_50_ = 10.17 µM) and compound **6** with a 4-OH substituent (IC_50_ = 11.65 µM) demonstrated very strong activity. The high activity of compound **6** may be explained by the efficient hydrogen atom transfer (HAT) ability of the phenolic OH group, enabling effective neutralization of the ABTS radical. Compound **7** bearing a 4-F substituent also displayed strong activity (IC_50_ = 13.90 µM), possibly due to the strong inductive electron-withdrawing effect and small atomic size of fluorine, which may contribute to stabilization of the radical species. Compound **5** with a 3,4-dichloro substitution showed notable activity (IC_50_ = 25.17 µM), suggesting that the cumulative electron-withdrawing effect and increased lipophilicity of two chlorine atoms may enhance radical interaction. Compounds containing electron-donating substituents such as 4-OCH_3_ (compound **4**, IC_50_ = 44.15 µM) exhibited moderate activity, likely due to the resonance electron-donating effect of the methoxy group increasing electron density in the aromatic system. Compound **2** (4-Cl, IC_50_ = 45.95 µM) also showed moderate activity, whereas compounds **3** (4-SO_2_CH_3_, IC_50_ = 64.18 µM) and **1** (4-CH_3_, IC_50_ = 78.12 µM) displayed comparatively lower activity, which may be related to the limited contribution of the methyl group to radical stabilization and the strong but resonance-limited electron-withdrawing nature of the sulfonyl group.

The DPPH radical scavenging assay revealed an activity order of **9** > **8** > **6** > **7** > **5** > **4** > **3** > **1** > **2**, with BHA again serving as the reference antioxidant. Compound **9** exhibited the strongest activity in the series (IC_50_ = 2.05 µM), even surpassing the reference antioxidant BHA, which can be attributed to the extended π-conjugation provided by the phenyl substituent that enhances radical stabilization. Compound **8** (4-CF_3_) also demonstrated very strong activity (IC_50_ = 3.11 µM), suggesting that the strong electron-withdrawing character of the CF_3_ group may contribute to stabilization of radical intermediates. Compound **6** containing a phenolic OH group showed remarkable activity (IC_50_ = 5.01 µM), likely due to the efficient hydrogen-donating ability of the phenolic moiety, which facilitates radical quenching. Compounds **7** (4-F, IC_50_ = 6.72 µM) and **5** (3,4-diCl, IC_50_ = 10.09 µM) also exhibited strong activity, possibly related to the inductive electron-withdrawing effects of halogen substituents. Compounds bearing electron-donating substituents such as 4-OCH_3_ (compound **4**, IC_50_ = 23.93 µM) and the strongly electron-withdrawing 4-SO_2_CH_3_ group (compound **3**, IC_50_ = 27.27 µM) displayed moderate activity. In contrast, compound **1** (4-CH_3_, IC_50_ = 40.23 µM) and particularly compound **2** (4-Cl, IC_50_ = 45.95 µM) showed comparatively weaker DPPH radical scavenging activity within the series.

In the CUPRAC assay, where a lower A_0.5_ value indicates stronger reducing capacity, the activity ranking was determined as **4** > **2** ≈ **7** ≈ **6** > **8** > **1** > **9** > **5** > **3**, while BHA again showed the strongest reducing power as the reference compound. Among the tested derivatives, compound **4** bearing a 4-OCH_3_ substituent exhibited the most potent reducing capacity in the series (A_0.5_ = 27.82 µM). This enhanced activity may be attributed to the electron-donating resonance (+M) effect of the methoxy group, which increases electron density in the aromatic system and facilitates electron transfer to reduce Cu(II) ions. Compounds **2** (4-Cl, A_0.5_ = 50.50 µM), **7** (4-F, A_0.5_ = 51.31 µM), and **6** (4-OH, A_0.5_ = 54.89 µM) displayed moderate reducing capacities. Compound **8** containing a 4-CF_3_ substituent showed relatively weak to moderate activity (A_0.5_ = 69.25 µM), while compound **1** (4-CH_3_, A_0.5_ = 71.46 µM) and compound **9** (4-phenyl, A_0.5_ = 78.62 µM) exhibited weaker reducing abilities. Compounds **5** (3,4-diCl, A_0.5_ = 86.18 µM) and especially compound **3** (4-SO_2_CH_3_, A_0.5_ = 94.53 µM) showed the lowest CUPRAC activity in the series, which may be associated with the strong electron-withdrawing character of these substituents, reducing the overall electron-donating capacity of the molecules.

The results obtained for the thiazole derivatives (**1**-**9**) indicate that antioxidant activity is largely related to the electronic properties of the substituents on the aromatic ring, their hydrogen-donating ability, and the degree of π-conjugation. In particular, compound **6** bearing a 4-OH substituent exhibited very strong activity in both the ABTS and DPPH assays; the high activity can be explained by the facile transfer of the phenolic hydrogen to radical species and the resonance stabilization of the resulting phenoxyl radical. Similarly, compound **9** with a 4-phenyl substituent extended the conjugation within the aromatic system, enhancing the stabilization of radical intermediates, and therefore became one of the most active derivatives in both radical scavenging assays.

Derivatives containing electron-withdrawing halogen and CF_3_ groups (especially compounds **7** and **8**) also displayed notable radical scavenging activity; this effect may be associated with the ability of these groups to stabilize the generated radical intermediates through their inductive effects. In addition, the high activity of compound **5** bearing a 3,4-dichloro substituent, particularly in the DPPH assay, suggests that the cumulative electronic effects of multiple halogen substituents and the increased lipophilicity may facilitate radical interactions. In contrast, compound **4** bearing a 4-OCH_3_ substituent exhibited the strongest reducing capacity in the CUPRAC assay within the series; the electron-donating resonance effect of the methoxy group may enhance the electron-transfer ability of the molecule, thereby facilitating the reduction of Cu(II) ions.

On the other hand, derivatives having 4-CH_3_ and 4-SO_2_CH_3_ substituents (compounds **1** and **3**) generally showed lower antioxidant activity. This may be attributed to the limited electron-donating effect of the methyl group and the strong but resonance-limited electron-withdrawing character of the sulfonyl group, which may not sufficiently support radical stabilization. Overall, these findings suggest that high antioxidant activity in this series is mainly associated with the presence of a phenolic hydrogen, extended π-conjugation, and substituents possessing favorable electronic properties.

Enzyme inhibition activity results of thiazole compounds were given in Table 2. The synthesized series of the thiazole derivatives (**1**-**9**) demonstrated varying inhibitory activities against AChE and BChE. Among the compounds, **3** (R = 4-SO_2_CH_3_) and **5** (R = 3,4-diCl) exhibited the most potent AChE inhibition with IC_50_ values of 8.7 ± 1.2 µM and 7.5 ± 1.1 µM, respectively, indicating that electron-withdrawing substituents on the aromatic ring enhance binding affinity to the enzyme active site. In contrast, compounds **4** (R = 4-OCH_3_) and **6** (R = 4-OH) showed relatively weaker AChE inhibition (14.0 ± 2.0 µM and 15.2 ± 2.3 µM), suggesting that electron-donating groups may reduce interaction strength. For BChE, a similar trend was observed, with compound **5** displaying the strongest inhibition (28.0 ± 3.6 µM) and compounds bearing hydroxyl or methoxy groups showing weaker activity. These results highlight the influence of electronic and steric properties of the R substituents on cholinesterase inhibition. It should be noted that this trend is based on a limited number of representative examples within a relatively small series and, in the absence of a quantitative structure-activity relationship analysis, should be regarded as a preliminary SAR observation rather than a definitive conclusion. Compounds bearing intermediate electronic character (e.g., 4-F, 4-Cl) did not follow a strictly monotonic trend, suggesting that additional steric and lipophilic factors may also contribute to the observed inhibitory potency. Notably, all synthesized compounds were less potent than the reference drug galantamine (IC_50_ = 1.82 ± 0.30 µM for AChE; IC_50_ = 4.62 ± 0.12 µM for BChE), yet they offer a scaffold for further optimization.

A more quantitative comparison with the reference drugs further underscores the potential of compounds **3** and **5**. For AChE, compounds **3** and **5** were approximately 4.8-fold and 4.1-fold less potent than galantamine (IC_50_ = 1.82 µM), respectively, while for BChE they were 6.7-fold and 6.1-fold less potent than galantamine (IC_50_ = 4.62 µM). In contrast, for the carbohydrate-hydrolyzing enzymes, compounds **3** and **5** markedly outperformed acarbose, being approximately 2.6-fold and 3.0-fold more potent against α-amylase (IC_50_ = 62.92 µM), and 14.7-fold and 16.0-fold more potent against α-glucosidase (IC_50_ = 271.92 µM), respectively. These quantitative comparisons highlight that, although compounds **3** and **5** do not surpass galantamine in cholinesterase inhibition, their antidiabetic potency substantially exceeds that of the clinically used reference drug acarbose, positioning them as promising leads for further optimization as dual-acting antidiabetic/neuroprotective agents.

The α-amylase and α-glucosidase inhibitory activities of the thiazole derivatives revealed significant potential for antidiabetic applications. Compounds **5** (3,4-diCl) and **3** (4-SO_2_CH_3_) showed the highest inhibitory activity against α-amylase (21.2 ± 2.7 µM and 24.0 ± 3.1 µM, respectively) and α-glucosidase (17.0 ± 2.5 µM and 18.5 ± 2.9 µM, respectively), outperforming other derivatives in the series. These findings suggest that hydrophobic and electron-withdrawing substituents enhance the enzyme binding affinity, likely through hydrogen bonding and π–π stacking interactions within the enzyme active site. Conversely, compounds with electron-donating groups such as 4-OCH_3_ (compound **4**) and 4-OH (compound **6**) exhibited lower inhibitory potency, highlighting the effect of substituent polarity on enzyme recognition. Remarkably, all synthesized compounds were considerably more active than acarbose, the reference antidiabetic drug (α-amylase IC_50_ = 62.92 ± 1.84 µM; α-glucosidase IC_50_ = 271.92 ± 1.50 µM), indicating that this thiazole scaffold has promising potential for further development as dual-target antidiabetic agents.

The data collectively indicate that substitution patterns on the aromatic ring influence both cholinesterase and carbohydrate-hydrolyzing enzyme inhibition. Electron-withdrawing and di-substituted derivatives (e.g., 4-SO_2_CH_3_, 3,4-diCl) generally showed higher potency across the tested enzymes, whereas electron-donating groups tended to reduce activity. However, given the limited size of the series and the absence of quantitative electronic parameter correlations, these observations should be considered a preliminary SAR trend rather than a generalizable rule, and further studies with an expanded compound series and quantitative SAR modeling are warranted to validate this trend.

### 2.3. Molecular Docking

**Figure 1:**
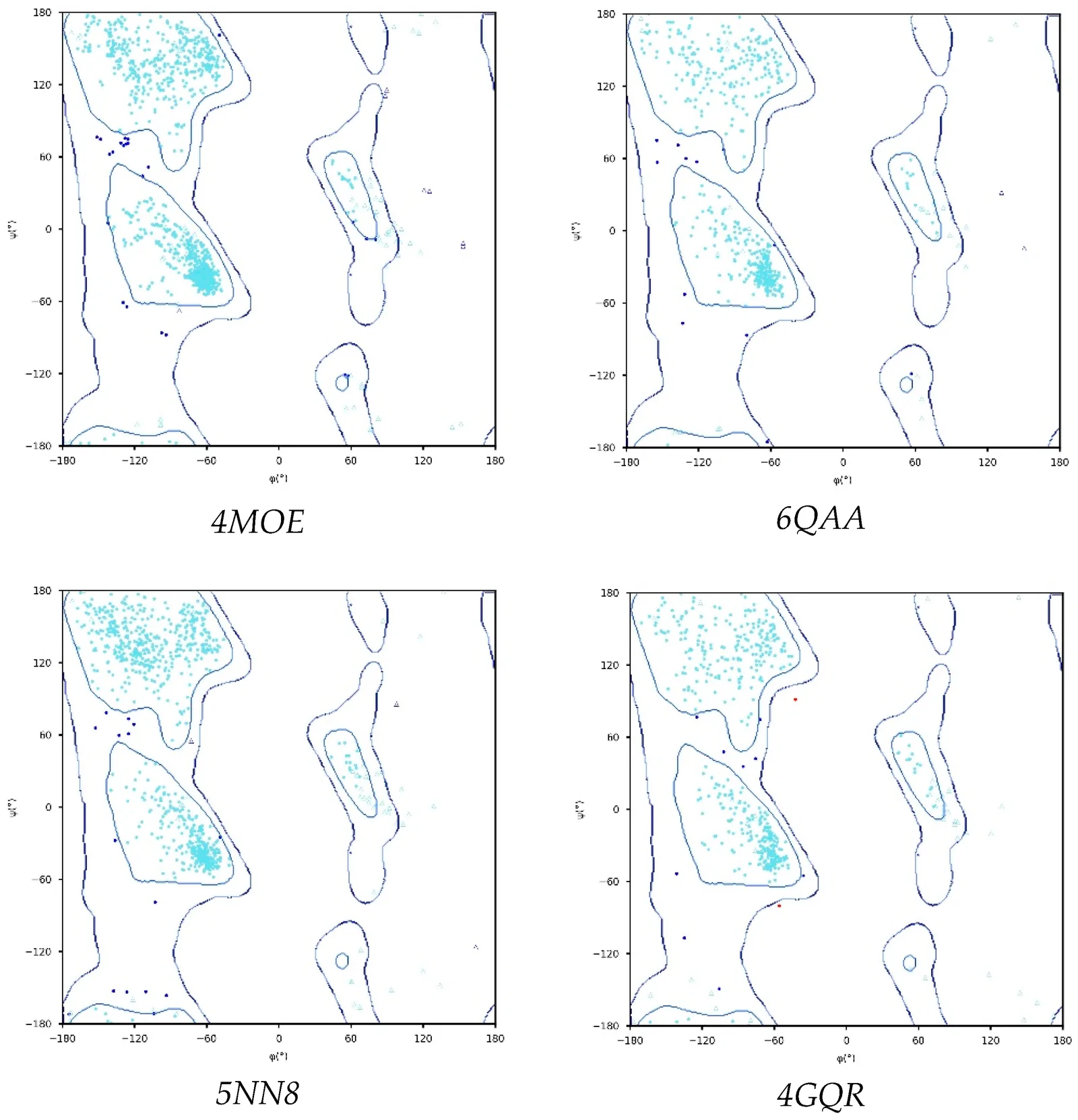
Ramchandran plot of retrieved from Ramplot server.

The docking study of 4M0E, 6QAA, 5NN8, and 4GQR was carried out for the compounds using AutoDock Vina v1.2.7 (Table 3 and Figure 1). The docking results were analyzed in terms of binding energy affinity and the type of interactions at the active site of the selected target (Table 4 and Figure S47). The docking protocol was validated by a redocking experiment of co-crystal ligands, with RMSD between 0.18 and 2.08 Å for all the target proteins, supporting the reliability of docking parameters selected for test compound docking.

### 2.4. Molecular Dynamics Studies

**Figure 2:**
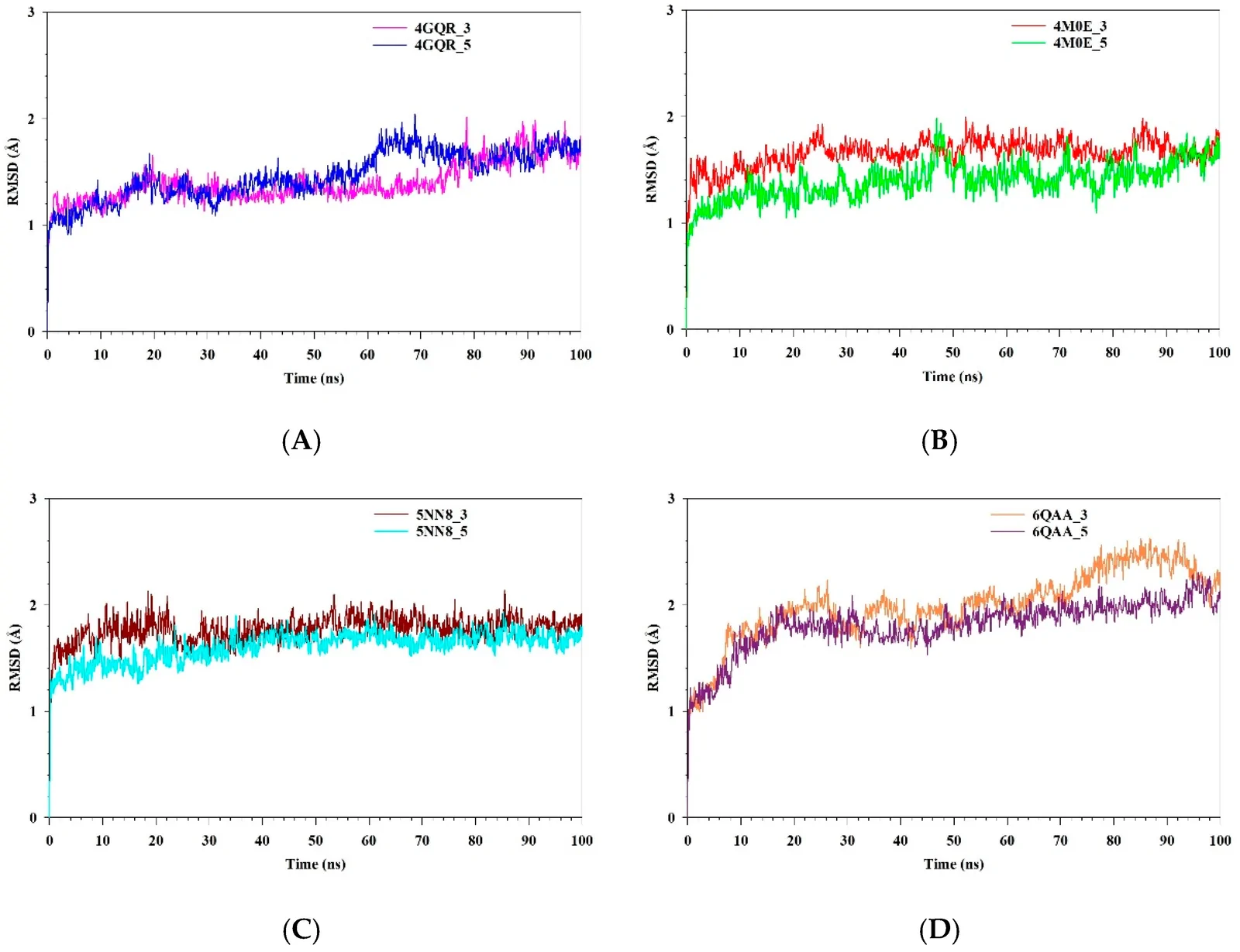
MD simulation analysis of 100 ns trajectories of the RMSD of the Cα backbone of (**A**) 4GQR_**3** (pink), 4GQR_**5** (blue); (**B**) 4M0E_**3** (red), 4M0E_**5** (Green); (**C**) 5NN8_**3** (DK red), 5NN8_**5** (cyan); (**D**) 6QAA_**3** (orange), 6QAA_**5** (Dark purple).

**Figure 3:**
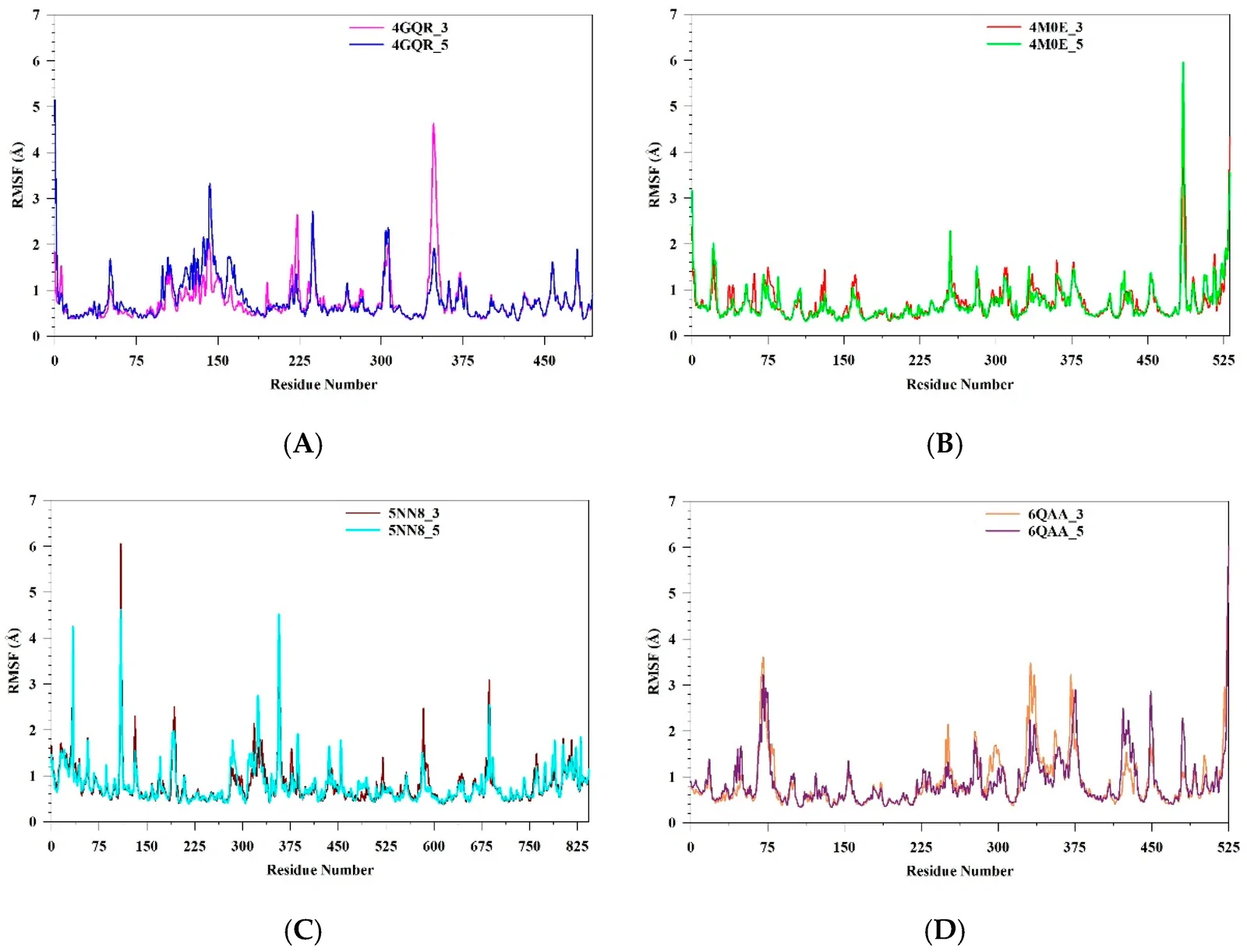
MD simulation analysis of 100 ns trajectories of the RMSF of the Cα backbone of (**A**) 4GQR_**3** (pink), 4GQR_**5** (blue); (**B**) 4M0E_**3** (red), 4M0E_**5** (Green); (**C**) 5NN8_**3** (DK red), 5NN8_**5** (cyan); (**D**) 6QAA_**3** (orange), 6QAA_**5** (Dark purple).

**Figure 4:**
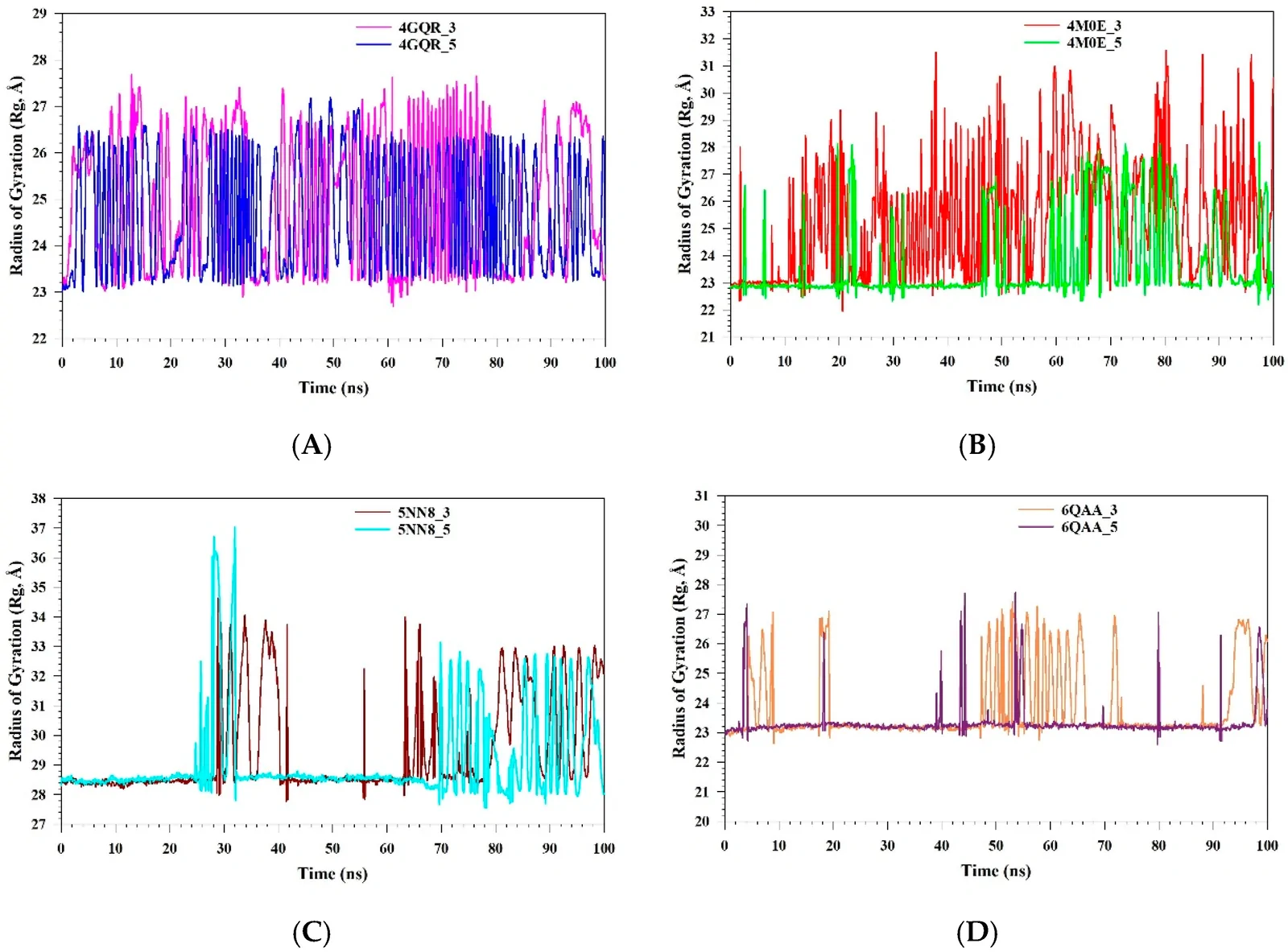
MD simulation analysis of 100 ns trajectories of the Radius of Gyration (Rg) of the Cα backbone of (**A**) 4GQR_**3** (pink), 4GQR_**5** (blue); (**B**) 4M0E_**3** (red), 4M0E_**5** (Green); (**C**) 5NN8_**3** (DK red), 5NN8_**5** (cyan); (**D**) 6QAA_**3** (orange), 6QAA_**5** (Dark purple).

**Figure 5:**
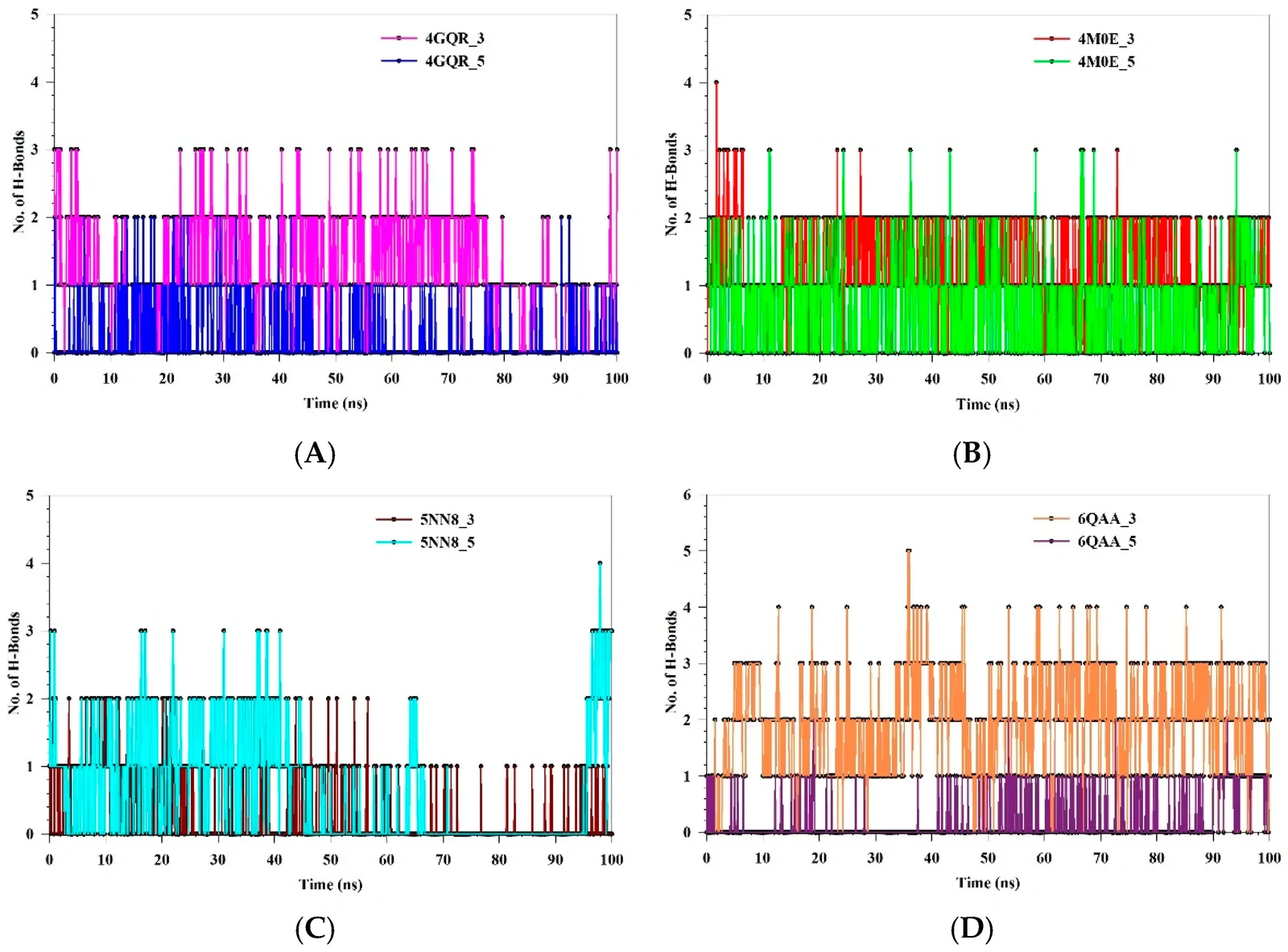
Formation of hydrogen bonds in (**A**) 4GQR_**3** (pink), 4GQR_**5** (blue); (**B**) 4M0E_**3** (red), 4M0E_**5** (Green); (**C**) 5NN8_**3** (DK red), 5NN8_**5** (cyan); (**D**) 6QAA_**3** (orange), 6QAA_**5** (Dark purple).

**Figure 6:**
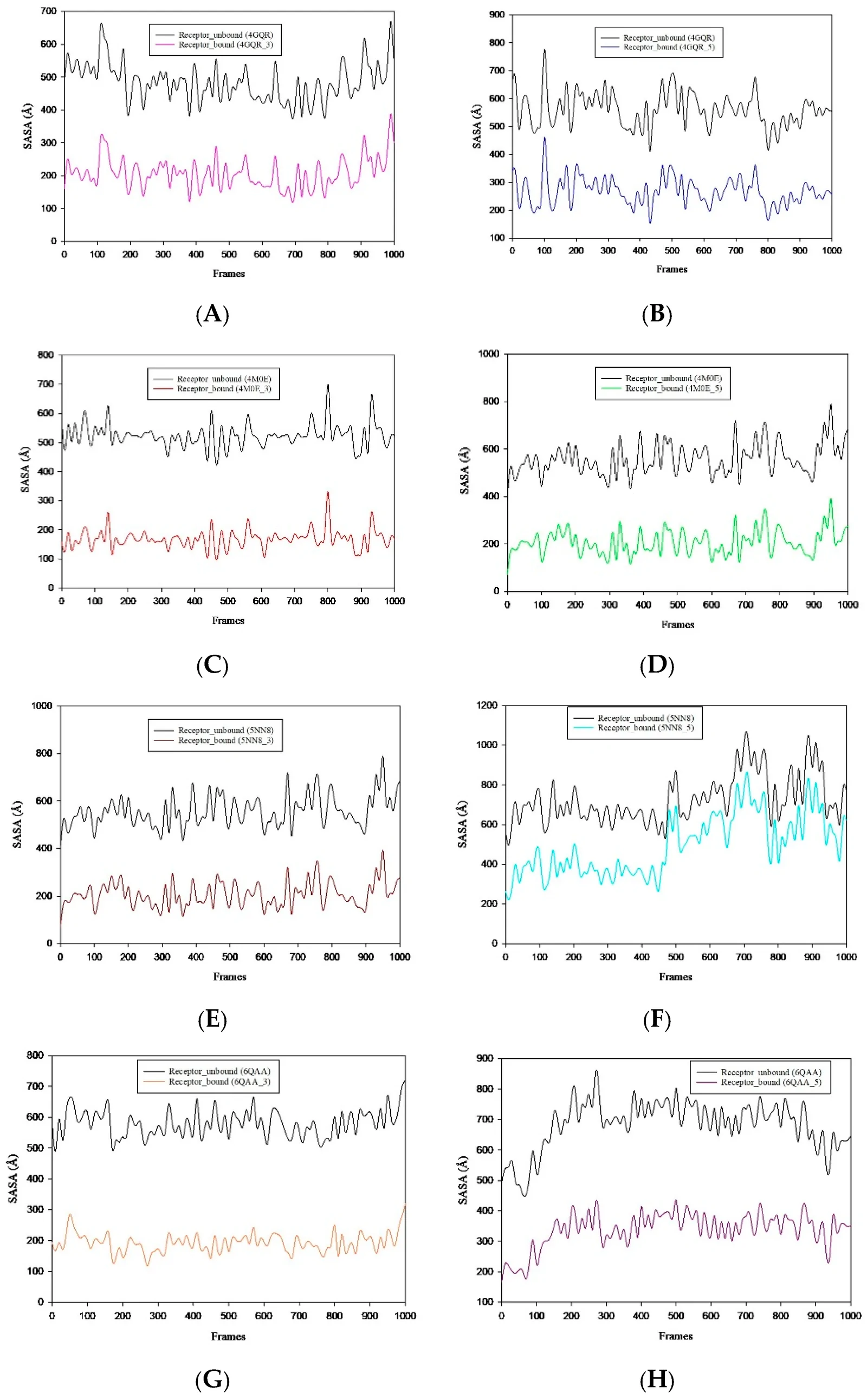
MD simulation analysis of 100 ns time scale of Solvent accessible surface area of (**A**) 4GQR_**3** (pink); (**B**) 4GQR_**5** (blue) (**C**) 4M0E_**3** (red); (**D**) 4M0E_**5** (Green); (**E**) 5NN8_**3** (DK red); (**F**) 5NN8_**5** (cyan); (**G**) 6QAA_**3** (orange); (**H**) 6QAA_**5** (Dark purple).

Molecular dynamics (MD) simulations were performed to evaluate the stability and convergence of 4GQR, 4M0E, 5NN8, and 6QAA with compounds **3** and **5**. Figure 2 represents a Root Mean Square Deviation (RMSD) analysis performed to evaluate the structural behavior of the 4GQR, 4M0E, 5NN8, and 6QAA systems. For the 4GQR systems, both 4GQR_**3** (pink) and 4GQR_**5** (blue) initially fluctuated around 1.0–1.3 Å and gradually increased to approximately 1.4–1.5 Å within the first 20 ns. From about 20 to 55 ns, both trajectories remained relatively consistent, although 4GQR_**3** generally displayed slightly lower RMSD values. A noticeable conformational adjustment was observed for 4GQR_**5** between approximately 58 and 70 ns, during which its RMSD increased to nearly 1.8–2.0 Å. The trajectory subsequently settled near 1.6–1.8 Å. In comparison, 4GQR_**3** remained close to 1.3–1.5 Å for most of the middle segment and increased moderately after 75 ns. The 4M0E_**3** system (red) showed RMSD values mainly between 1.4 and 1.8 Å throughout the trajectory. A gradual increase was observed during the first 25 ns, after which the system fluctuated around a relatively constant level without sustained structural drift. The occasional peaks approaching 2.0 Å were transient and were followed by a return to the established range. The 4M0E_**5** (green) trajectory initially maintained lower RMSD values of approximately 1.0–1.3 Å and subsequently increased to around 1.3–1.6 Å. A temporary rise near 45–50 ns reached approximately 1.9 Å, suggesting a short-lived conformational rearrangement. After this period, the RMSD returned to a lower range before gradually increasing near the end. Overall, 4M0E_**5** exhibited a smaller deviation from its starting structure than 4M0E_**3** for most of the simulation, although both systems remained dynamically stable. For 5NN8, the 5NN8_**3** trajectory (DK red) increased rapidly from approximately 1.3 Å to around 1.7–1.9 Å during the initial 10–20 ns. Following a brief reduction near 25 ns, the RMSD remained predominantly between 1.7 and 1.9 Å for the remainder of the simulation. The absence of a continuous upward trend after equilibration indicates that the system maintained a consistent conformational state. The 5NN8_**5** (cyan) system displayed comparatively lower RMSD values, beginning near 1.2 Å and gradually stabilizing at approximately 1.5–1.7 Å. Although several short-term fluctuations were present, the trajectory remained well controlled and did not show pronounced structural drift. Thus, 5NN8_**5** demonstrated slightly greater conformational consistency than 5NN8_**3**, while both systems retained stable structures throughout the 100 ns period. The 6QAA systems exhibited comparatively larger structural deviations. Both 6QAA_**3** (orange) and 6QAA_**5** (dark purple) increased from approximately 1.0 Å to nearly 1.7–1.9 Å within the first 15 ns, indicating a more substantial initial reorganization. Between 15 and 70 ns, the trajectories fluctuated mainly within the range of 1.7–2.1 Å. After approximately 70 ns, 6QAA_**3** underwent a further increase and reached approximately 2.3–2.5 Å, where it remained for a considerable portion of the later trajectory. This behavior suggests that 6QAA_**3** adopted a new conformational state during the final third of the simulation. In contrast, 6QAA_**5** remained mainly around 1.8–2.0 Å and showed only a moderate increase toward approximately 2.1–2.2 Å near the end. Therefore, 6QAA_**5** displayed better structural consistency than 6QAA_**3**. Figure 3 represents the RMSF profiles, which indicated that most residues in all systems fluctuated below approximately 1.0 Å, suggesting that the protein cores remained structurally stable during the simulation. In 4GQR, the 4GQR_**3** (pink) and 4GQR_**5** (blue) largely overlapped, although the 4GQR_**3** system showed a prominent peak near residues 348–350 with an average RMSF of 0.77 Å, while the 4GQR_**5** system displayed higher flexibility around residues 145 and 235 with an RMSF of 0.80 Å. For 4M0E, the 4M0E_**3** (red) and 4M0E_**5** (green) profiles were comparable across most residues. A major peak near residue 490 was more pronounced in 4M0E_**5**, whereas 4M0E_**3** showed greater fluctuation in the C-terminal region with an RMSF of 0.73 Å. In 5NN8, the 5NN8_**3** (DK red) and 5NN8_**5** (Cyan) showed several localized peaks, particularly near residues 35, 110, 355, and 685. The 5NN8_**3** curve exhibited the highest fluctuation near residue 110, while the 5NN8_**5** curve was more flexible around residue 355. The 6QAA systems, represented by 6QAA_**3** (orange) and 6QAA_**5** (dark purple), showed similar overall patterns but greater flexibility in several loop and terminal regions. The 6QAA_**3** system displayed higher peaks around residues 70, 330, and 375, whereas the 6QAA_**5** system showed greater fluctuations between residues 420–480 and at the C-terminal end. Overall, the elevated RMSF values were restricted to specific flexible regions, while most residues remained comparatively stable. Figure 4 shows that the Radius of Gyration (Rg) showed different levels of compactness among the simulated systems. In 4GQR, the 4GQR_**3** (pink) and 4GQR_**5** (blue) systems fluctuated mainly between approximately 23.02 and 27.62 Å, indicating repeated but comparable changes in protein compactness. For 4M0E, the 4M0E_**5** (green) remained closer to 23 Å for longer periods, whereas the 4M0E_**3** (red) frequently increased to approximately 25–31 Å. This suggests that 4M0E_**5** maintained a comparatively more compact structure. In 5NN8, both the 5NN8_**3** (DK red) and 5NN8_**5** (cyan) curves remained near 28.5 Å during several intervals but showed substantial increases up to approximately 34–37 Å. These variations indicate notable conformational expansion, particularly for 5NN8_**5** during the early-middle trajectory. The 6QAA systems remained predominantly around 23 Å. The 6QAA_**3** (orange) displayed more frequent increases than the 6QAA_**5** (Dark purple), suggesting that 6QAA_**5** generally retained better compactness. Overall, 4M0E_**5** and 6QAA_**5** showed comparatively more consistent compact structures, while 5NN8 exhibited the greatest variation in radius of gyration. Figure 5 represents the hydrogen-bond profiles, which showed continuous but dynamic protein–ligand interactions during the 100 ns simulations. In 4GQR, the 4GQR_**3** (pink) generally maintained 1–2 hydrogen bonds, with occasional formation of 3 bonds, whereas the 4GQR_**5** (blue) mostly formed 0–1 bond. In 4M0E, both the 4M0E_**3** (red) and 4M0E_**5** (green) systems commonly maintained 1–2 hydrogen bonds, although 4M0E_**3** occasionally reached 4 bonds. For 5NN8, the 5NN8_**3** (Dk red) predominantly showed 0–1 hydrogen bond, while the 5NN8_**5** (cyan) formed up to 2–3 bonds during several intervals and reached 4 bonds near the end. The 6QAA_**3** (orange) displayed the strongest hydrogen-bonding pattern, generally maintaining 1–3 bonds and occasionally reaching 4–5 bonds. In contrast, the 6QAA_**5** (Dark purple) mainly formed 0–1 bond. Overall, 6QAA_**3** exhibited the most persistent hydrogen-bond network, whereas 4GQR_**5** and 6QAA_**5** showed comparatively fewer hydrogen-bond contacts. Figure 6 represents the Solvent Accessible Surface Area (SASA) analysis, which showed that all ligand-bound receptors had considerably lower solvent-accessible surface areas than their corresponding unbound receptors. The 4M0E_**3** (red) and 6QAA_**3** (orange) complexes showed particularly low and relatively stable bound-state SASA profiles, indicating effective surface burial and limited conformational expansion. The 4GQR_**3** (pink) and 5NN8_**3** (DK red) systems also maintained lower SASA values than their corresponding compound **5** complexes. In contrast, 5NN8_**5** (cyan) displayed the highest bound-state SASA and the greatest temporal variation, indicating increased solvent accessibility and substantial conformational flexibility during the second half of the trajectory. Based on the SASA profiles, compound **3** generally produced more compact complexes than compound **5** for the four receptors.

**Figure 7:**
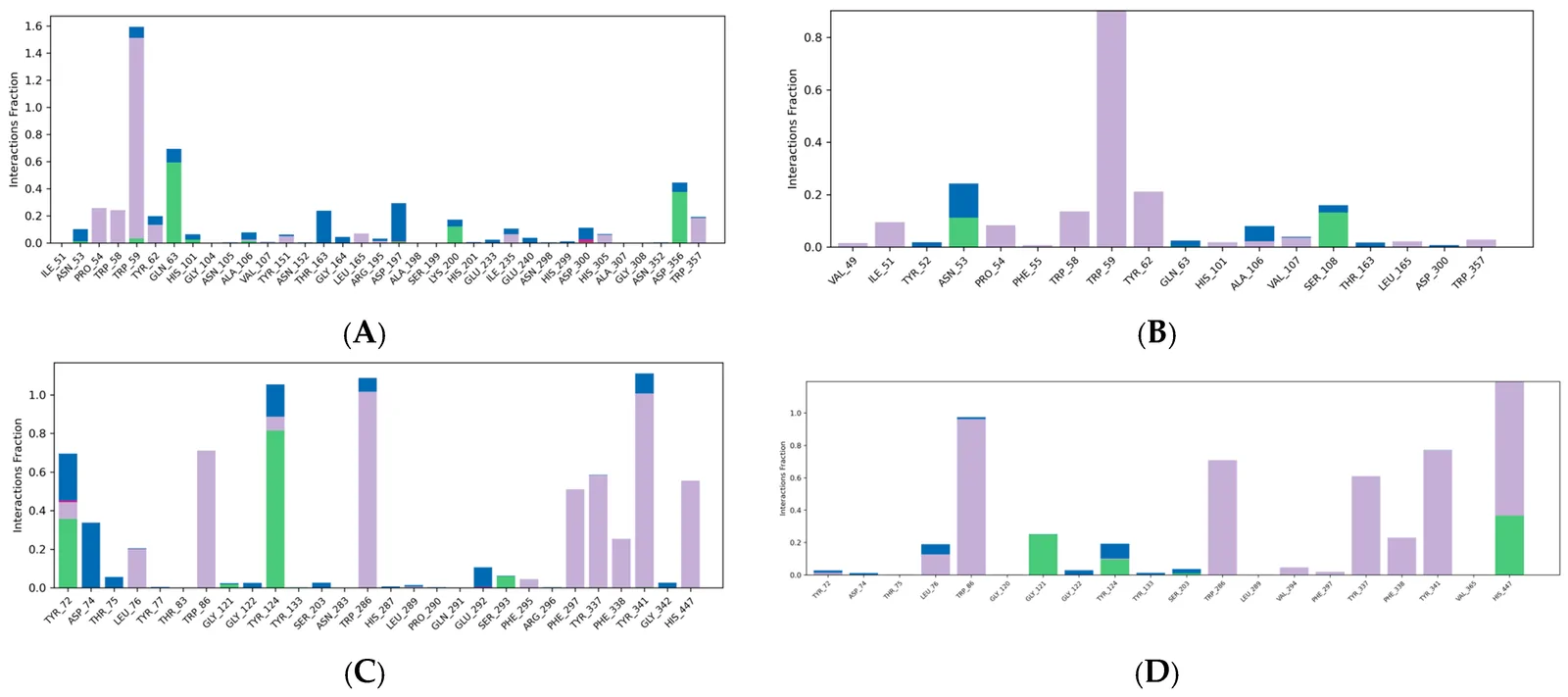

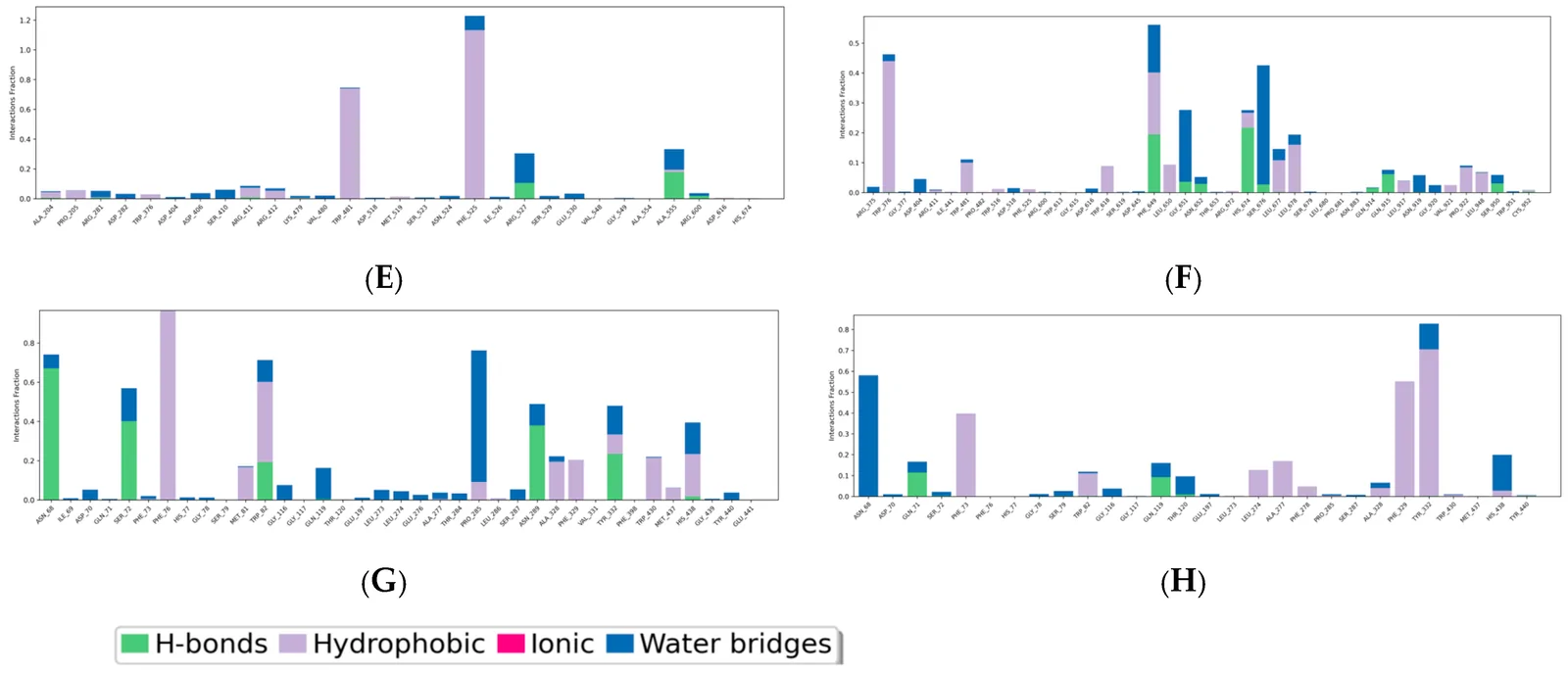
Bar graph of Protein-ligand contacts of (**A**) 4GQR_**3**, (**B**) 4GQR_**5**, (**C**) 4M0E_**3**, (**D**) 4M0E_**5**, (**E**) 5NN8_**3**, (**F**) 5NN8_**5** (cyan), (**G**) 6QAA_3, (**H**) 6QAA_**5**.

The dynamic interactions between the protein and ligand complexes were monitored throughout the 100 ns simulation. These interactions are summarized as normalized stacked bar charts (Figure 7A–H), categorized into hydrogen bonds, hydrophobic interactions, ionic interactions, and water bridges. In instances where a residue maintained multiple contacts of a specific type, the interaction fraction value remains ≥1. The analysis reveals that the stability of the 4GQR, 4M0E, 5NN8, and 6QAA complexes is primarily driven by high interaction fractions in the form of hydrophobic and hydrogen bond interactions.

**Figure 8:**
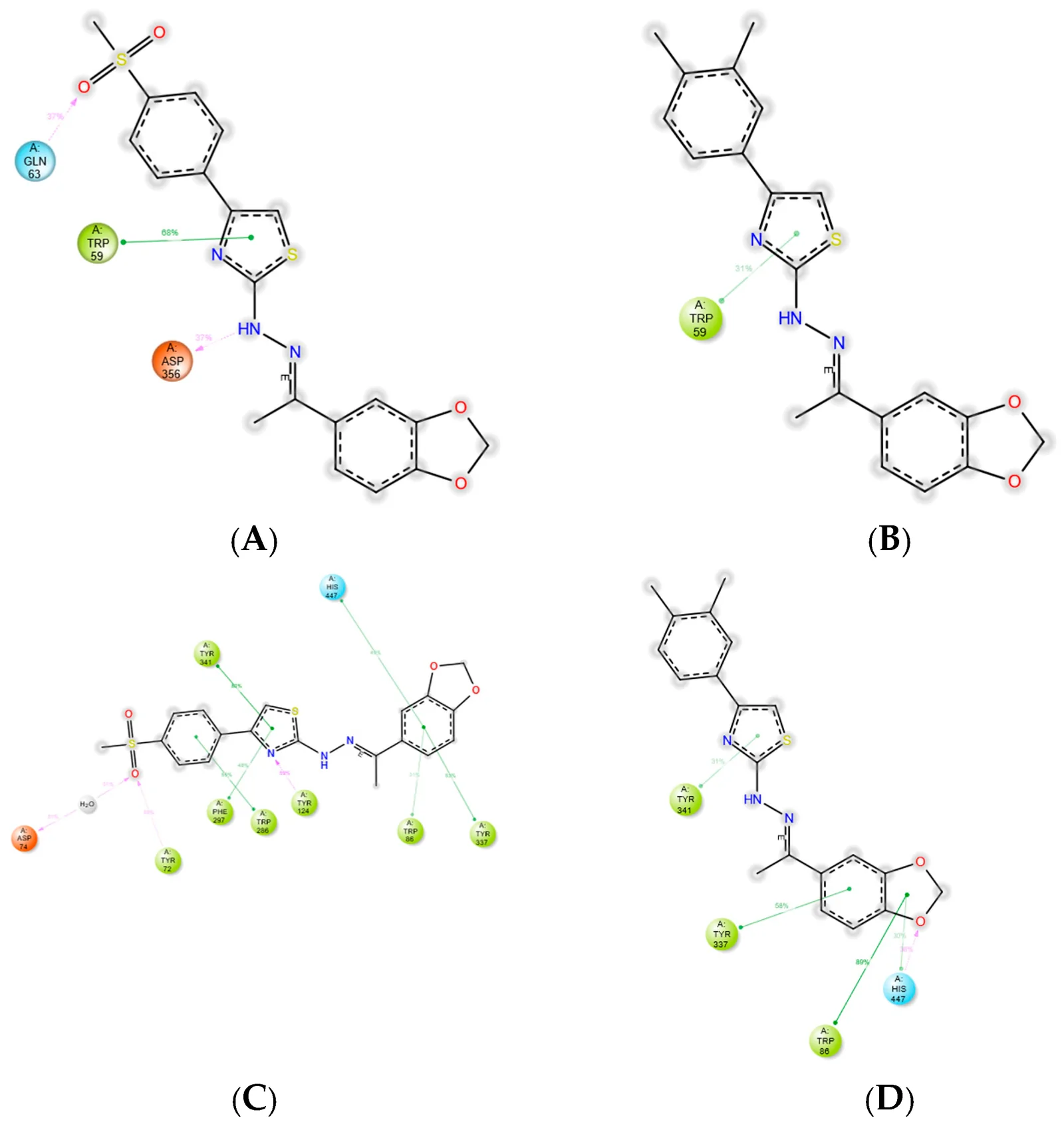

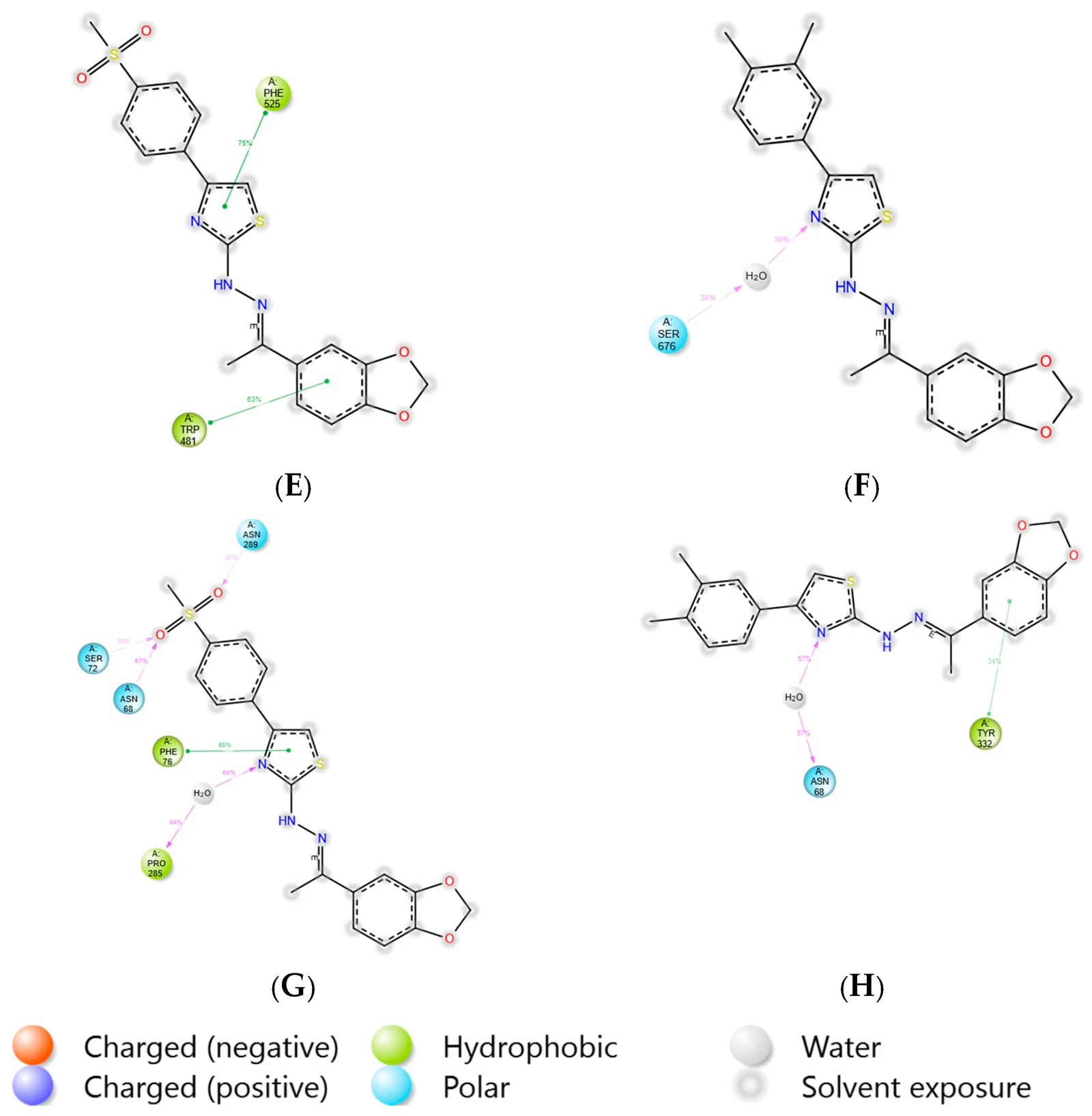
Protein–ligand percent interactions of (**A**) 4GQR_**3**, (**B**) 4GQR_**5**, (**C**) 4M0E_**3**, (**D**) 4M0E_**5**, (**E**) 5NN8_**3**, (**F**) 5NN8_**5** (cyan), (**G**) 6QAA_**3**, (**H**) 6QAA_**5**.

Figure 8 illustrates the percentage of protein–ligand interactions. In 4GQR_**3**, GLN63 (37%) and ASP356 (37%) interact via hydrogen bonding, whereas TRP59 (68%) interacts via hydrophobic interaction. In 4GQR_**5**, TRP59 (31%) interacts via hydrophobic interaction. In 4M0E_**3**, TYR337 (53%), TRP86 (31%), TYR341 (80%), TRP286 (55%), and PHE297 (48%) interact via hydrophobic interactions; TYR72 (33%) and TYR124 (59%) interact via hydrogen bonding. ASP74 (62%) interacts via a water bridge. In 4M0E_**5**, TYR341 (31%), TYR337 (58%), TRP86 (89%), and HIS447 (30%) interact via hydrophobic interactions. In 5NN8_**3**, PHE525 (75%) and TRP481 (63%) interact via hydrophobic interactions. In 5NN8_**5**, SER676 (78%) interacts via a water bridge. In 6QAA_**3**, SER72 (36%), ASN68 (67%), and ASN289 (37%) interact via hydrogen bonding. PHE76 (65%) and PRO285 (128%) interact via hydrophobic and water-bridge interactions, respectively. In 6QAA_**5**, ASN68 (114%) and TYR332 (34%) interact via a water bridge and hydrophobic interactions, respectively.

### 2.5. Molecular Mechanics Generalized Born Surface Area (MM-GBSA) Calculations

Utilizing the MD simulation trajectory, the binding free energy, along with other contributing energies in the form of MM-GBSA, was determined for complexes 4GQR, 4M0E, 5NN8, and 6QAA with compounds **3** and **5**. The results in Table 5 indicate favorable binding energies for all eight complexes. 5NN8_**3** exhibited the strongest binding affinity with a ΔG_bind_ of −203.19 kcal/mol, followed by 5NN8_**5** at −184.08 kcal/mol. Their strong binding was mainly supported by Van der Waals, Coulombic, lipophilic, and hydrogen-bonding contributions, although the positive SolvGB term opposed complex formation.

Among the remaining systems, 4M0E_**3** showed the most favorable binding energy (−77.12 kcal/mol), followed by 6QAA_**3**, 4M0E_**5**, 4GQR_**5**, 6QAA_**5**, and 4GQR_**3**. For the 6QAA systems, 6QAA_**3** showed a more favorable ΔG_bind_ of −53.19 kcal/mol, compared with −43.39 kcal/mol for 6QAA_**5**. Within the 4GQR, 4GQR_**5** demonstrated stronger binding with a ΔG_bind_ of −46.20 kcal/mol, whereas 4GQR_**3** produced a value of −36.02 kcal/mol. The binding of 4GQR_**5** was improved. The results identify 5NN8_**3** as the most energetically favorable complex, followed by 5NN8_**5** and 4M0E_**3**. Compound **3** was preferred in the 4M0E, 5NN8, and 6QAA systems, whereas compound **5** showed superior binding in the 4GQR system.

### 2.6. ADMET Prediction

Potent compounds can only proceed to further testing and be marketed as drugs if they possess acceptable ADMET properties. In this context, certain ADMET properties of the thiazole derivatives (**1**-**9**) were evaluated using the ADMETlab program. Caco-2 permeability, PAMPA, and HIA values were used as the basis for evaluating absorption. Caco-2 permeability and HIA values, which are measures of oral bioavailability, indicate that the compounds (except compound **3**) have high absorption properties. The PAMPA values, another measure of membrane permeability, demonstrated that the compounds possess high permeability.

The volume of distribution (VD) values are optimal for the compounds (except for compound **3**). Compounds **3**, **4**, **6**, and **9** are predicted to have high penetration of the blood-brain barrier (BBB). CYP enzymes, which are responsible for the metabolism of compounds, play a critical role in drug–drug interactions and dose adjustment [19]. In this context, the synthesized compounds are predicted to be inhibitors of CYP3A4 and CYP2C9. On the other hand, while all synthesized compounds are substrates of CYP3A4, it has been determined that only compounds **6** and **9** may be substrates of CYP2C9.

Since the CLplasma values indicating elimination are below 5, the compounds exhibit low plasma clearance, and their T_1_/_2_ values indicate short half-lives. The human ether-a-go-go-related gene (hERG) values, which are an indicator of cardiotoxicity, suggest that the compounds are unlikely to act as hERG blockers. Based on AMES mutagenicity and carcinogenicity data, it is estimated that the compounds may pose a toxicity risk, while the risk of hepatotoxicity is not high, except for compounds **3**, **7**, and **8** (Table 6).

The Lipinski rule—one of the drug-likeness properties used to evaluate the absorption and permeability of an orally administered drug (MW ≤ 500; logP ≤ 5; HAcceptor ≤ 10; HDonor ≤ 5; at most one of these parameters may fall outside the specified range)—provides important insights. It has been determined that the thiazole derivatives (**1**-**9**) comply with the Lipinski rule.

**Figure 9:**
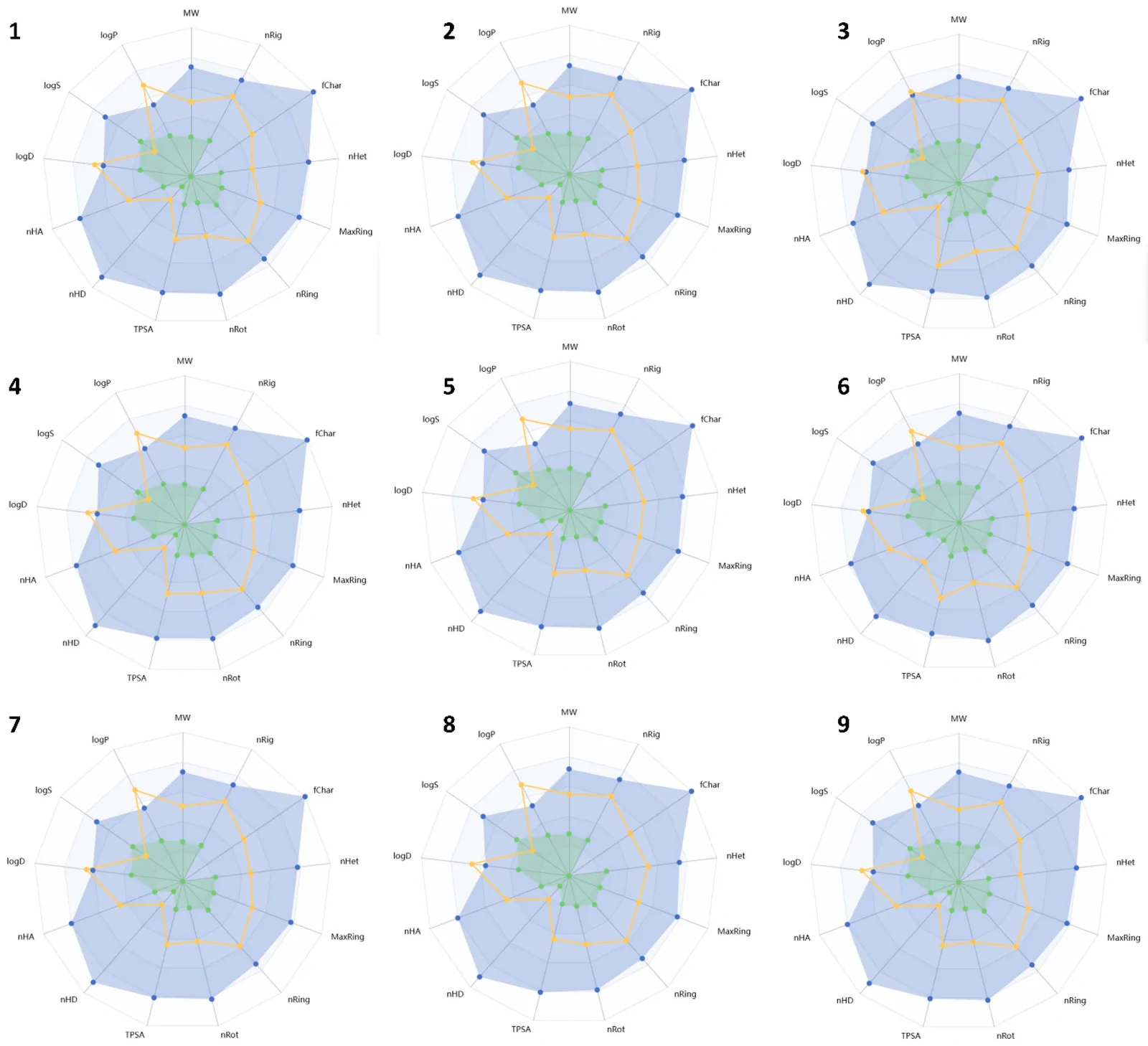
The radar view of the thiazole derivatives (**1**-**9**).

A radar view is a representation derived from certain parameters of compounds, indicating the quality of their physicochemical properties. A compound’s radar image is derived from physicochemical properties such as molecular weight, number of rigid bonds, formal charge, number of heteroatoms, number of atoms in the largest ring, number of rings, number of rotatable bonds, topological polar surface area, number of hydrogen bond donors, number of hydrogen bond acceptors, distribution coefficient, partition coefficient, and water solubility coefficient. It is expected that the orange shape, specific to the compound’s properties, will align with the blue and green shapes [20]. As can be seen from the radar plot, all physicochemical properties except the partition coefficient (log P) fall within the appropriate range (Figure 9). The slight deviation in the partition coefficient is minimal and, as mentioned above, is in compliance with Lipinski’s rule.

The dual-activity approach used in this study is supported by the findings of recent literature. In a similar approach to compounds **3** and **5** here, Sajitha et al. designed and synthesized thiazole–piperazine sulphonamide hybrids that bear both anticholinesterase and antioxidant activities, with docking and MD-based RMSD stability analysis and ADMET screening [21]. In a similar fashion, derivatives of thiazoles were tested in parallel for both cholinesterase inhibition and antioxidant activity, and they were found to be promising anti-Alzheimer’s leads, in agreement with the trends observed in the present series, which are driven by the electron-withdrawing substituents [22]. In an antidiabetic application, compounds of the type 1,2,3-triazole–indole–thiazolidinedione hybrids that combine α-glucosidase, α-amylase and DPP-4 inhibitory activity with antioxidant activity were also tested and validated by docking and MD simulation, showing that dual/multi-target enzyme inhibition with radical-scavenging activity can be rationally combined in a single heterocyclic structure [23] and further confirming the design rationale and SAR trends observed for compounds **3** and **5** of the present invention. Compared with these systems, the thiazole–benzodioxole scaffold reported here shows a similar inhibitory profile (AChE, BChE, α-amylase, α-glucosidase) and antioxidant activity in one compact, structurally simple series, thus exhibiting potential as a compact multi-target lead to be optimized.

## 3. Materials and Methods

### 3.1. General Information

All chemicals and solvents were obtained from Sigma-Aldrich (Merck KgaA, Darmstadt, Germany). Thin-layer chromatography (TLC) was used to monitor the reactions in the synthesis studies. In TLC, petroleum ether:ethyl acetate (1:1, *v*/*v*) was preferred as the mobile phase, and silica-coated aluminum was preferred as the stationary phase. The melting points of the compounds were obtained using a Schmelzpunktbestimmer SMP II instrument (BÜCHI Labortechnik GmbH, Essen, Germany). IR spectra of the compounds were obtained with an FTIR-8400S Shimadzu Spectrometer (Shimadzu Corporation, Kyoto, Japan). NMR spectra of the compounds were recorded with a Bruker Avance III HD 600 MHz (Bruker, Karlsruhe, Germany). For NMR, the compounds were dissolved in deuterodimethyl sulfoxide and recorded against the TMS internal reference standard. LC-MS analyses of the synthesized compounds were performed using an Agilent 1260 Infinity II LC-MS system equipped with a G7115A 1260 DAD WR detector, a G7311B 1260 Quad Pump system, a G1328C 1260 Manual Injection unit, and a G6125B LC/MSD detector. An ACE C18 Column (Avantor, Radnor, PA, USA) was used as the stationary phase. acetonitrile/water (90:10, *v*/*v*) was used as the mobile phase under isocratic elution conditions. The samples were injected separately into the system, and molecular ion peaks were identified by their mass-to-charge ratios (*m*/*z*) in the mass spectrometry section. All ions were detected at their *m*/*z* values using the MSD detector in Scan mode. LC-HRMS spectra of the compounds were obtained with a Thermo Scientific Exactive Plus Benchtop Full-Scan Orbitrap Mass Spectrometer LC-MS/MS analyzer (Thermo Fisher Scientific, San Jose, CA, USA).

### 3.2. Chemistry

For the synthesis of the thiazole compounds (**1**-**9**), the thiosemicarbazone derivative and phenyl bromides were first reacted. For this purpose, 1-(benzo[*d*][1,3]dioxol-5-yl)ethan-1-one (10 mmol, 1.6 g) was dissolved in ethanol (20–25 mL), and thiosemicarbazide (10 mmol, 0.9 g) was added. The mixture was refluxed at 95 °C for 10 h. The resulting solid was filtered and dried (Yield: 80%). Meanwhile, the acetophenone derivatives (10 mmol) were dissolved in ether (20 mL), and a catalytic amount of ALCl_3_ was added. Bromine (12 mmol) was added to the mixture at 0 °C, and the mixture was stirred until the solution became colorless. The ether was evaporated to yield phenacyl bromides (Yield: 70–80%).

Finally, 2-(1-(benzo[*d*][1,3]dioxol-5-yl)ethylidene)hydrazine-1-carbothioamide (1 mmol, 0.2 g) and the phenacyl bromides (1 mmol) were refluxed at 95 °C for 10 h in 15 mL of ethanol and a catalytic amount of glacial acetic acid [24]. The resulting solid was filtered, dried, and then recrystallized from ethanol. Finally, the thiazole derivatives (**1**-**9**) were obtained in pure form.

Compound **2** was previously synthesized by Rauf et al., and its anti-HIV activity has been tested [25]. However, all other compounds are original and have not been reported in the literature.

*2-{2-[1-(Benzo[d][1,3]dioxol-5-yl)ethylidene]hydrazinyl}-4-(p-tolyl)thiazole* (**1**)

Cream-colored solid, yield: 94%, m.p. (with decomposition) > 210 °C. IR (v_max_, cm^−1^): 3219 (N-H); 3064 (Csp^2^-H); 2972, 2902 (Csp^3^-H); 1608 (C=N); 1504, 1435 (aromatic C=C); 1257, 1030 (dioxole C-O-C); 767 (C-S-C). ^1^H NMR (600 MHz, DMSO-*d*_6_) *δ* 12.06 and 11.78 (2s, 1H, N-H), 8.35–6.90 (m, 8H, Csp^2^-H), 6.25–6.02 (m, 2H, O-CH_2_-O), 2.78–2.08 (m, 6H, 2CH_3_). ^13^C NMR (150 MHz, decoupled, DMSO-*d*_6_) *δ* 169.2 (thiazole C-2), 148.5, 148.2 (C=N), 138.1, 132.5, 129.4, 129.1, 128.7, 127.9, 125.2, 123.9, 120.9, 108.5, 107.9, 106.0 (thiazole C-5), 102.5, 101.8 (O-CH_2_-O), 21.3 (C_6_H_4_CH_3_-*p*), 14.7 (CH_3_). HRMS (ESI): *m/z* calcd for C_19_H_18_N_3_O_2_S [M + H]^+^: 352.1120; found: 352.1105.

*2-{2-[1-(Benzo[d][1,3]dioxol-5-yl)ethylidene]hydrazinyl}-4-(4-chlorophenyl)thiazole* (**2**)

Cream-colored solid, yield: 77%, m.p. (with decomposition) > 235 °C. IR (v_max_, cm^−1^): 3308 (N-H); 3055 (Csp^2^-H); 2902, 2798 (Csp^3^-H); 1608 (C=N); 1502, 1435 (aromatic C=C); 1257, 1030 (dioxole C-O-C); 773 (C-S-C). ^1^H NMR (600 MHz, DMSO-*d*_6_) *δ* 12.06 and 11.73 (2s, 1H, N-H), 8.32–6.88 (m, 8H, Csp^2^-H), 6.27–6.01 (m, 2H, O-CH_2_-O), 2.79–2.09 (m, 3H, CH_3_). ^13^C NMR (150 MHz, decoupled, DMSO-*d*_6_) *δ* 169.4, 167.1 (thiazole C-2), 148.6, 148.1 (C=N), 132.9, 132.6, 130.5, 129.7, 128.9, 128.6, 120.9, 119.0, 108.8, 108.4, 106.0 (thiazole C-5), 102.4, 101.8 (O-CH_2_-O), 16.1, 14.7 (CH_3_). HRMS (ESI): *m/z* calcd for C_18_H_15_ClN_3_O_2_S [M + H]^+^: 372.0574; found: 372.0560.

*2-{2-[1-(Benzo[d][1,3]dioxol-5-yl)ethylidene]hydrazinyl}-4-[4-(methylsulfonyl)phenyl]thiazole* (**3**)

Cream-colored solid, yield: 91%, m.p. (with decomposition) > 250 °C. IR (v_max_, cm^−1^): 3232 (N-H); 3007 (Csp^2^-H); 2912, 2798 (Csp^3^-H); 1622 (C=N); 1485, 1438 (aromatic C=C); 1251, 1033 (dioxole C-O-C); 761 (C-S-C). ^1^H NMR (600 MHz, DMSO-*d*_6_) *δ* 11.78 and 11.56 (2s, 1H, N-H), 8.17–6.95 (m, 8H, Csp^2^-H), 6.26–6.03 (m, 2H, O-CH_2_-O), 3.35–3.19 (m, 3H, SO_2_CH_3_), 2.79–2.08 (m, 3H, CH_3_). ^13^C NMR (150 MHz, decoupled, DMSO-*d*_6_) *δ* 170.6, 169.4 (thiazole C-2), 148.7, 148.1 (C=N), 140.4, 140.1, 138.8, 132.6, 132.4, 132.2, 131.6, 129.3, 128.9, 128.0, 127.6, 127.4, 126.5, 120.9, 120.7, 108.4, 108.0, 106.0, 105.9 (thiazole C-5), 102.5, 101.8 (O-CH_2_-O), 44.1, 44.0 (SO_2_CH_3_), 14.8, 14.6 (CH_3_). HRMS (ESI): *m/z* calcd for C_19_H_18_N_3_O_4_S_2_ [M + Na]^+^: 438.0558; found: 438.0556.

*2-{2-[1-(Benzo[d][1,3]dioxol-5-yl)ethylidene]hydrazinyl}-4-(4-methoxyphenyl)thiazole* (**4**)

Orange-colored solid, yield: 76%, m.p. (with decomposition) > 220 °C. IR (v_max_, cm^−1^): 3213 (N-H); 3053 (Csp^2^-H); 2899, 2729 (Csp^3^-H); 1606 (C=N); 1487, 1435 (aromatic C=C); 1259, 1031 (dioxole C-O-C); 748 (C-S-C). ^1^H NMR (600 MHz, DMSO-*d*_6_) *δ* 8.52–6.98 (m, 8H, Csp^2^-H), 6.21–6.07 (m, 2H, O-CH_2_-O), 4.01–3.71 (m, 3H, OCH_3_), 2.62–2.25 (m, 3H, CH_3_). ^13^C NMR (150 MHz, decoupled, DMSO-*d*_6_) *δ* 165.8 (thiazole C-2), 160.1, 151.0, 148.3 (C=N), 135.1, 132.6, 123.9, 123.3, 113.6, 111.7, 108.8, 107.1 (thiazole C-5), 102.4 (O-CH_2_-O), 57.4 (OCH_3_), 16.8, 16.1 (CH_3_). LC-MS (ESI): *m/z* calcd for C_19_H_18_N_3_O_3_S [M + H]^+^: 368.1; found: 368.3.

*2-{2-[1-(Benzo[d][1,3]dioxol-5-yl)ethylidene]hydrazinyl}-4-(3,4-dichlorophenyl)thiazole* (**5**)

Yellow-colored solid, yield: 95%, m.p. (with decomposition) > 240 °C. IR (v_max_, cm^−1^): 3234 (N-H); 3041 (Csp^2^-H); 2904, 2783 (Csp^3^-H); 1616 (C=N); 1487, 1435 (aromatic C=C); 1253, 1033 (dioxole C-O-C); 756 (C-S-C). ^1^H NMR (600 MHz, DMSO-*d*_6_) *δ* 12.03 and 11.76 (2s, 1H, N-H), 8.44–6.94 (m, 7H, Csp^2^-H), 6.28–6.04 (m, 2H, O-CH_2_-O), 2.58–2.09 (m, 3H, CH_3_). ^13^C NMR (150 MHz, decoupled, DMSO-*d*_6_) *δ* 165.3 (thiazole C-2), 151.2, 148.3 (C=N), 136.7, 132.0, 131.9, 130.7, 130.1, 124.1, 122.1, 108.8, 107.2 (thiazole C-5), 102.4 (O-CH_2_-O), 16.1 (CH_3_). HRMS (ESI): *m/z* calcd for C_18_H_14_Cl_2_N_3_O_2_S [M + H]^+^: 406.0184; found: 406.0205.

*4-{2-[2-(1-(Benzo[d][1,3]dioxol-5-yl)ethylidene]hydrazinyl}-4-(4-hydroxyphenyl)thiazole* (**6**)

Orange-colored solid, yield: 95%, m.p. (with decomposition) > 238 °C. IR (v_max_, cm^−1^): 3444 (O-H); 3323 (N-H); 3111 (Csp^2^-H); 2997, 2852 (Csp^3^-H); 1608 (C=N); 1505, 1435 (aromatic C=C); 1251, 1031 (dioxole C-O-C); 744 (C-S-C). ^1^H NMR (600 MHz, DMSO-*d*_6_) *δ* 12.09 and 11.81 (2s, 1H, N-H), 8.47–6.97 (m, 8H, Csp^2^-H), 6.25–6.03 (m, 2H, O-CH_2_-O), 2.79–2.08 (m, 3H, CH_3_). ^13^C NMR (150 MHz, decoupled, DMSO-*d*_6_) *δ* 167.8, 166.5 (thiazole C-2), 163.7, 151.9, 150.8, 148.3 (C=N), 133.6, 132.1, 131.2, 128.4, 125.2, 123.7, 120.5, 116.8, 108.7, 107.9, 107.0 (thiazole C-5), 103.6, 102.3 (O-CH_2_-O), 16.7, 16.0 (CH_3_). HRMS (ESI): *m/z* calcd for C_18_H_16_N_3_O_3_S [M + H]^+^: 354.0912; found: 354.0894.

*2-{2-[1-(Benzo[d][1,3]dioxol-5-yl)ethylidene]hydrazinyl}-4-(4-fluorophenyl)thiazole* (**7**)

Cream-colored solid, yield: 88%, m.p. (with decomposition) > 243 °C. IR (v_max_, cm^−1^): 3223 (N-H); 3059 (Csp^2^-H); 2902, 2752 (Csp^3^-H); 1612 (C=N); 1504, 1435 (aromatic C=C); 1259, 1031 (dioxole C-O-C); 765 (C-S-C). ^1^H NMR (600 MHz, DMSO-*d*_6_) *δ* 12.09 and 11.81 (2s, 1H, N-H), 8.38–6.96 (m, 8H, Csp^2^-H), 6.27–6.02 (m, 2H, O-CH_2_-O), 2.79–2.08 (m, 3H, CH_3_). ^13^C NMR (150 MHz, decoupled, DMSO-*d*_6_) *δ* 168.6, 166.9 (thiazole C-2), 154.7, 151.0, 148.9, 148.3 (C=N), 133.7, 130.9, 128.4, 126.0, 124.0, 116.7, 115.4, 108.8, 107.1 (thiazole C-5), 102.4, 101.8 (O-CH_2_-O), 16.1, 14.7 (CH_3_). HRMS (ESI): *m/z* calcd for C_18_H_15_FN_3_O_2_S [M + H]^+^: 356.0869; found: 356.0849.

*2-{2-[1-(Benzo[d][1,3]dioxol-5-yl)ethylidene]hydrazinyl}-4-[4-(trifluoromethyl)phenyl]thiazole* (**8**)

Yellow-colored solid, yield: 80%, m.p. (with decomposition) > 257 °C. IR (v_max_, cm^−1^): 3215 (N-H); 3051 (Csp^2^-H); 2899, 2708 (Csp^3^-H); 1606 (C=N); 1500, 1433 (aromatic C=C); 1257, 1028 (dioxole C-O-C); 750 (C-S-C). ^1^H NMR (600 MHz, DMSO-*d*_6_) *δ* 12.08 and 11.53 (2s, 1H, N-H), 8.47–6.89 (m, 8H, Csp^2^-H), 6.28–6.02 (m, 2H, O-CH_2_-O), 2.79–2.08 (m, 3H, CH_3_). ^13^C NMR (150 MHz, decoupled, DMSO-*d*_6_) *δ* 169.6, 169.4 (thiazole C-2), 148.7, 148.1 (C=N), 132.3, 131.5, 129.3, 129.2, 129.1, 128.9, 128.4, 125.8, 125.6, 120.9, 120.9, 108.4, 106.0 (thiazole C-5), 101.8 (O-CH_2_-O), 14.8, 14.7 (CH_3_). HRMS (ESI): *m/z* calcd for C_19_H_15_F_3_N_3_O_2_S [M + H]^+^: 406.0837; found: 406.0816.

*2-{2-[1-(Benzo[d][1,3]dioxol-5-yl)ethylidene]hydrazinyl}-4-([1,1′-biphenyl]-4-yl)thiazole* (**9**)

Yellow-colored solid, yield: 87%, m.p. (with decomposition) > 245 °C. IR (v_max_, cm^−1^): 3223 (N-H); 3028 (Csp^2^-H); 2908, 2779 (Csp^3^-H); 1614 (C=N); 1485, 1448 (aromatic C=C); 1251, 1031 (dioxole C-O-C); 746 (C-S-C). ^1^H NMR (600 MHz, DMSO-*d*_6_) *δ* 8.45–7.03 (m, 13H, Csp^2^-H), 6.26–6.03 (m, 2H, O-CH_2_-O), 2.63–2.12 (m, 3H, CH_3_). ^13^C NMR (150 MHz, decoupled, DMSO-*d*_6_) *δ* 163.7 (thiazole C-2), 156.4, 151.1, 148.8 (C=N), 140.3, 136.8, 135.7, 133.6, 131.6, 129.7, 127.5, 118.4, 108.7, 107.1 (thiazole C-5), 102.4, 102.0 (O-CH_2_-O), 16.1 (CH_3_). HRMS (ESI): *m/z* calcd for C_24_H_20_N_3_O_2_S [M + H]^+^: 414.1276; found: 414.1263.

### 3.3. Biological Activity

For all bioactivity assays (antioxidant, anticholinesterase, and antidiabetic), stock solutions of the pure synthesized compounds were prepared in DMSO, and serial dilutions were prepared to obtain final concentrations of 100, 50, 25, 12.5, 6.25, and 3.125 µM in the assay plate (i.e., in the final reaction mixture). All biological experiments were carried out in triplicate. DMSO was used as a negative control to monitor the reaction. The corresponding DMSO concentrations in the final reaction mixtures ranged from 0.5% to 1.0% (*v*/*v*), and the DMSO vehicle was used as the control. Appropriate blanks containing all assay components except the test compounds were included, and the absorbance values obtained from the blanks were subtracted from the corresponding sample measurements to correct for background absorbance. The bleaching rate was calculated from the absorbance differences versus time. The sample concentration providing 50% inhibition activity (IC_50_) for all other assays, and 0.5 absorbance (A_0.5_) for the CUPRAC assay, were calculated from the graph of bleaching rate (%) against sample concentrations. All biological activity measurements were performed using a 96-well microplate reader (SpectraMax 340PC^384^, Molecular Devices, San Jose, CA, USA).

### 3.4. In Vitro Antioxidant Activity

The antioxidant activity of the thiazole derivatives (**1**-**9**) was determined using three complementary assays, namely, 2,2-diphenyl-1-picrylhydrazyl (DPPH) free radical scavenging activity, 2,2′-azino-bis(3-ethylbenzothiazoline-6-sulfonic acid) (ABTS) cation radical scavenging activity, and cupric reducing antioxidant capacity (CUPRAC). Butylated hydroxyanisole (BHA) was used as a standard for comparison of activity.

### 3.5. Determination of DPPH Free Radical Scavenging Activity

The DPPH free radical scavenging activity was performed according to Blois (1958) [26], with slight modifications. Briefly, 160 μL of 0.004% DPPH in ethanol was mixed with 40 μL of the thiazole derivatives (**1**-**9**) dissolved in ethanol at different concentrations. The absorbance was measured at 517 nm after 30 min incubation in the dark.

### 3.6. Determination of ABTS Cation Radical Scavenging Activity

The ABTS cation radical scavenging activity was performed according to Re et al. (1999) [27], with slight modifications [28]. The ABTS cation radical was prepared using the reaction between 7 mM ABTS in water and 2.45 mM potassium persulfate and then stored in the dark at room temperature for 12 h. The formed ABTS cation radical solution was diluted to a 1:88 ratio with ethanol before use to obtain an absorbance of 0.700 ± 0.025 at 734 nm. Then, briefly, 160 μL of the prepared reagent was mixed with 40 μL of the thiazole derivatives (**1**-**9**) dissolved in DMSO at different concentrations. The absorbance was measured at 734 nm after 10 min incubation.

### 3.7. Determination of Cupric Reducing Antioxidant Capacity

Cupric reducing antioxidant capacity (CUPRAC) assay was performed according to Apak et al. (2004) [29]. Briefly, 50 μL 10 mM Cu (II), 50 μL 7.5 mM neocuproine, and 60 μL NH_4_Ac buffer (1 M, pH 7.0) were added to each well in a 96-well plate. Add 40 μL of the thiazole derivatives (**1**-**9**) at various concentrations to the mixture. After 1 h, incubation absorbance was recorded at 450 nm.

### 3.8. In Vitro Enzyme Inhibitory Activities

Galantamine for AChE and BChE, and acarbose for α-amylase and α-glucosidase were used as positive standards to compare inhibitory activity. All enzymes used in the bioactivity assays were purchased from Sigma-Aldrich (St. Louis, MO, USA).

### 3.9. Determination of Anticholinesterase Activity

The electric eel acetylcholinesterase (AChE, Type-VI-S, EC 3.1.1.7, 10KU) and horse serum butyrylcholinesterase (BChE, EC 3.1.1.8, 6KU) were used to determine the anticholinesterase activity in DMSO of the thiazole derivatives (**1**-**9**), where acetylthiocholine iodide and butyrylthiocholine chloride were employed as substrates using a spectroscopic method [30]. Briefly, 130 μL sodium phosphate buffer (100 mM, pH 8.0), 10 μL of the thiazole derivatives (**1**-**9**) at different concentrations, and 20 μL AChE (5.32 × 10^−3^ U/20 μL) or BChE (6.85 × 10^−3^ U/20 μL) enzymes in buffer were mixed. After incubation for 15 min at 25 °C, 20 μL of 0.5 mM DTNB (5,50-dithiobis (2-nitrobenzoic acid) and 20 μL of acetylthiocholine iodide (0.71 mM) or butyrylthiocholine chloride (0.2 mM) were added. Then the absorbance was measured at 412 nm.

### 3.10. Determination of α-Amylase Inhibitory Activity

α-Amylase inhibitory activity of the thiazole derivatives (**1**-**9**) was tested by using the spectroscopic method with slight changes from Quan et al. (2019) [31]. Briefly, 25 µL of sample solution at different concentrations and 50 µL α-amylase from porcine pancreas (Type VI-B, EC 3.2.1.1, 0.1 U/mL) in phosphate buffer (20 mM, pH = 6.9; phosphate buffer prepared with 6 mM NaCl) were mixed in a 96-well microplate. The mixture was pre-incubated for 10 min at 37 °C. After pre-incubation, 50 µL starch solution (0.05%) was added and incubated for more 10 min at 37 °C. The reaction was stopped by addition of 25 µL HCl (0.1 M), and then 100 µL Lugol solution was added for monitoring. 96-well microplate reader was used to measure absorbance at 565 nm.

### 3.11. Determination of α-Glucosidase Inhibitory Activity

α-Glucosidase inhibitory activity of the thiazole derivatives (**1**-**9**) was determined using the spectroscopic method with slight modifications [32]. Briefly, 50 µL phosphate buffer (10 mM pH = 6.9), 25 µL PNPG (*p*-nitrophenyl-*α*-*D*-glucopyranoside) in phosphate buffer (10 mM pH = 6.9), 10 µL sample solution, and 25 µL *Saccharomyces cerevisiae* α-glucosidase (Type I, EC 3.2.1.20, 0.1 U/mL) in phosphate buffer (10 mM pH = 6.0) were mixed in a 96-well microplate. After 20 min incubation at 37 °C, 90 µL sodium carbonate (100 mM) was added to each well to stop the enzymatic reaction. Absorbance of the 96-well microplate reader was recorded at 400 nm.

### 3.12. Calculation of IC_50_ Values

For each assay, the percentage inhibition was calculated at each tested concentration using the formula: % Inhibition = [(A₍control₎ − A₍sample₎)/A₍control₎] × 100, where A₍control₎ and A₍sample₎ represent the absorbance values of the control (DMSO blank) and the test compound, respectively. IC_50_ values, defined as the concentration required to inhibit 50% of the enzymatic activity or radical-scavenging capacity, were determined by plotting percentage inhibition against the logarithm of compound concentration and fitting the data with non-linear regression.

### 3.13. Molecular Docking

In this study, we selected the X-ray crystallographic structures of the target proteins acetylcholinesterase (PDB ID: 4M0E), butyrylcholinesterase (PDB ID: 6QAA), lysosomal acid-α-glucosidase (PDB ID: 5NN8), pancreatic_α-amylase (PDB ID: 4GQR), which were retrieved from the RCSB Protein Data Bank, accessed on 12 March 2026 (https://www.rcsb.org). While selecting proteins for docking studies, the PDB entry was validated using the X-ray crystallographic method for resolution (<3 Å), wwPDB validation (better), and Ramachandran plot (residues in allowed region > 88%) [33]. The target protein refinement was performed using CHIMERA V1.16 [34] to ensure high-quality input for the docking study, where standard residues present in the protein were minimized using the AMBER force field, and non-standard residues were minimized using AM1-BCC semi-empirical charges to maintain proper electrostatic interactions. Non-standard residues like water, co-crystal ligands, and unnecessary chains were removed, which may interfere with the docking procedure. Structures of compounds were drawn in Marvin Sketch; explicit hydrogens were added, then optimized for 2D and 3D cleanliness; possible conformers were generated, from which the lowest-energy conformer was selected and saved in mol2 format. Then, structures were optimized in CHIMERA using the AM1-BCC force field. Proteins and ligands were converted to PDBQT format using AutoDockTools 4.2.6 [35], which can be read by AutoDock Vina 1.2.7 [26]. In AutoDockTools, the rotatable bonds in the ligands were identified and made flexible during the docking study to allow conformational variability. The protein was kept rigid, while the ligand’s flexibility was explicitly considered through flexible rotatable bonds. It is important to note that docking was performed without explicit solvent conditions. Active site amino acids were identified for target protein 5D41 using the co-crystallized ligand as a reference; the amino acids present in the active sites are shown in Table 7.

The Grid box was defined around the active site, and the grid dimensions were optimized to encompass the protein–ligand interactions. According to the active site, grid centers were defined, as shown in Table 8.

The docking protocol was meticulously designed to reflect insights into interactions between protein-ligand complexes. We used AutoDock Vina V. 1.2.7 for molecular docking [36]. The docking parameters were optimized by multiple docking runs using various grid sizes and based on different tools to identify the best binding pose and replicable results, such as spacing (0.375 Å), num_modes (9), energy range (3), exhaustiveness (16), and other default configurations. Docking results were visualized through the Biovia Discovery Studio visualization tool and Maestro 13.9 (Academic Edition) [37] for structural analysis, and binding interactions were analyzed using the Protein–Ligand Interaction Profiler PLIP, accessed on 12 March 2026, https://plip-tool.biotec.tu-dresden.de/plip-web/plip/index [38]. To validate the docking protocol, the co-crystal ligands of each target (1YL in 4M0E, HUN in 6QAA, Acarbose in 5NN8, MYC in 4GQR) were redocked in the same binding pocket, and RMSDs were calculated, which confirms the reliability of the docking protocol. Details are available in the Supplementary Materials (Figure S48).

### 3.14. Molecular Dynamics Studies

The Desmond 2025-1 from Schrödinger, LLC was used to run MD simulations on docked complexes 4GQR, 4M0E, 5NN8, and 6QAA with compounds **3** and **5**. Compounds **3** and **5** were chosen for MD simulation because they also exhibited the lowest IC_50_ values for all four biological targets, namely the inhibition of AChE (8.7 and 7.5 µM), BChE (compound **5**, 28.0 µM), α-amylase (24.0 and 21.2 µM), and α-glucosidase (18.5 and 17.0 µM) in the synthesized series (Table 2). To gain deeper insight into the structural stability and binding behavior that leads to the better experimental activity of these two compounds, MD simulations were performed. The OPLS-2005 force field [39,40,41] and an explicit solvent model with SPC water molecules were used in this system [42] in an orthorhombic periodic boundary simulation box of 10 Å × 10 Å × 10 Å dimensions. Na^+^ ions were added to neutralize the charge at 0.15 M, and NaCl solutions were added to the system to simulate the physiological environment. The system was relaxed before production with Desmond’s default multistage equilibration protocol. This consisted of (i) a Brownian dynamics simulation under the NVT ensemble at 10 K with solute heavy atoms restrained (200 ps); (ii) NVT simulation at 10 K with solute heavy atoms restrained (50 ps); (iii) NPT simulation at 10 K with solute heavy atoms restrained (50 ps); (iv) NPT simulation in which the system was heated to 300 K with solute heavy atoms restrained (50 ps); and (v) an unrestrained NPT simulation at 300 K (100 ps), totaling 450 ps of relaxation. The force constant for solute heavy-atom restraints was used during the restrained stages (default: 50 kcal mol^−1^ Å^−2^). This production was then performed with the NPT ensemble at 300 K and 1.01325 bar with the use of the Martyna–Tuckerman–Klein (MTK) barostat (relaxation time 2.0 ps) and the Nose–Hoover thermostat (relaxation time 1.0 ps) [43]. The integrator of the RESPA was used with a bonded/near time step of 2 fs and a far time step of 6 fs. Long-range electrostatic interactions were calculated using a 9 Å short-range cutoff with the u-series method from Desmond, a smooth particle-particle particle-mesh Ewald method. The production run lasted for 100 ns with trajectory frames recorded every 100 ps (1000 frames per trajectory). A time step of 2 fs was used. The Martyna–Tuckerman–Klein chain coupling scheme [44] and barostat method were used for pressure control with a relaxation time of 2 ps. The particle mesh Ewald method [45] was used to calculate long-range electrostatic interactions, and the Coulomb cutoff radius was fixed at 9 Å. The final production run was carried out for 100 ns. The root mean square deviation (RMSD), the radius of gyration (Rg), root mean square fluctuation (RMSF), and several hydrogen bonds (H-bonds) were calculated to monitor the stability of the MD simulations [17,46].

### 3.15. Binding Free Energy Analysis

The molecular mechanics combined with the generalized Born surface area (MM-GBSA) approach [47] was used to compute the binding free energies of the ligand-protein complexes. The Prime MM-GBSA binding free energy was calculated using the Python 3.14.7 script thermal mmgbsa.py in the simulation trajectory for the last 50 frames with a 1-step sampling size. The binding free energy of Prime MM-GBSA (kcal/mol) was estimated using the principle of additivity, in which individual energy modules such as Coulombic, covalent, hydrogen bond, Van der Waals, self-contact, lipophilic, and solvation of protein and ligand were collectively added. The equation used to calculate ΔG_bind_ is the following:

$${\Delta G}_{bind}={\Delta G}_{MM}+{\Delta G}_{Solv}-{\Delta G}_{SA}$$

where:

- Δ*G_bind_* designates the binding free energy,
- Δ*G_MM_* designates the difference between the free energies of ligand-protein complexes and the total energies of protein and ligand in isolated form,
- Δ*G_Solv_* designates the difference in the GSA solvation energies of the ligand-receptor complex and the sum of the solvation energies of the receptor and the ligand in the unbound state,
- Δ*G_SA_* designates the difference in the surface area energies for the protein and the ligand [48].

### 3.16. In Silico ADMET Prediction

The absorption, distribution, metabolism, elimination, and toxicity (ADMET) parameters of 1,3-thiazole compounds (**1**-**9**) were evaluated using ADMETlab 3.0 (https://admetlab3.scbdd.com/, accessed on 5 April 2026). In addition, bioavailability radar plots for the 1,3-thiazole compounds (**1**-**9**) were also generated using this software.

## 4. Conclusions

In the present study, some new thiazole compounds containing a 1,3-benzodioxole ring (**1**-**9**) were synthesized and evaluated for their potential biological activities. Compound **8** with a 4-trifluoromethyl substituent exhibited strong antioxidant activity in both ABTS and DPPH assays. Compound **9**, which has a 4-phenyl substituent, demonstrated high antioxidant activity in both the ABTS and DPPH assays. While the thiazole compounds exhibited weaker ChE inhibition than galantamine, they demonstrated higher α-glucosidase and α-amylase enzyme inhibition than acarbose. Compounds **3** and **5**, with 4-methylsulfonyl and 3,4-dichloro substituents, respectively, exhibited high levels of both anticholinesterase and antidiabetic activity. This study demonstrates that the biological activity of the thiazole–benzodioxole derivatives is influenced by the electronic and steric properties of the aromatic substituents. Electron-withdrawing and di-substituted derivatives generally exhibited enhanced cholinesterase and carbohydrate-hydrolyzing enzyme inhibition, whereas incorporation of a 4-trifluoromethylphenyl substituent was associated withsuperior radical scavenging activity. These structure-activity observations should be regarded as preliminary given the limited series size and warrant further validation with a broader compound set and quantitative SAR analysis. Overall, the structure-activity relationship analysis provides valuable insights for the rational design of dual-function molecules with potent antioxidant and enzyme inhibitory properties, highlighting the potential of this scaffold as a lead for neuroprotective and antidiabetic drug development.

## Figures and Tables

**Table 1:** Antioxidant activities of synthesized thiazoles ^a^.

Compound	Antioxidant Activity		
	ABTS Assay (IC_50_ µM)	DPPH Assay (IC_50_ µM)	CUPRAC Assay (A_0.5_ µM) *
**1**	78.12 ± 0.22	40.23 ± 0.79	71.46 ± 0.00
**2**	45. 95 ± 0.26	45.95 ± 0.26	50. 50 ± 0.00
**3**	64.18 ± 0.62	27.27 ± 0.41	94.53 ± 0.03
**4**	44.15 ± 0.48	23.93 ± 0.18	27.82 ± 0.01
**5**	25.17 ± 0.14	10.09 ± 0.13	86.18 ± 0.01
**6**	11.65 ± 0.08	5.01 ± 0.13	54.89 ± 0.03
**7**	13.90 ± 0.36	6.72 ± 0.01	51.31 ± 0.00
**8**	10.17 ± 0.07	3.11 ± 0.00	69.25 ± 0.01
**9**	9.28 ± 0.48	2.05 ± 0.06	78.62 ± 0.03
**BHA ^b^**	2.49 ± 0.05	5.93 ± 0.20	5.74 ± 0.41

Abbreviation: BHA, 2-tert-Butyl-4-hydroxyanisole. ^a^ Values expressed herein are mean ± SEM of three parallel measurements, *p* < 0.05. ^b^ Reference compound. * A_0.5_: The concentration showing 0.500 absorbance.

**Table 2:** IC_50_ µM results of enzyme inhibition activities of synthesized compounds **1**-**9** ^a^.

Compounds	Anticholinesterase Activity		Antidiabetic Activities	
	AChE Assay	BChE Assay	α-Amylase Assay	α-Glucosidase Assay
**1**	12.5 ± 1.8	45.0 ± 5.3	30.2 ± 4.1	25.0 ± 3.7
**2**	10.2 ± 1.5	38.4 ± 4.7	28.5 ± 3.9	22.7 ± 3.2
**3**	8.7 ± 1.2	31.0 ± 4.0	24.0 ± 3.1	18.5 ± 2.9
**4**	14.0 ± 2.0	50.2 ± 6.0	32.5 ± 4.3	27.8 ± 3.8
**5**	7.5 ± 1.1	28.0 ± 3.6	21.2 ± 2.7	17.0 ± 2.5
**6**	15.2 ± 2.3	52.0 ± 6.5	35.0 ± 4.8	30.0 ± 4.0
**7**	11.0 ± 1.6	40.0 ± 5.0	29.0 ± 3.8	23.5 ± 3.4
**8**	9.0 ± 1.3	33.5 ± 4.2	25.5 ± 3.3	19.8 ± 2.8
**9**	13.5 ± 2.1	48.0 ± 6.0	31.0 ± 4.2	26.5 ± 3.5
**Galantamine ^b^**	1.82 ± 0.30	4.62 ± 0.12	NT	NT
**Acarbose ^b^**	NT	NT	62.92 ± 1.84	271.92 ± 1.50

^a^ Values expressed herein are mean ± SEM of three parallel measurements. *p* < 0.05. NT: not tested. ^b^ Reference compounds.

**Table 3:** Comparison between standard values and retrieved protein for validation of the protein selected for the docking study.

Parameters	Target	Protein Id	Method of Experiment	Mutation	Resolution	wwPDB Validation	Co-Crystal Ligand	Ramchandran Plot (Ramchandran Map Server) Residues in Favored + Allowed Regions
Standards	-	-	X-RAY Diffraction	No	About 3.00 Å	Better	-	>90%
Details	AChE	4M0E	X-RAY Diffraction	No	2	Better	1YL	100%
	BChE	6QAA	X-RAY Diffraction	No	1.9	Better	HUN	100%
	α-Glucosidase	5NN8	X-RAY Diffraction	No	2.45	Better	Acarbose	100%
	Pancreatic α-Amylase	4GQR	X-RAY Diffraction	No	1.2	Better	MYC	98%

**Table 4:** The list of interactions between ligands and proteins 4M0E, 6QAA, 5NN8, and 4GQR.

Name of Complex	Binding Energy (kcal/mol)	Type of Interaction: Residue ID (Distance Å)			
		Hydrophobic Interactions	Hydrogen Bonds	π-Stacking	Salt Bridge/Halogen Bond
4M0E-1	−8.785	TRP286A (3.57), LEU289A (3.72), LEU289A (3.37), GLU292A (3.67), TYR337A (3.76), PHE338A (4), PHE338A (3.56), TYR341A (3.54)	GLU292A (3.31), SER293A (2.39)	TRP286A (4.44), TYR341A (4.28)	-
4M0E-2	−8.746	TRP86A (3.4), TYR341A (3.68), TYR341A (3.72)	ASP74A (3), TYR133A (2.64), TYR337A (1.94)	TRP86A (4.54), TRP286A (4.05), TYR341A (4.08)	-
4M0E-3	−10.363	ASP74A (3.84), TRP86A (3.21), TYR341A (3.61), TYR341A (3.77)	ASP74A (2.74), TYR133A (2.57), TYR337A (2.12)	TRP286A (3.95), TYR341A (3.91)	-
4M0E-4	−8.493	TRP286A (3.66), LEU289A (3.46), GLU292A (3.6), PHE338A (3.72), TYR341A (3.62)	GLU292A (2.87), SER293A (2.22)	TRP286A (4.12), TYR341A (4.29), HIS287A (5.9)	-
4M0E-5	−8.552	PHE297A (3.66), TYR337A (3.69), PHE338A (3.64)	GLY121A (2.62), GLY122A (3.25), TYR337A (3.68), HIS447A (2.38)	TRP286A (3.84)	-
4M0E-6	−9.559	TYR72A (3.32), TRP86A (3.65), TYR337A (3.62), PHE338A (3.57), TYR341A (3.54)	TYR72A (2.2), TYR133A (3.46), TYR337A (2.56)	TRP286A (4.72), TRP286A (3.86)	-
4M0E-7	−9.768	TRP286A (3.99), TRP286A (3.39), LEU289A (3.57), GLU292A (3.96), GLU292A (3.83), TYR341A (3.72), TYR341A (3.62)	-	TRP286A (4.25)	-
4M0E-8	−9.435	TRP286A (3.68), TRP286A (3.46), LEU289A (3.43), GLU292A (3.67), TYR341A (3.52)	SER293A (2.69)	TYR341A (4.28)	-
4M0E-9	−9.892	ASP74A (3.99), TRP86A (3.35), TRP286A (3.87), TYR341A (3.6), TYR341A (3.97)	ASP74A (3), TYR133A (2.54), TYR337A (1.94)	TRP86A (4.48), TRP286A (4), TYR341A (4.07)	-
4M0E-Galantamin	−8.013	TYR72A (3.91), TRP286A (3.58), TRP286A (3.71), LEU289A (3.65)	SER293A (2.36), SER293A (2.6)	TRP286A (4.19), TRP286A (3.77)	-
4M0E-1yl	−10.566	TYR72A (3.61), TRP286A (3.99), TRP286A (3.92), TRP286A (3.96), TYR341A (3.54), TYR341A (3.6)	-	-	-
6QAA-1	−8.697	GLN119A (3.66), GLN119A (3.72), GLU276A (3.38), PHE329A (3.66), TYR332A (3.73), TYR332A (3.83)	PRO285A (2.62)	-	-
6QAA-2	−8.57	GLN119A (3.69), GLN119A (3.67), GLU276A (3.19), PHE329A (3.63), TYR332A (3.77), TYR332A (3.84)	PRO285A (2.65)	-	-
6QAA-3	−8.405	-	THR120A (1.88)	TRP82A (4.02)	
6QAA-4	−8.524	GLN119A (3.65), THR120A (3.66), ALA328A (3.83), PHE329A (3.8)	ASN68A (2.39), GLU276A (3.09), PRO285A (2.1)	TRP82A (5.01), PHE329A (4.99)	-
6QAA-5	−8.383	GLN119A (3.72), GLU276A (3.67), ALA277A (3.3)	THR120A (2.12)	TRP82A (4.09)	-
6QAA-6	−8.572	ASN68A (3.99), GLN119A (3.62)	ASN68A (3.62), THR120A (3.5), THR120A (1.96), GLU276A (3.06), ASN289A (2.21)	TRP82A (4.02)	-
6QAA-7	−8.792	ASN68A (3.95), GLN119A (3.62), GLN119A (3.7), PHE329A (3.61), TYR332A (3.73), TYR332A (3.78)	PRO285A (2.55)	-	-
6QAA-8	−8.987	ASN68A (3.6), ASP70A (4)	ASN68A (2.99)	PHE329A (5.06)	
6QAA-9	−9.071	TRP82A (3.53), GLN119A (3.7), GLU276A (3.82), ALA277A (3.69)	-	TRP82A (4.29), TRP82A (4.26)	-
6QAA-Galantamin	-7.658	TRP82A (3.69), TRP82A (3.7), PHE329A (3.65)	SER198A (2.97)	-	ASP70A (5.02)
6QAA-Hun	−8.095	ASP70A (3.78), TRP82A (3.66), PHE329A (3.6), PHE329A (3.63), PHE398A (3.96)	-	PHE329A (4.99)	-
5NN8_1	−7.493	TRP481A (3.72), TRP481A (3.71), PHE525A (3.93), PHE525A (3.83), PHE649A (3.78)	-	PHE525A (3.7)	-
5NN8_2	−7.67	TRP376A (3.55), TRP376A (3.86), TRP481A (3.89), PHE525A (3.61), ALA555A (3.66), PHE649A (3.63)	ARG600A (3.1), HIS674A (2.79)	-	-
5NN8_3	−7.662	TRP376A (3.74), TRP481A (3.99), TRP481A (3.71), PHE649A (3.71)	ARG600A (2.44)	-	-
5NN8_4	−7.343	TRP376A (3.52), TRP376A (3.8), PHE525A (3.55), ALA555A (3.69), PHE649A (3.67)	ARG600A (2.88), HIS674A (2.81)	-	-
5NN8_5	−7.044	TRP376A (3.75), TRP481A (3.85), TRP618A (3.96), PHE649A (3.68)	ARG600A (2.48)	TRP481A (5.45)	
5NN8_6	−7.2	TRP376A (3.66), TRP618A (3.98), LEU650A (3.95), LEU650A (3.93), LEU678A (3.75)	ASP518A (3.28), GLY651A (2.54)	TRP481A (5.17)	
5NN8_7	−7.534	TRP376A (3.8), TRP376A (3.89), PHE525A (3.97)	-	PHE649A (5.21)	-
5NN8_8	−7.848	TRP376A (3.47), ALA555A (3.77), PHE649A (3.73)	ARG281A (3.34), ASP282A (2.65)	TRP481A (5.1)	ASP518A (3.48)
5NN8_9	**−8.725**	TRP376A (3.72), ALA555A (3.53), PHE649A (3.74)	ALA555A (2.65)	TRP481A (5.21)	
5NN8-Acarbose	−6.057	LEU677A (3.66)	GLY377A (3.03), SER379A (2.38), SER379A (2.72), SER379A (2.06), SER379A (2.03), LEU405A (3.12), ASP406A (3.03), ASP409A (2.31), SER410A (2.14), SER410A (2.69), SER410A (2.29), ARG411A (3.14), ARG411A (3.28), ARG411A (3.08), ARG411A (3.21), ASN417A (3.03), ASP419A (2.51)	-	-
5NN8-Alpha Acarbose	−7.376	TRP376A (3.7)	ARG281A (2.67), ASP282A (2.37), ALA284A (3.39), ASP404A (3.4), ASP404A (3.33), TRP481A (2.67), ARG600A (2), ARG600A (2.48), ARG600A (2.85), ASP616A (2.24), HIS674A (2.27)	-	-
4GQR-1	−7.726	THR163A (2.8), ARG195A (2.3), GLU233A (3.55), HIS299A (2.63)	TRP59A (3.86), TRP59A (3.99), TYR62A (5.09)	-	-
4GQR-2	−7.794	LEU162A (3.62)	-	TRP59A (4.08), TRP59A (3.6)	-
4GQR-3	−7.765	TRP59A (3.63), TRP59A (3.83), TYR62A (3.37), LEU162A (3.99), LEU162A (3.98), LEU165A (3.62), LEU165A (3.79)	-		-
4GQR-4	−7.455	TRP58A (3.83), TYR62A (3.61), GLN63A (3.91), LEU165A (3.89)	GLU233A (3.15)	TRP59A (3.98), TRP59A (4.22), TRP59A (4.57)	-
4GQR-5	−7.959	TRP58A (3.83), TRP59A (3.84), TRP59A (3.85), TYR62A (3.84), ASP300A (3.49)	THR163A (2.71)	TYR62A (5.08)	
4GQR-6	−7.787	TRP59A (3.56), LEU162A (3.77)	GLU233A (2.8), HIS299A (2.65)	TRP59A (3.91), TRP59A (4.47)	-
4GQR-7	−7.643	TRP58A (3.6), TRP58A (3.94), TRP59A (3.58), TYR62A (3.93), TYR62A (3.67), ALA106A (3.87), VAL107A (3.81), LEU165A (3.68)	THR163A (3.54), THR163A (2.6)	-	ASP197A (3.12)
4GQR-8	−8.085	TRP59A (3.51), TRP59A (3.64), TYR62A (3.52), LEU162A (3.8), LEU165A (3.93), ILE235A (3.68)	GLN63A (2.87)	TRP59A (4.09), TYR62A (5.5)	
4GQR-9	**−8.82**	TRP59A (3.79), TRP59A (3.86), LEU162A (3.64), ILE235A (3.47)	GLU233A (3.12)	TRP59A (3.66)	
4GQR-Acarbose	−6.6	--	GLN63A (3.24), ASN105A (3.56), ASN105A (2.85), ALA106A (2.15), ASP197A (3.37), ASP197A (2.69), GLU233A (2.24), HIS299A (2.13)	-	-
4GQR-Alpha Acarbose	−8.553	TRP58A (3.64), TRP59A (3.93), TYR62A (3.82)	GLN63A (1.93), GLU233A (2.46), GLU233A (3), HIS299A (2.15)	TRP59A (4.19), TRP59A (4.39)	

**Table 5:** Binding free energy components for 4GQR, 4M0E, 5NN8, and 6QAA with compounds **3** and **5** were calculated by MM-GBSA.

Energies (kcal/mol)	4GQR_3	4GQR_5	4M0E_3	4M0E_5	5NN8_3	5NN8_5	6QAA_3	6QAA_5
**ΔG_bind_**	−36.02 ± 4.70	−46.20 ± 3.72	−77.12 ± 2.62	−48.49 ± 3.57	−203.19 ± 10.91	−184.08 ± 8.61	−53.19 ± 3.40	−43.39 ± 3.31
**ΔG_bind_Coulomb**	12.76 ± 2.35	−6.27 ± 1.77	8.75 ± 1.28	−7.49 ± 3.45	−130.10 ± 52.32	−32.94 ± 10.32	17.75 ± 3.21	−8.38 ± 1.99
**ΔG_bind_Covalent**	1.82 ± 0.75	2.13 ± 1.00	2.23 ± 1.17	2.26 ± 0.96	4.38 ± 3.71	1.75 ± 4.15	3.34 ± 0.49	2.73 ± 0.63
**ΔG_bind_Hbond**	−0.75 ± 0.28	−0.19 ± 0.13	−1.01 ± 0.22	−0.74 ± 0.39	−9.67 ± 0.99	−7.66 ± 0.47	−0.98 ± 0.38	−0.55 ± 0.16
**ΔG_bind_Lipo**	−11.14 ± 1.31	−18.01 ± 1.33	−31.94 ± 0.76	−25.82 ± 1.68	−41.17 ± 3.08	−38.39 ± 4.80	−18.50 ± 0.75	−18.01 ± 0.96
**ΔG_bind_Packing**	−4.65 ± 1.23	−3.34 ± 0.50	−9.21 ± 0.94	−4.56 ± 0.90	−3.91 ± 0.84	−2.42 ± 1.41	−5.32 ± 0.49	−4.11 ± 0.72
**ΔG_bind_SolvGB**	−4.78 ± 1.91	19.53 ± 1.19	10.00 ± 2.67	35.35 ± 2.53	113.93 ± 51.71	28.76 ± 9.19	2.53 ± 2.37	21.52 ± 1.94
**ΔG_bind_VdW**	−29.26 ± 2.59	−40.05 ± 2.13	−55.94 ± 1.45	−47.50 ± 1.48	−136.66 ± 6.29	−133.19 ± 7.16	−52.01 ± 1.56	−36.58 ± 1.68

**Table 6:** The ADMET properties of the thiazole derivatives (**1**-**9**).

Comp	Absorption			Distribution		Metabolism				Excretion		Toxicity			
	Caco-2	PAMPA	HIA	VD	BBB	CYP3A4 Inh	CYP3A4 Sub	CYP2C9 Inh	CYP2C9 Sub	CL	T_1/2_	hERG	AMES	Car	HHT
**1**	−5.055	0.133	0.0	0.369	0.743	1.0	0.015	1.0	0.749	2.864	0.747	0.213	0.805	0.798	0.697
**2**	−5.010	0.092	0.0	0.341	0.966	1.0	0.056	1.0	0.927	2.512	0.833	0.327	0.687	0.751	0.687
**3**	−5.242	0.662	0.0	−0.026	0.15	0.999	0.001	0.997	0.883	1.083	1.085	0.393	0.656	0.829	0.85
**4**	−5.069	0.069	0.002	0.301	0.154	1.0	0.001	1.0	0.993	3.654	0.634	0.267	0.849	0.87	0.683
**5**	−4.994	0.11	0.0	0.404	0.994	1.0	0.192	1.0	0.968	2.559	0.901	0.38	0.63	0.811	0.7
**6**	−5.075	0.552	0.0	0.141	0.196	1.0	0.0	1.0	0.197	4.125	0.845	0.182	0.801	0.803	0.659
**7**	−5.07	0.054	0.0	0.369	0.952	1.0	0.01	1.0	0.835	2.551	0.84	0.241	0.838	0.869	0.721
**8**	−4.919	0.086	0.0	0.581	0.886	1.0	0.007	0.999	0.969	3.636	0.956	0.336	0.516	0.5	0.806
**9**	−5.05	0.07	0.0	0.347	0.653	1.0	0.003	1.0	0.11	2.693	0.802	0.199	0.815	0.779	0.698

Caco-2 permeability: Optimal: higher than −5.15 Log units. PAMPA: Molecules with log Peff values below 2.0 were classified as low-permeability. HIA: Human intestinal absorption, Category 0: HIA-(HIA >= 30%); Category 1: HIA + (HIA < 30%). VD: Volume distribution, Optimal: 0.04–20 L/kg. BBB: Blood-brain barrier penetration, Category 1: BBB+; Category 0: BBB−. Enzyme inhibitor: Category 1: Inhibitor; Category 0: Non-inhibitor. Enzyme substrate: Category 1: Substrate; Category 0: Non-substrate. CL: The unit of predicted CLplasma penetration is ml/min/kg. >15 mL/min/kg: high clearance; 5–15 mL/min/kg: moderate clearance; <5 mL/min/kg: low clearance. T_1/2_: Half-life of drugs. T_1/2_: Ultra-short half-life drugs < 1 h < short half-life drugs < 4 h < intermediate short half-life drugs < 8 h < long half-life drugs. hERG: Molecules with IC_50_ ≤ 10µM or ≥50% inhibition were classified as hERG+, while molecules with IC_50_ > 10µM or <50% inhibition were classified as hERG-. AMES: AMES toxicity. Category 1: Ames positive(+); Category 0: Ames negative(−). Car: Carcinogenicity. Category 1: carcinogens; Category 0: non-carcinogens. HHT: Human hepatotoxicity. Category 1: H-HT positive(+); Category 0: H-HT negative(−).

**Table 7:** Active Site Amino Acids.

PDB ID	Active Site Amino Acids
4M0E	TYR72A, ASP74A, TYR124A, TRP286A, SER293A, VAL294A, PHE295A, PHE297A, TYR337A, PHE338A, TYR341A
6QAA	ASP70A, THR120A, LEU286A, VAL288A, ALA328A, PHE329A, TRP430A, PRO285A, GLY116A, SER287A, TRP231A, TRP231A, PHE329A
5NN8	TRP376A, TRP481A, PHE649A, ASP282A, ASP518A, ASP518A, ARG600A, ARG600A, ASP616A, HIS674A, HIS674A, TYR292A, ASP616A
4GQR	TYR62A, LEU165A, TRP59A, TYR62A, ASP197A, ALA198A, GLN63A, THR163A, ARG195A, ARG195A, HIS201A, GLU233A

**Table 8:** Grid Parameter.

PDB ID ↓	Center Coordinates			Size Coordinates		
	X	Y	Z	X	Y	Z
4M0E	−17.2	−42.38	25.58	30	30	30
6QAA	18.52	42.38	40.93	20	20	20
5NN8	−13.92	−38.29	95.23	35	35	35
4GQR	13.44	15.14	39.44	20	20	20

## Data Availability

The original contributions presented in this study are included in the article/Supplementary Materials. Further inquiries can be directed to the corresponding authors.

## Supplementary Materials

The following supporting information can be downloaded at: https://www.mdpi.com/article/10.3390/ph19091468/s1, Figure S1. IR spectrum for **1**; Figure S2. 1H-NMR spectrum for **1**; Figure S3. 13C-NMR spectrum for **1**; Figure S4. LC-MS spectrum for **1**; Figure S5. LC-HRMS spectrum for **1**; Figure S6. IR spectrum for **2**; Figure S7. 1H-NMR spectrum for **2**; Figure S8. 13C-NMR spectrum for **2**; Figure S9. LC-MS spectrum for **2**; Figure S10. LC-HRMS spectrum for **2**; Figure S11. IR spectrum for **3**; Figure S12. 1H-NMR spectrum for **3**; Figure S13. 13C-NMR spectrum for **3**; Figure S14. LC-MS spectrum for **3**; Figure S15. LC-HRMS spectrum for **3**; Figure S16. IR spectrum for **4**; Figure S17. 1H-NMR spectrum for **4**; Figure S18. 13C-NMR spectrum for **4**; Figure S19. LC-MS spectrum for **4**; Figure S20. LC-HRMS spectrum for **4**; Figure S21. IR spectrum for **5**; Figure S22. 1H-NMR spectrum for **5**; Figure S23. 13C-NMR spectrum for **5**; Figure S24. LC-MS spectrum for **5**; Figure S25. LC-HRMS spectrum for **5**; Figure S26. IR spectrum for **6**; Figure S27. 1H-NMR spectrum for **6**; Figure S28. 13C-NMR spectrum for **6**; Figure S29. LC-MS spectrum for **6**; Figure S30. LC-HRMS spectrum for **6**; Figure S31. IR spectrum for **7**; Figure S32. 1H-NMR spectrum for **7**; Figure S33. 13C-NMR spectrum for **7**; Figure S34. LC-MS spectrum for **7**; Figure S35. LC-HRMS spectrum for **7**; Figure S36. IR spectrum for **8**; Figure S37. 1H-NMR spectrum for **8**; Figure S38. 13C-NMR spectrum for **8**; Figure S39. LC-MS spectrum for **8**; Figure S40. LC-HRMS spectrum for **8**; Figure S41. IR spectrum for **9**; Figure S42. 1H-NMR spectrum for **9**; Figure S43. 13C-NMR spectrum for **9**; Figure S44. LC-MS spectrum for **9**; Figure S45. LC-HRMS spectrum for **9**; Figure S46. HSQC spectrum for **3**; Figure S47. 2D and 3D interactions between ligands and protein 5D41; Figure S48. Validation of docking protocol through redocking of cocrystal ligand in binding pocket of respective targets. Superimposition of experimental co-crystal ligand pose (Orange) before docking and re-docked pose (Green) after docking (A) 4M0E–1YL (acetylcholinesterase), RMSD = 0.18 Å; (B) 6QAA–HUN (butyrylcholinesterase), RMSD = 1.96 Å; (C) 5NN8–Acarbose (lysosomal acid α glucosidase), RMSD = 1.45 Å; (D) 4GQR–MYC (pancreatic α amylase), RMSD = 2.08 Å.

## Abbreviations

The following abbreviations are used in this manuscript:

AD	Alzheimer’s disease
T2DM	Type 2 Diabetes Mellitus
AGEs	Advanced glycation end products
AChE	Acetylcholinesterase
BChE	Butyrylcholinesterase
ROS	Reactive oxygen species
MTDL	Multi-target-directed ligand
CAS	Catalytic active site
PAS	Peripheral anionic site
SAR	Structure-activity relationship
TLC	Thin-layer chromatography
MD	Molecular dynamics
DPPH	2,2-Diphenyl-1-picrylhydrazyl
ABTS	2,2′-Azino-bis(3-ethylbenzothiazoline-6-sulfonic acid)
CUPRAC	Cupric reducing antioxidant capacity
