# Supplementary material for: Synthesis, Characterization, Biological Evaluation and In Silico Studies of Some New Thiazole Derivatives
Source: Pharmaceuticals (Basel). 2026 Sep 16;19(9):1468. doi: 10.3390/ph19091468 (PMC13609992; doi:10.3390/ph19091468)
Supplement: Supplementary file 1 [file pharmaceuticals-19-01468-s001.zip › pharmaceuticals-4510978-supplementary.pdf]

# Synthesis, characterization, biological evaluation and in silico studies of some new thiazole derivatives

**Tuğçe Çaklılı [1,2], Yusuf Sıcak [3], Somdatta Y. Chaudhari [4], Suraj N. Mali [5], Dilaycan Çam [6], Gülin Gümüşbulut Şener [6], Mehmet Öztürk [6,7], Mert Ülgen [1] and Fatih Tok [8,*]**

1 Department of Pharmaceutical Chemistry, Faculty of Pharmacy, Acıbadem Mehmet Ali Aydınlar University, İstanbul 34752, Türkiye; tugce.caklili@acibadem.edu.tr (T.Ç.); Mert.Ulgen@acibadem.edu.tr (M.Ü.)
2 Institute of Health Sciences, Marmara University, İstanbul 34865, Türkiye
3 Department of Medicinal and Aromatic Plants, Köyceğiz Vocational School, Mugla Sitki Kocman University, Muğla 48800, Türkiye; ysicak@gmail.com
4 Department of Pharmaceutical Chemistry, PES's Modern College of Pharmacy, Pune 411044, Maharashtra, India; somu.chaudhari@gmail.com
5 School of Pharmacy, D. Y. Patil University (Deemed to Be University), Navi Mumbai 400706, Maharashtra, India; suraj.mali@dypatil.edu
6 Department of Chemistry, Faculty of Sciences, Mugla Sitki Kocman University, Muğla 48000, Türkiye; dilaycancam@gmail.com (D.Ç.); gulingumusbulut@posta.mu.edu.tr (G.G.Ş.); mehmetozturk@mu.edu.tr (M.Ö.)
7 Faculty of Chemistry and Chemical Technology, Farabi University, Almaty 050040, Kazakhstan
8 Department of Pharmaceutical Chemistry, Faculty of Pharmacy, Marmara University, İstanbul 34854, Türkiye
* Correspondence: fatih.tok@marmara.edu.tr

## LIST OF CONTENTS

**Figure S1**. IR spectrum for **1**
**Figure S2**. $^{1}$H-NMR spectrum for **1**
**Figure S3**. $^{13}$C-NMR spectrum for **1**
**Figure S4**. LC-MS spectrum for **1**
**Figure S5**. LC-HRMS spectrum for **1**
**Figure S6**. IR spectrum for **2**
**Figure S7**. $^{1}$H-NMR spectrum for **2**
**Figure S8**. $^{13}$C-NMR spectrum for **2**
**Figure S9**. LC-MS spectrum for **2**
**Figure S10**. LC-HRMS spectrum for **2**
**Figure S11**. IR spectrum for **3**
**Figure S12**. $^{1}$H-NMR spectrum for **3**
**Figure S13**. $^{13}$C-NMR spectrum for **3**
**Figure S14**. LC-MS spectrum for **3**
**Figure S15**. LC-HRMS spectrum for **3**
**Figure S16**. IR spectrum for **4**
**Figure S17**. $^{1}$H-NMR spectrum for **4**
**Figure S18**. $^{13}$C-NMR spectrum for **4**
**Figure S19**. LC-MS spectrum for **4**
**Figure S20**. LC-HRMS spectrum for **4**
**Figure S21**. IR spectrum for **5**
**Figure S22**. $^{1}$H-NMR spectrum for **5**
**Figure S23**. $^{13}$C-NMR spectrum for **5**
**Figure S24**. LC-MS spectrum for **5**
**Figure S25**. LC-HRMS spectrum for **5**
**Figure S26**. IR spectrum for **6**
**Figure S27**. $^{1}$H-NMR spectrum for **6**
**Figure S28**. $^{13}$C-NMR spectrum for **6**

**Figure S29**. LC-MS spectrum for **6**
**Figure S30**. LC-HRMS spectrum for **6**
**Figure S31**. IR spectrum for **7**
**Figure S32**. $^{1}$H-NMR spectrum for **7**
**Figure S33**. $^{13}$C-NMR spectrum for **7**
**Figure S34**. LC-MS spectrum for **7**
**Figure S35**. LC-HRMS spectrum for **7**
**Figure S36**. IR spectrum for **8**
**Figure S37**. $^{1}$H-NMR spectrum for **8**
**Figure S38**. $^{13}$C-NMR spectrum for **8**
**Figure S39**. LC-MS spectrum for **8**
**Figure S40**. LC-HRMS spectrum for **8**
**Figure S41**. IR spectrum for **9**
**Figure S42**. $^{1}$H-NMR spectrum for **9**
**Figure S43**. $^{13}$C-NMR spectrum for **9**
**Figure S44**. LC-MS spectrum for **9**
**Figure S45**. LC-HRMS spectrum for **9**
**Figure S46**. HSQC spectrum for **3**
**Figure S47**. 2D and 3D interactions between ligands and protein 5D41
**Figure S48**. Validation of docking protocol through redocking of cocrystal ligand in binding pocket of respective targets. Superimposition of experimental co-crystal ligand pose (Orange) before docking and re-docked pose (Green) after docking **(A)** 4M0E–1YL (acetylcholinesterase), RMSD = 0.18 Å; **(B)** 6QAA–HUN (butyrylcholinesterase), RMSD = 1.96 Å; **(C)** 5NN8–Acarbose (lysosomal acid α-glucosidase), RMSD = 1.45 Å; **(D)** 4GQR–MYC (pancreatic α-amylase), RMSD = 2.08 Å.

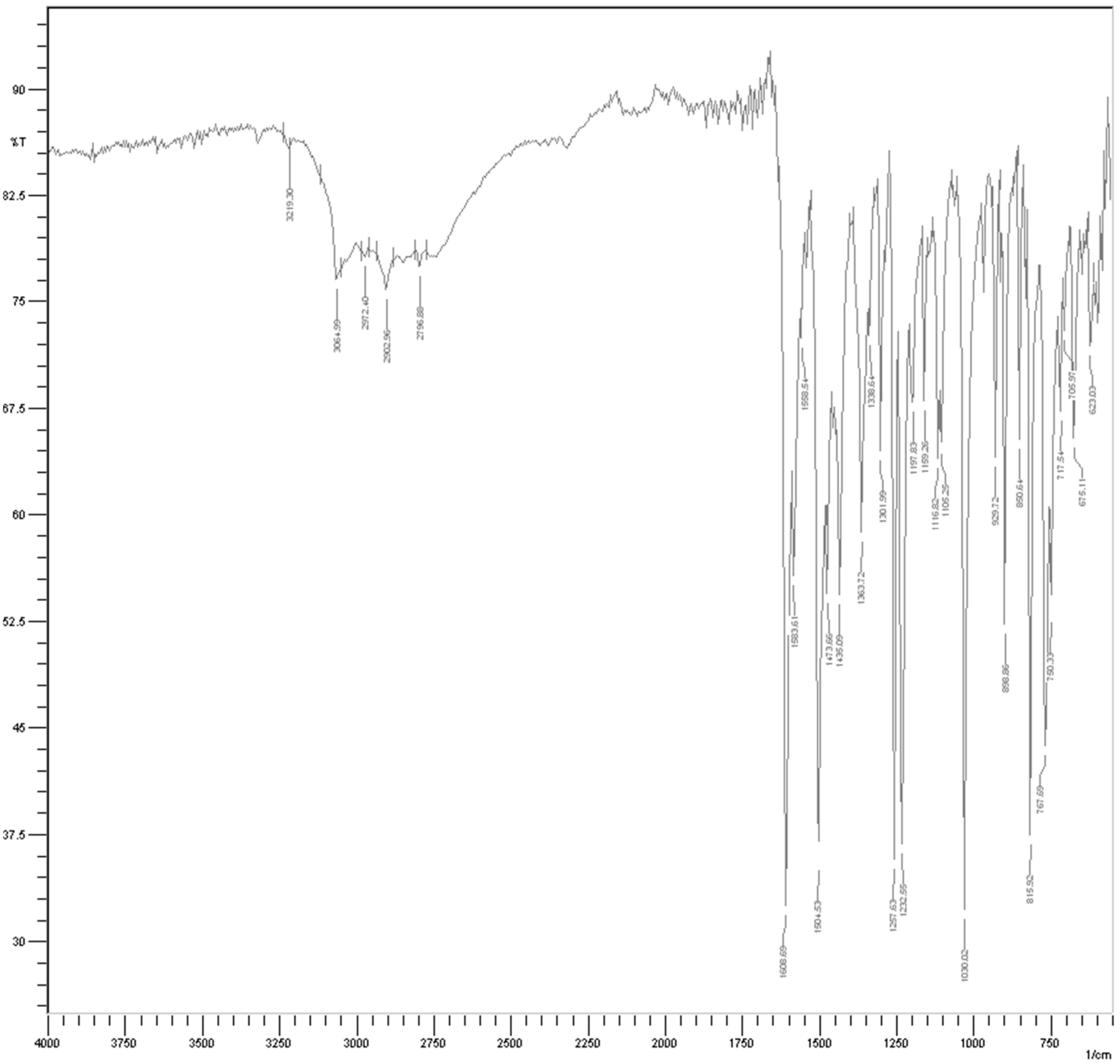


**Figure S1**. IR spectrum for **1**

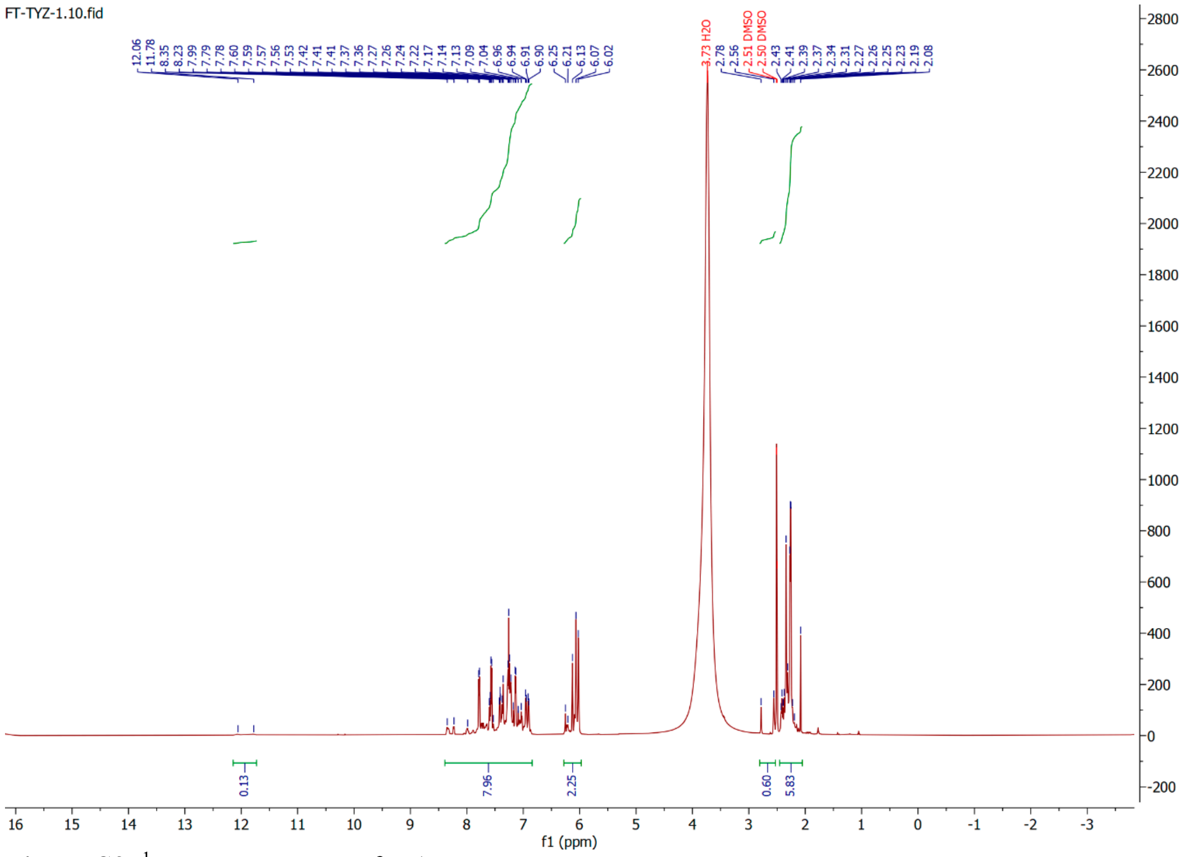


**Figure S2**. $^{1}$H-NMR spectrum for **1**

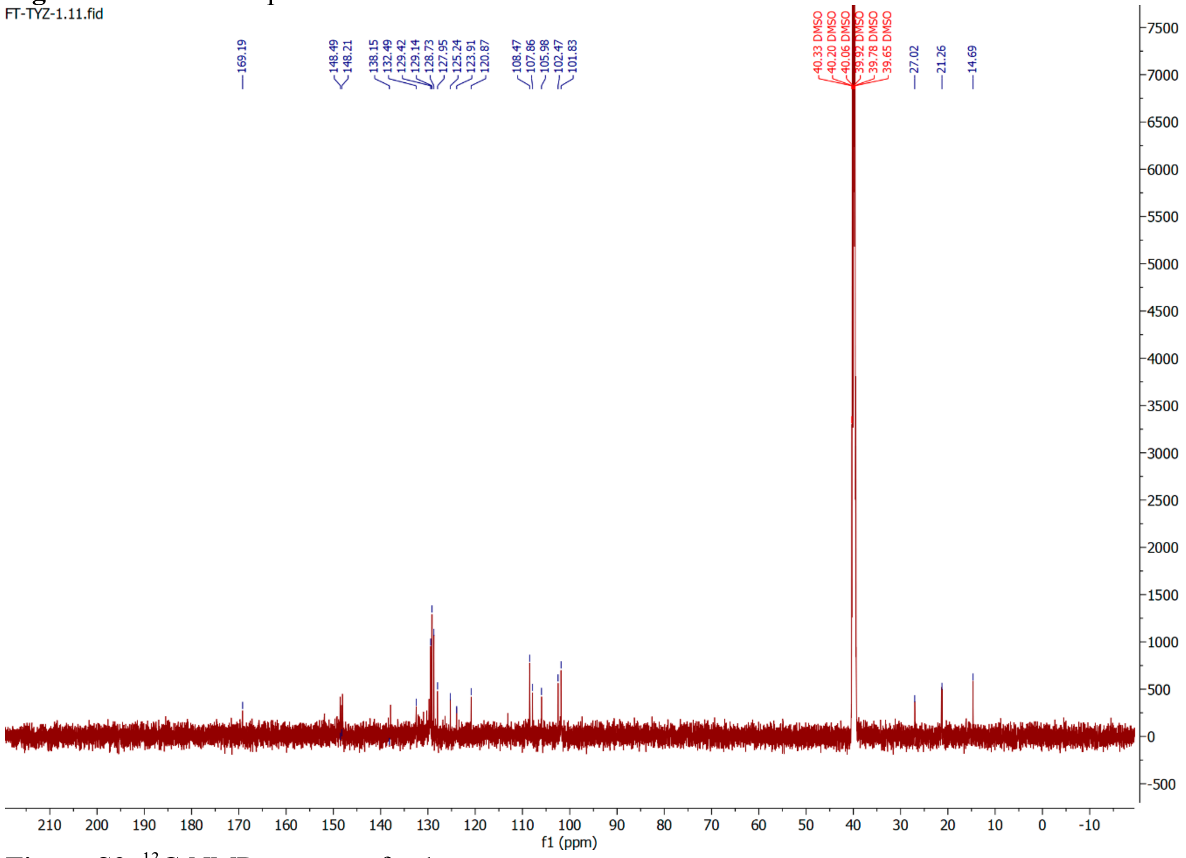


**Figure S3**. $^{13}$C-NMR spectrum for **1**

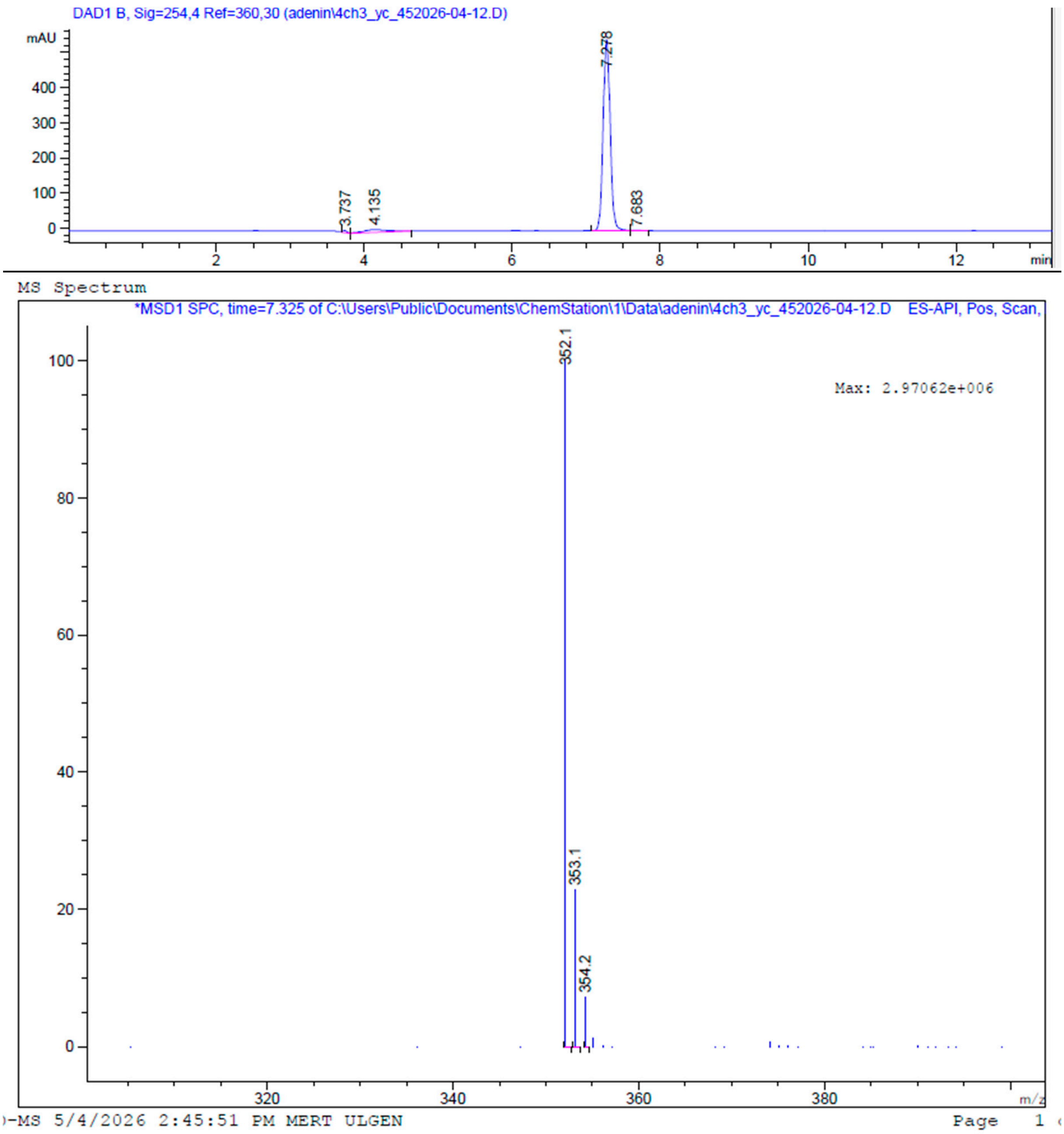


**Figure S4**. LC-MS spectrum for **1**

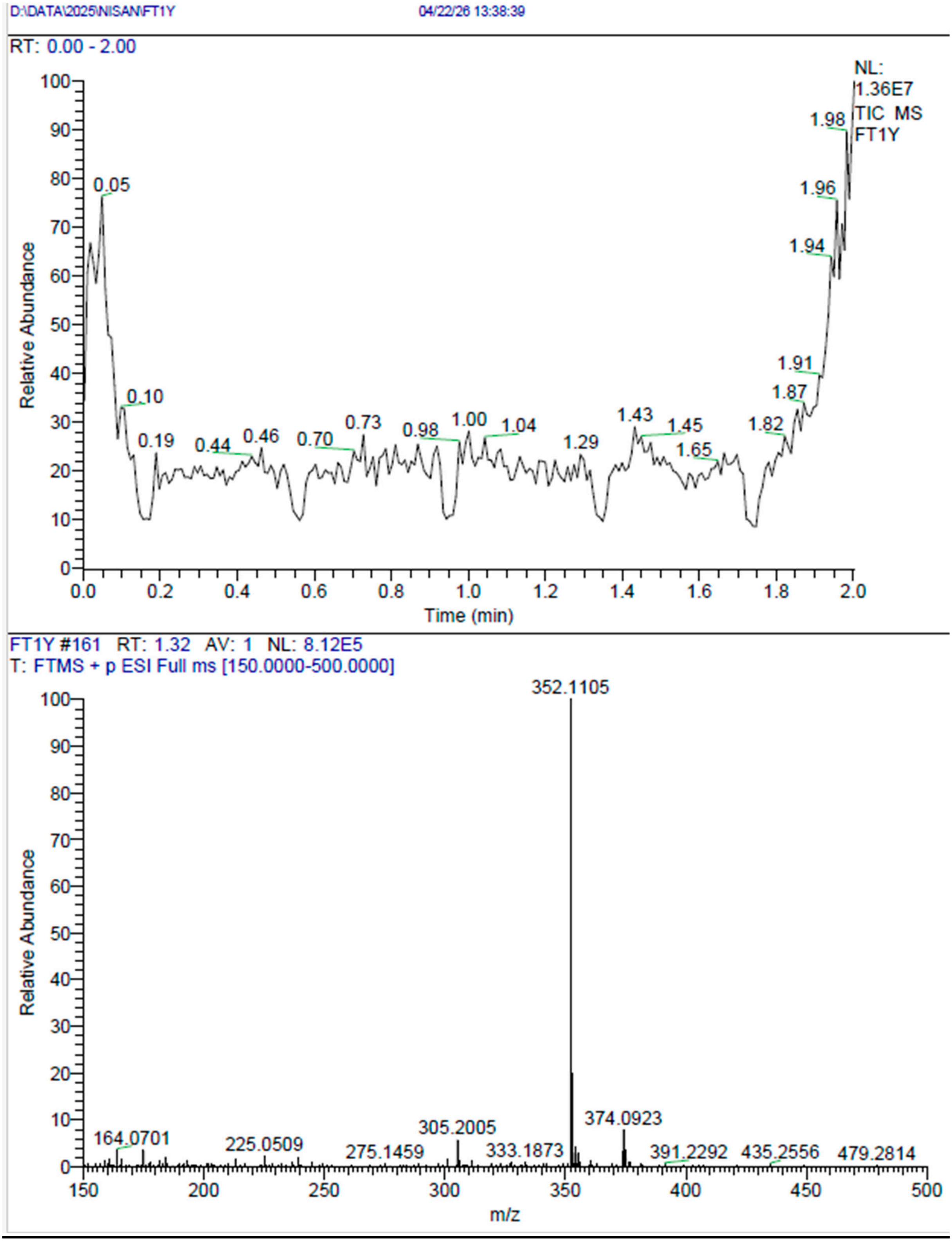


**Figure S5**. LC-HRMS spectrum for **1**

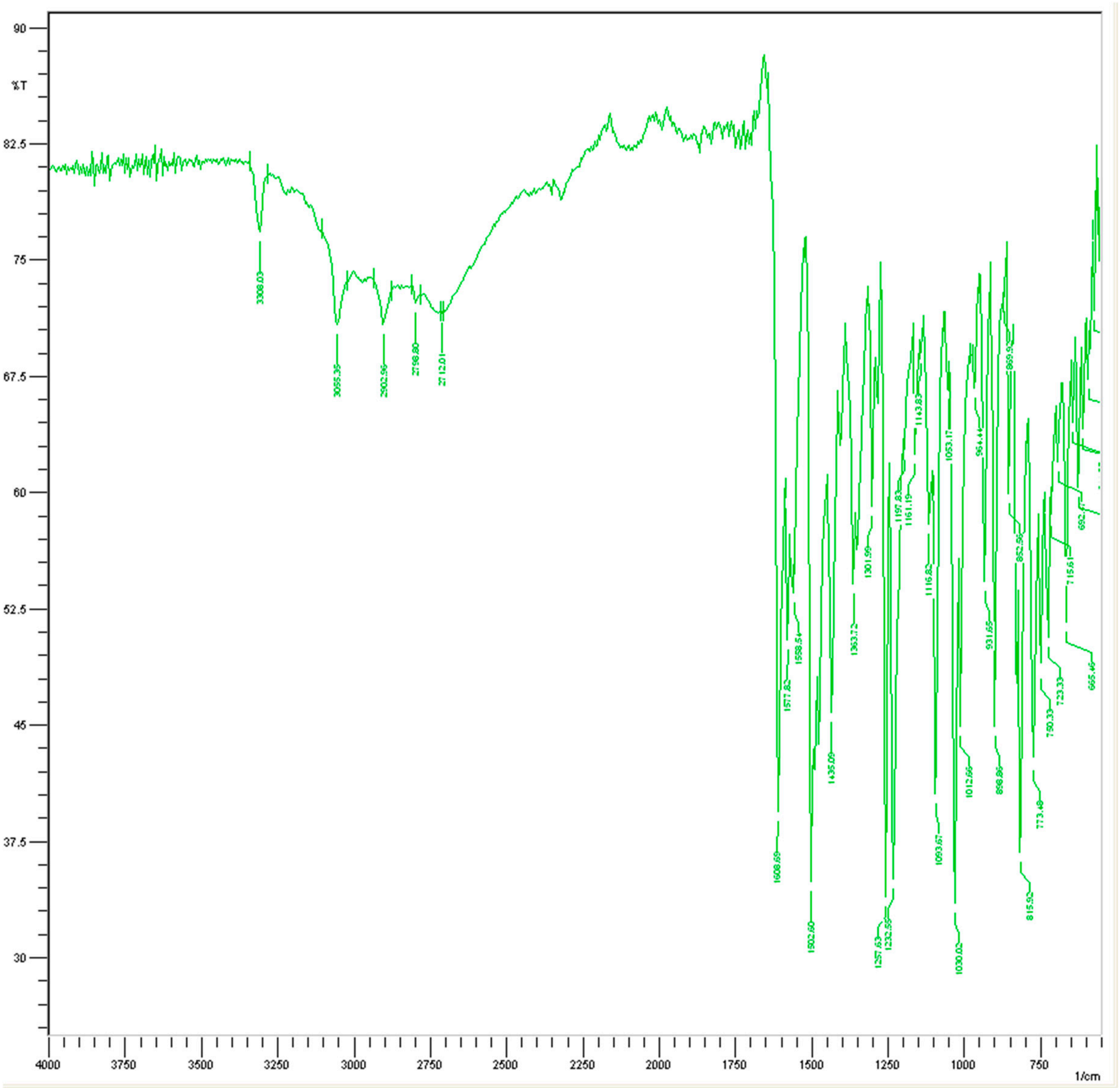


**Figure S6**. IR spectrum for **2**

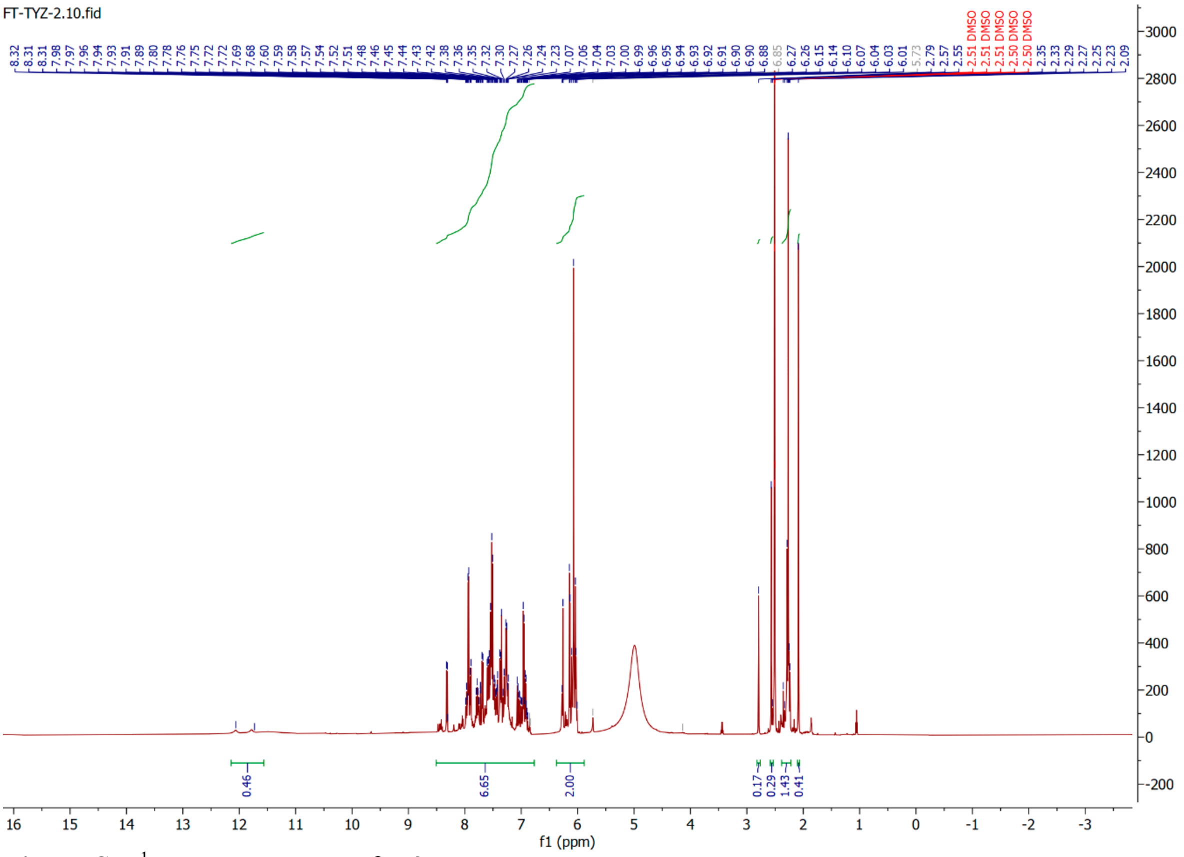


**Figure S7**. $^{1}$H-NMR spectrum for **2**

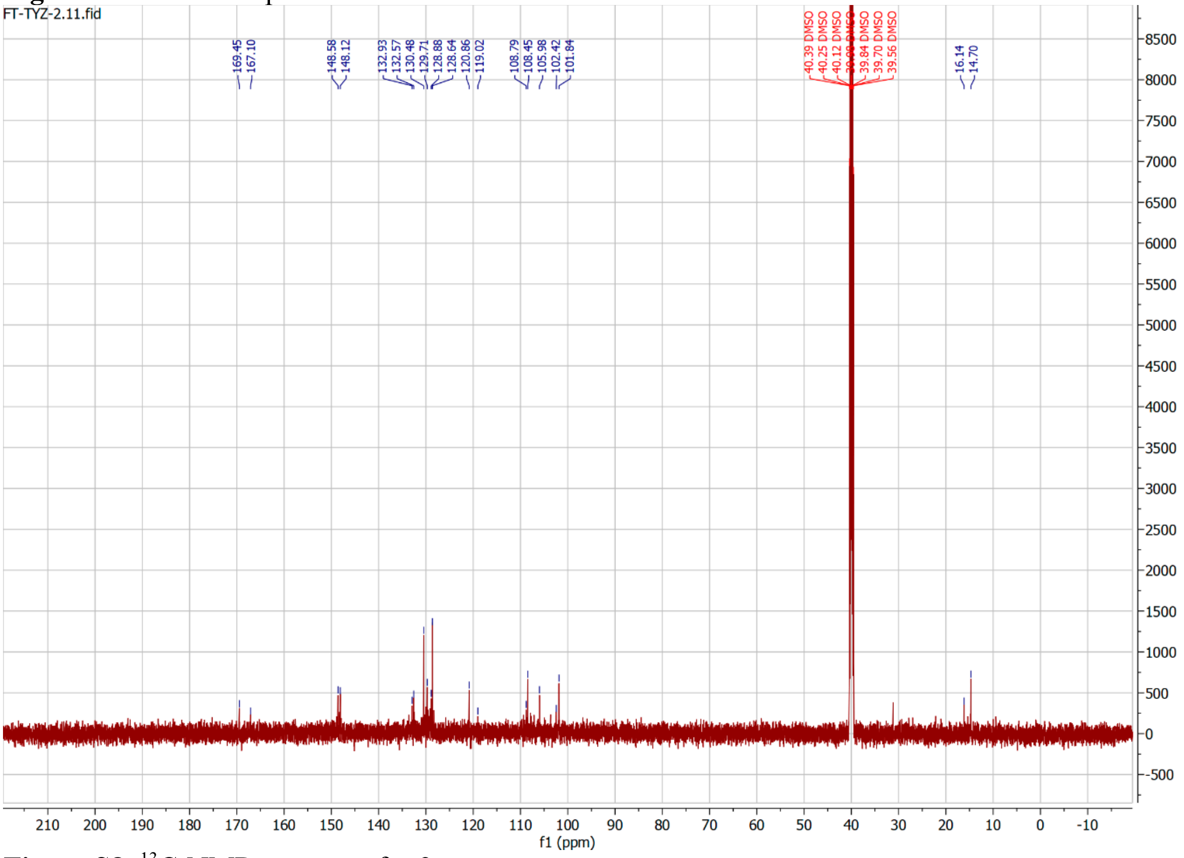


**Figure S8**. $^{13}$C-NMR spectrum for **2**

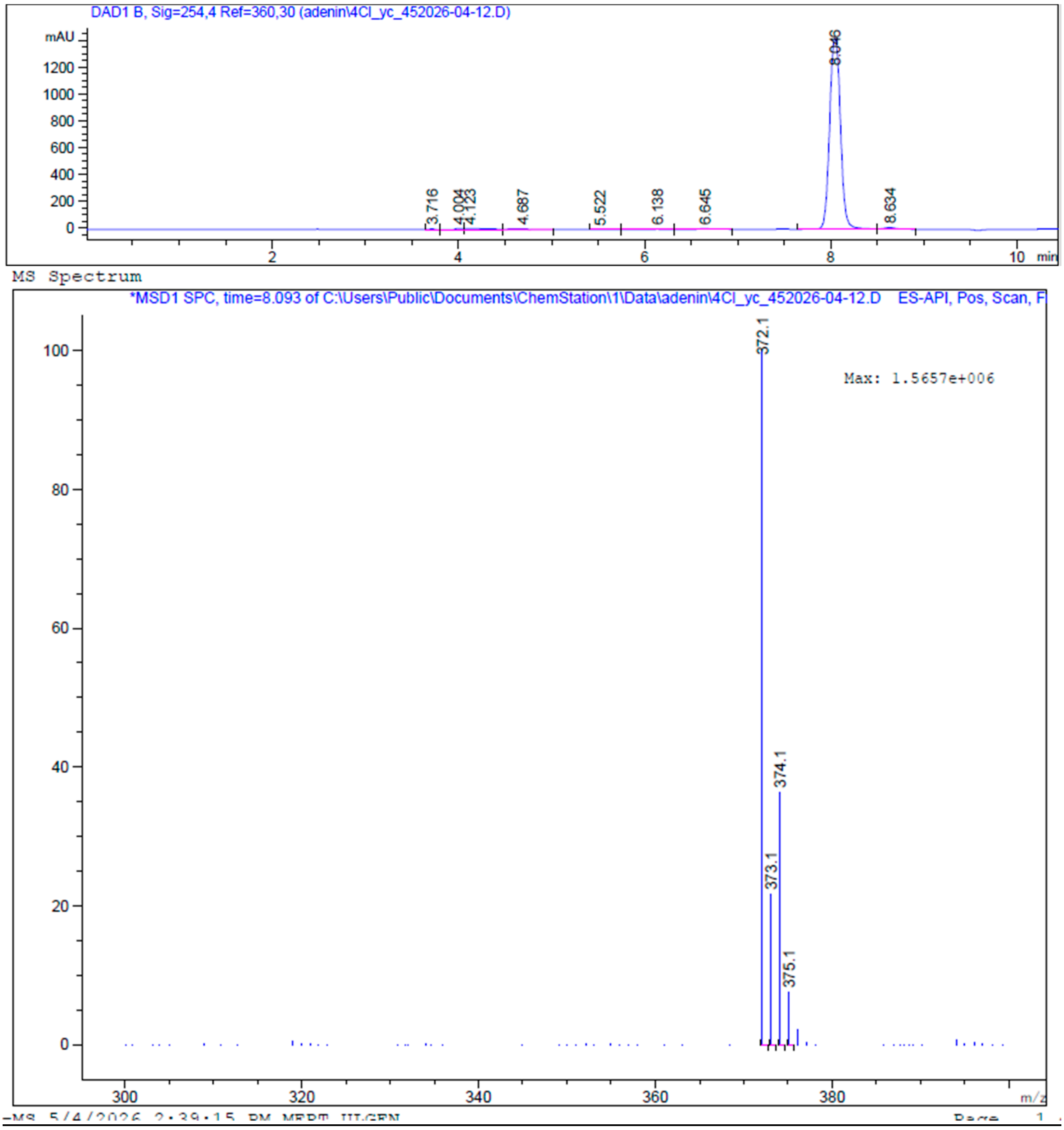


**Figure S9**. LC-MS spectrum for **2**

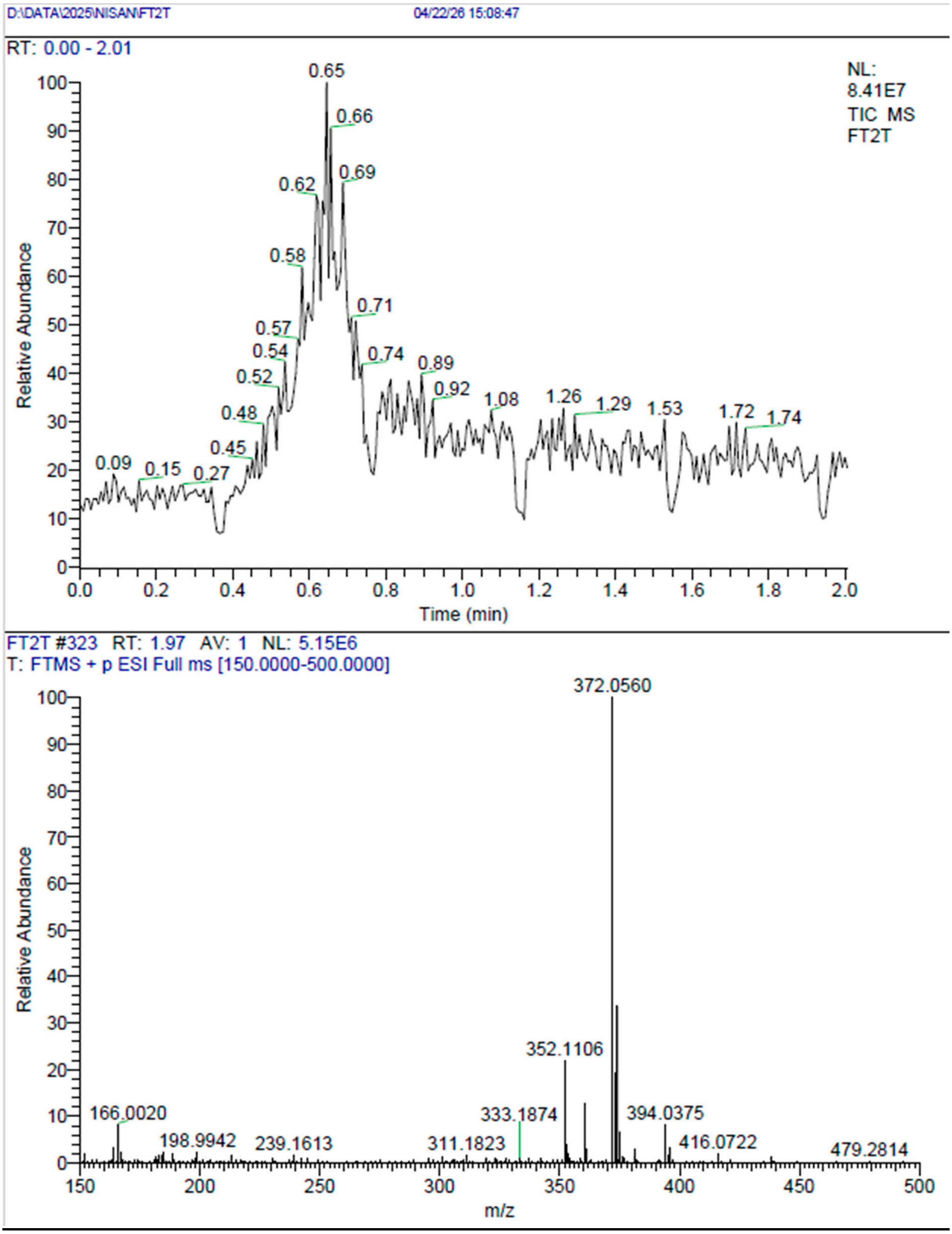


**Figure S10**. LC-HRMS spectrum for **2**

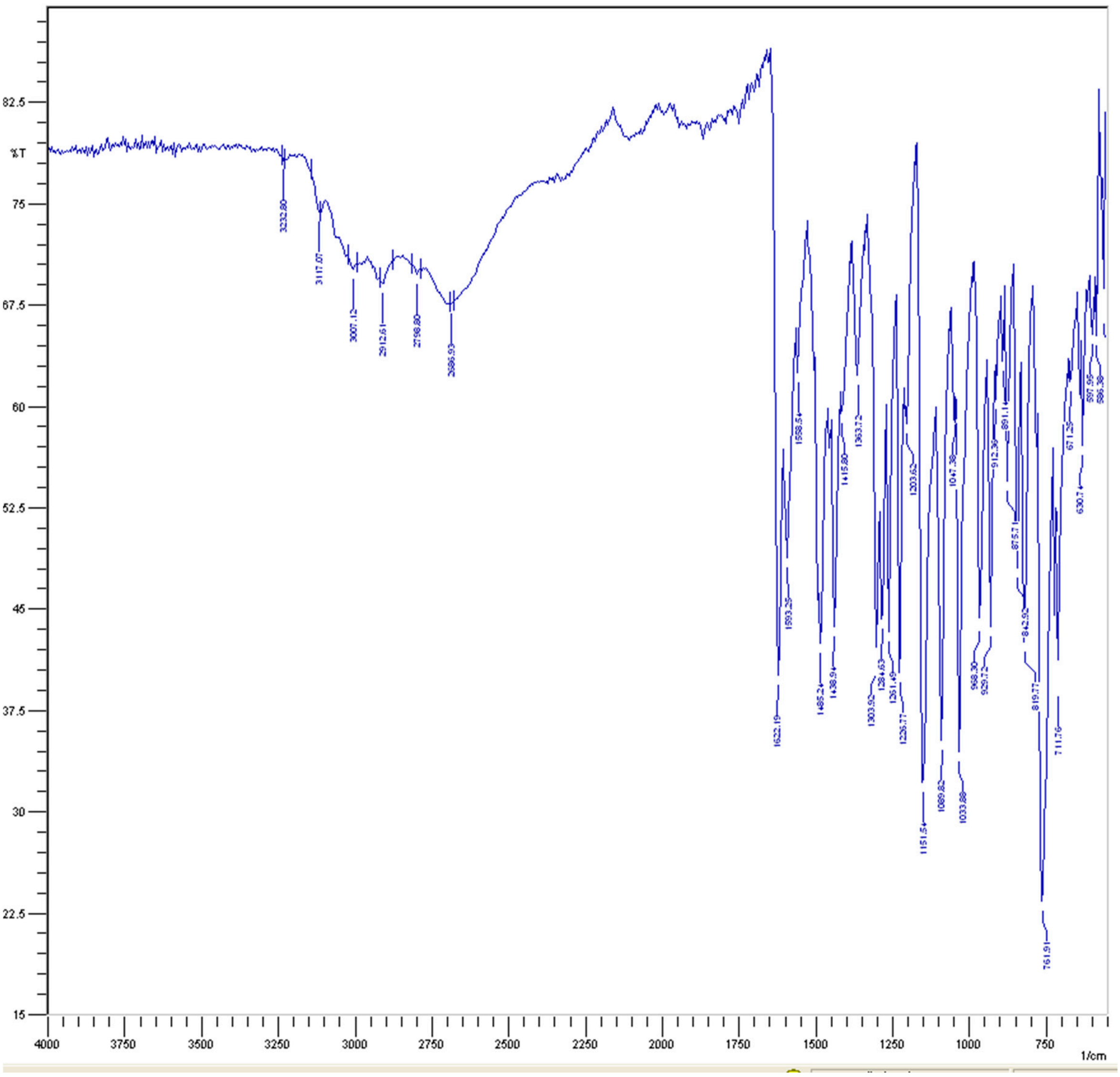


**Figure S11**. IR spectrum for **3**

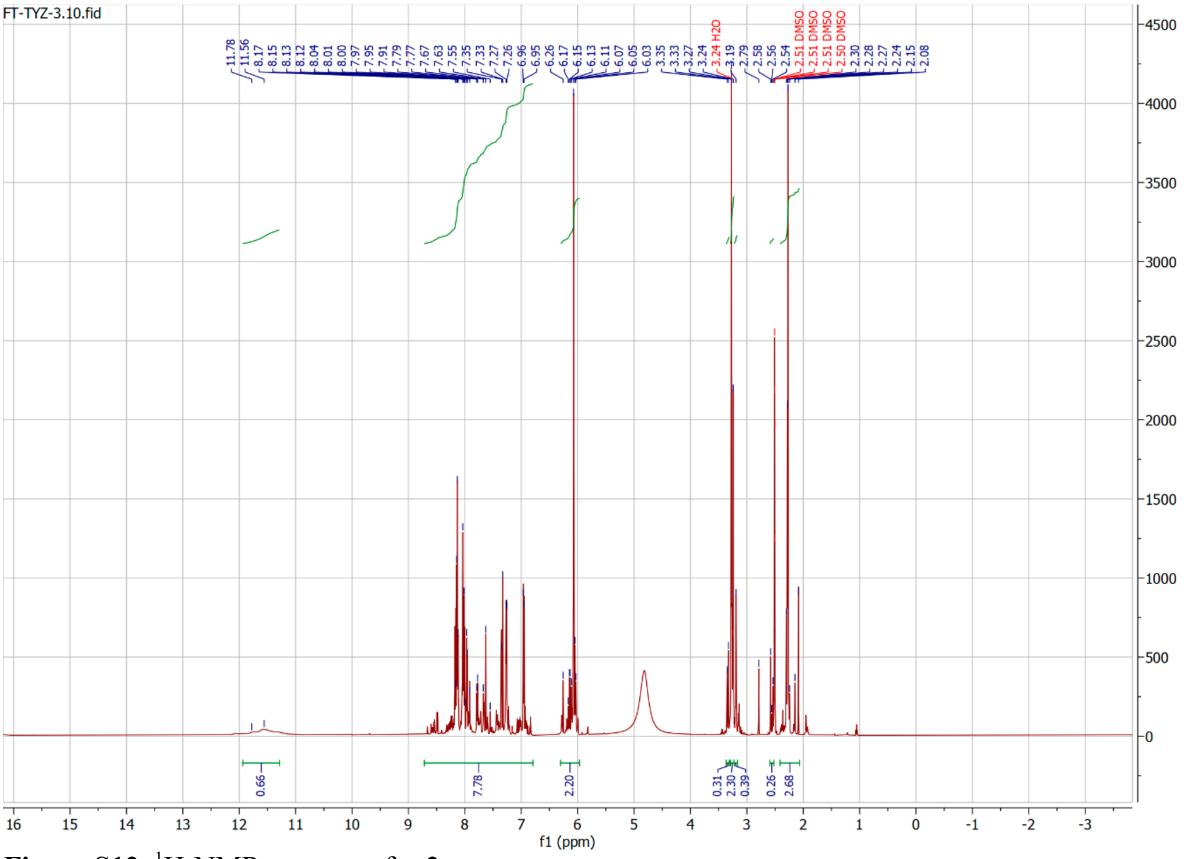

**Figure S12**. $^{1}$H-NMR spectrum for **3**

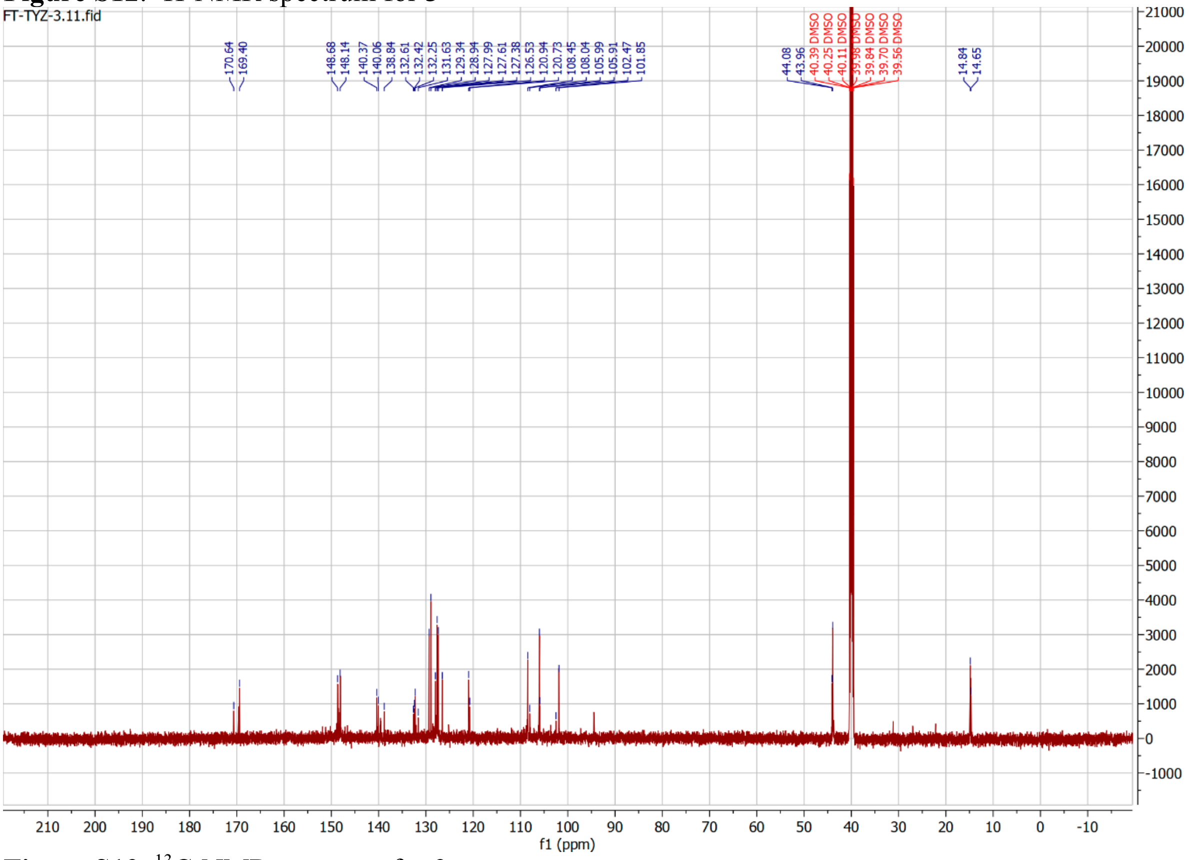

**Figure S13**. $^{13}$C-NMR spectrum for **3**

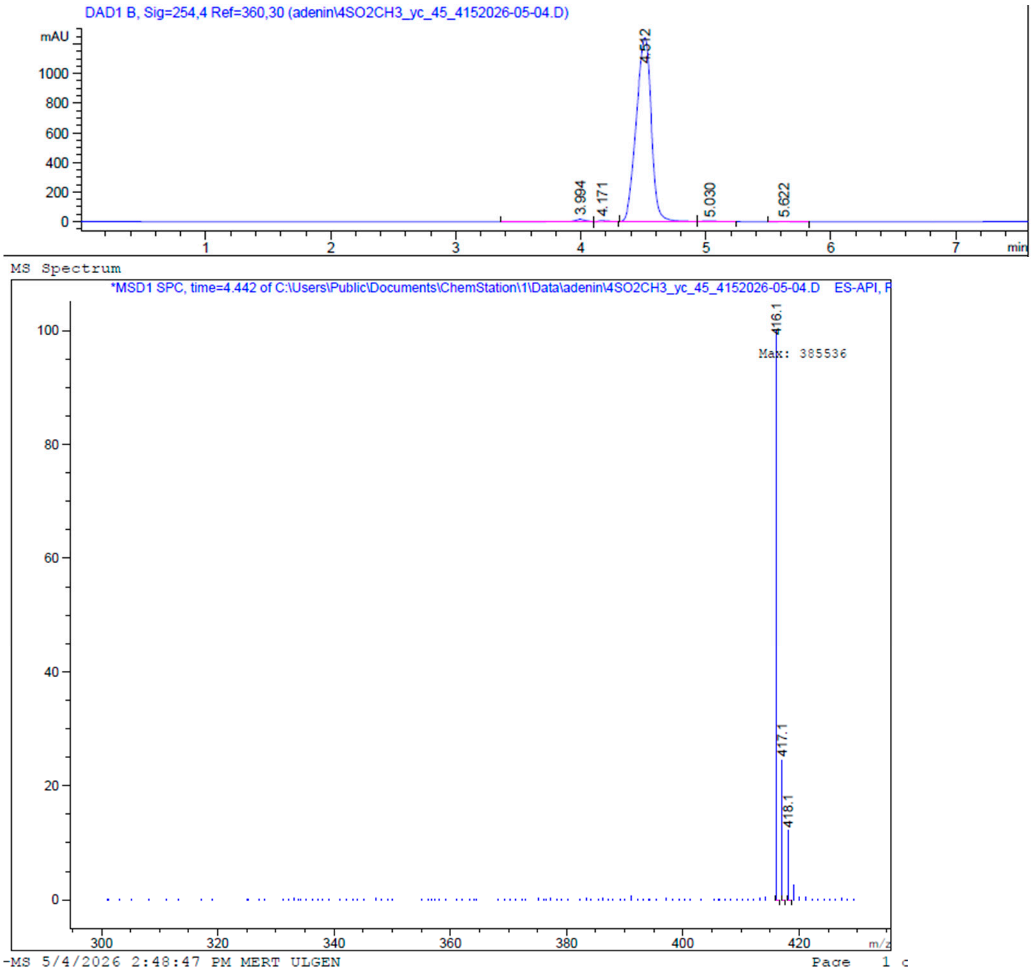


**Figure S14**. LC-MS spectrum for **3**

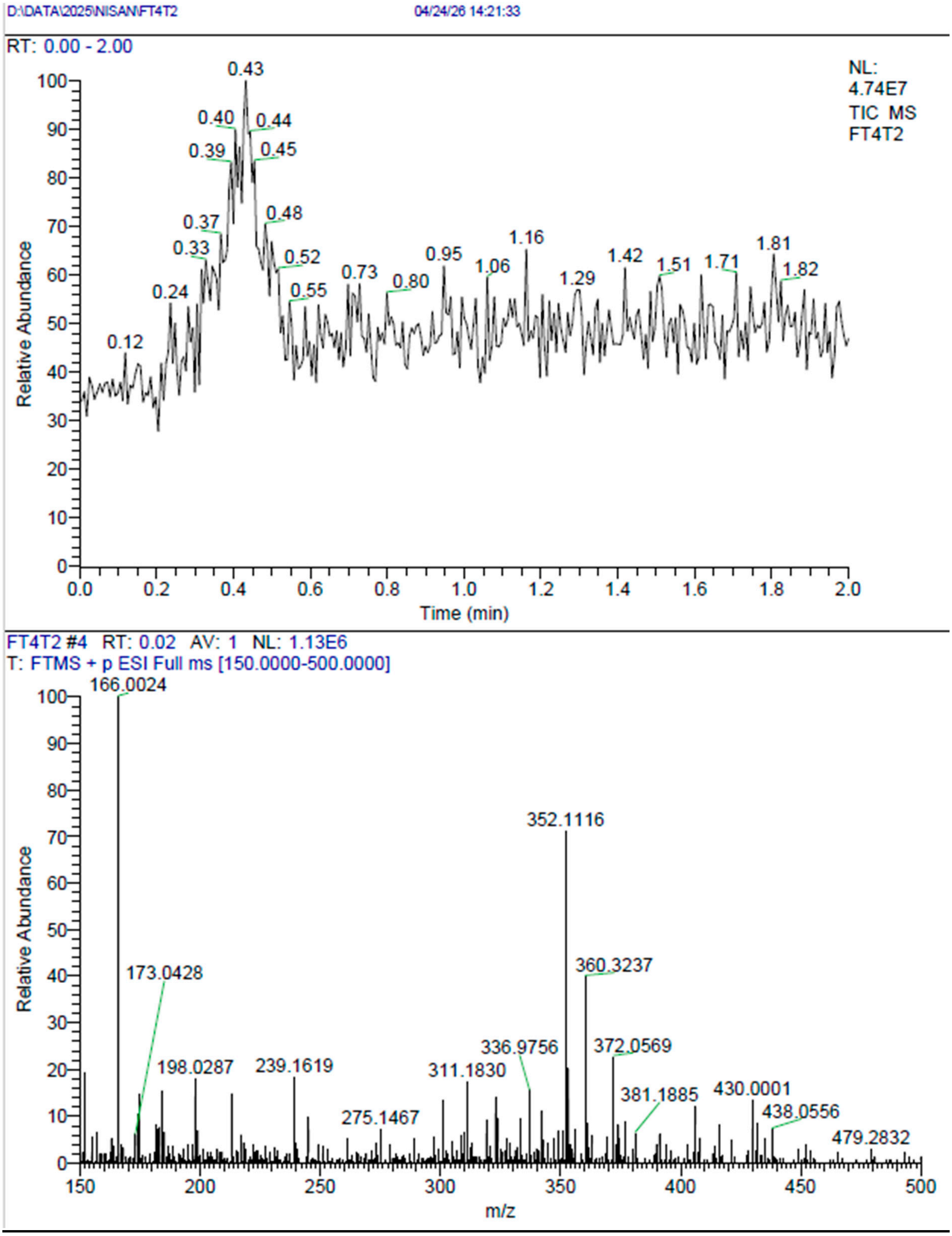


**Figure S15**. LC-HRMS spectrum for **3**

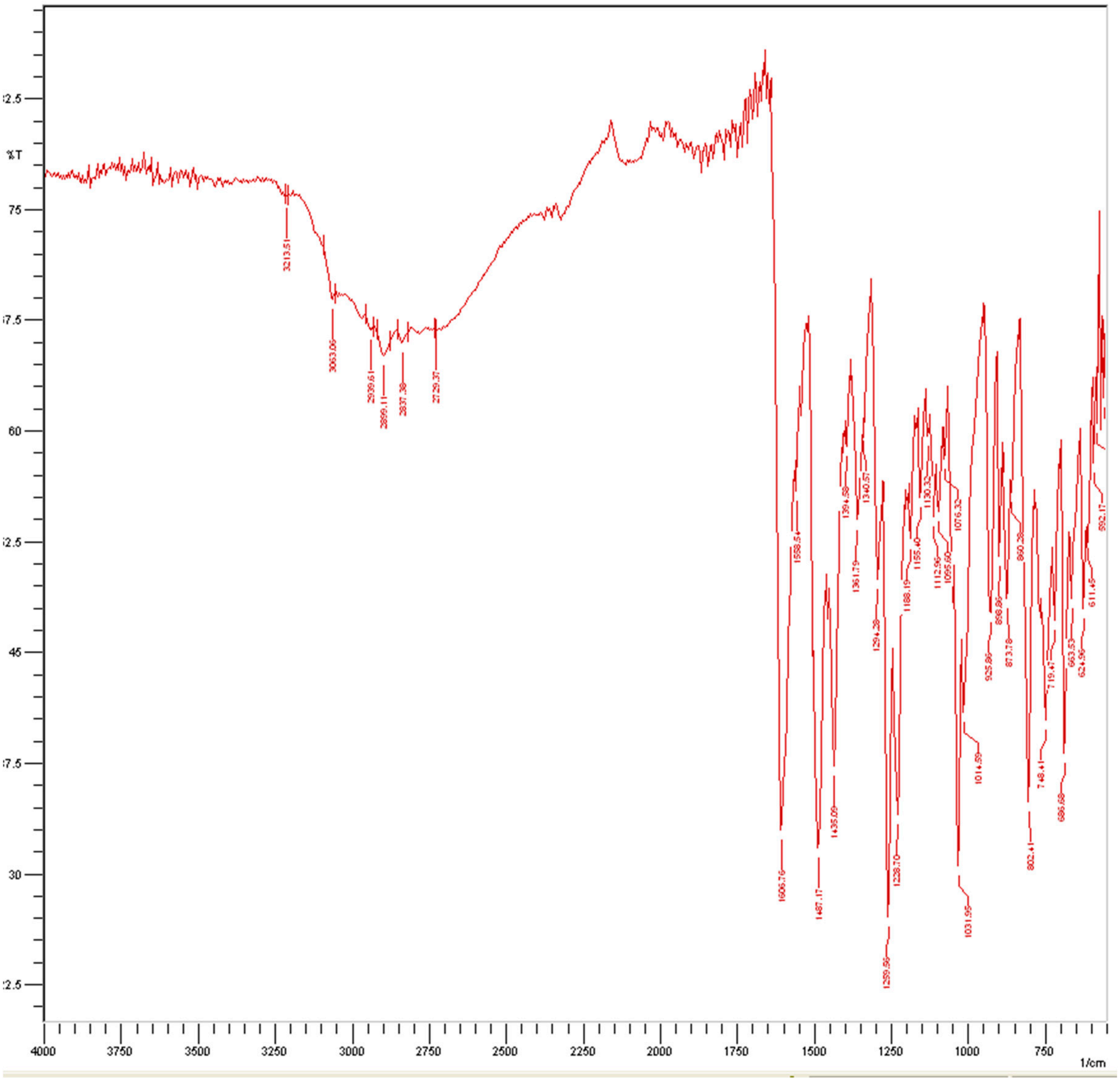


**Figure S16**. IR spectrum for **4**

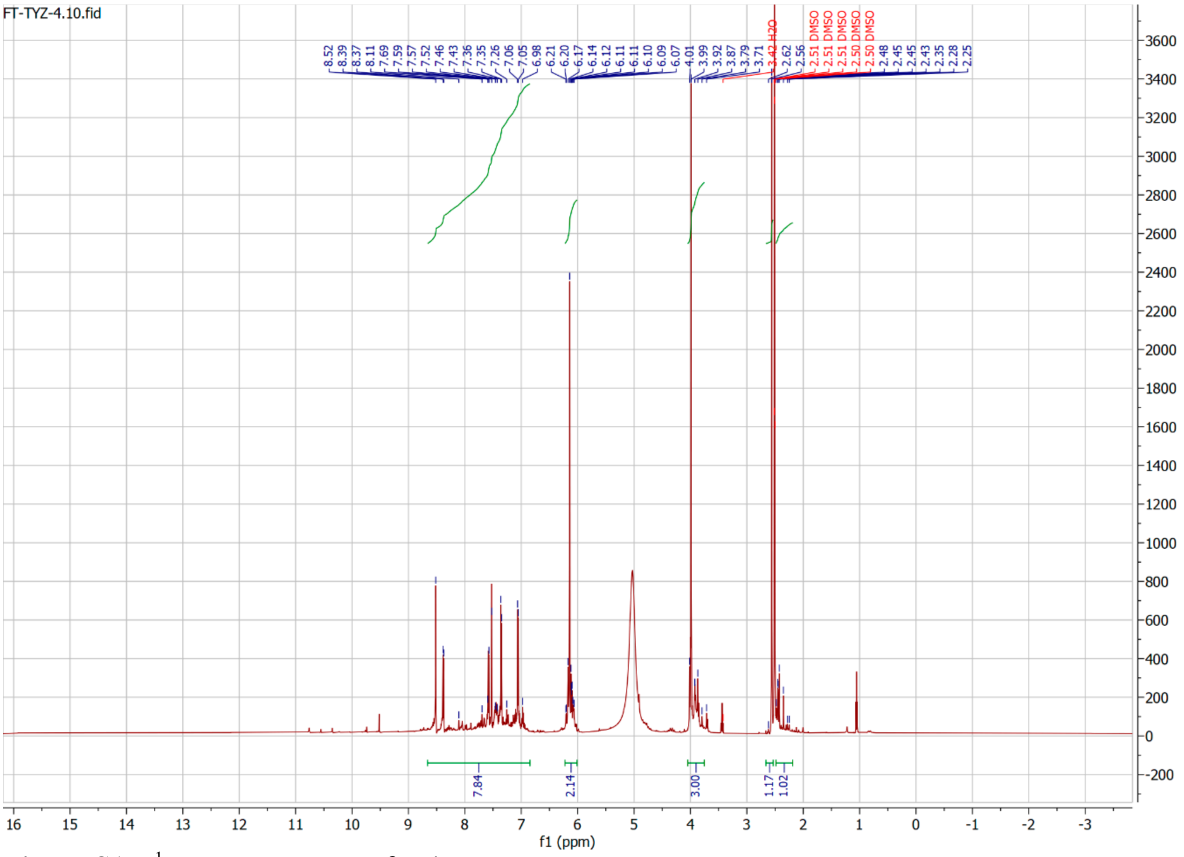


**Figure S17**. $^{1}$H-NMR spectrum for **4**

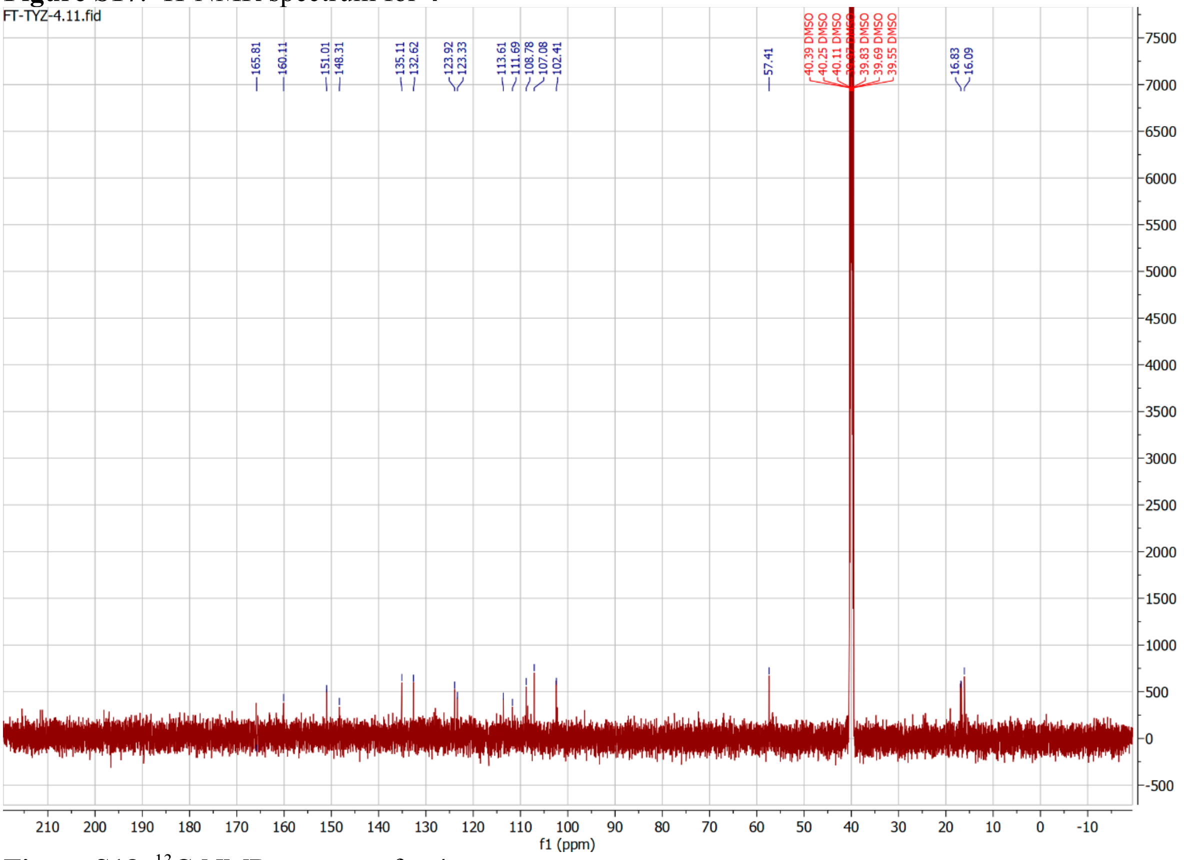


**Figure S18**. $^{13}$C-NMR spectrum for **4**

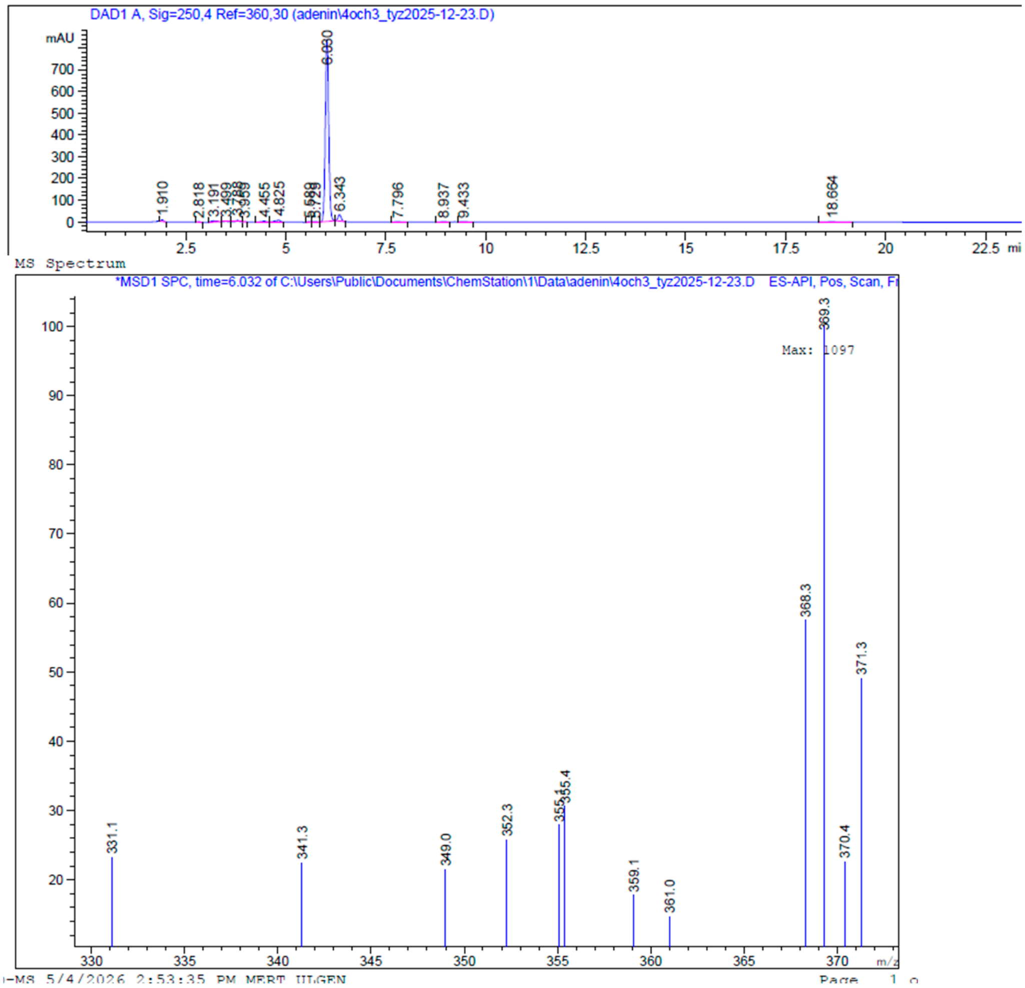


**Figure S19**. LC-MS spectrum for **4**

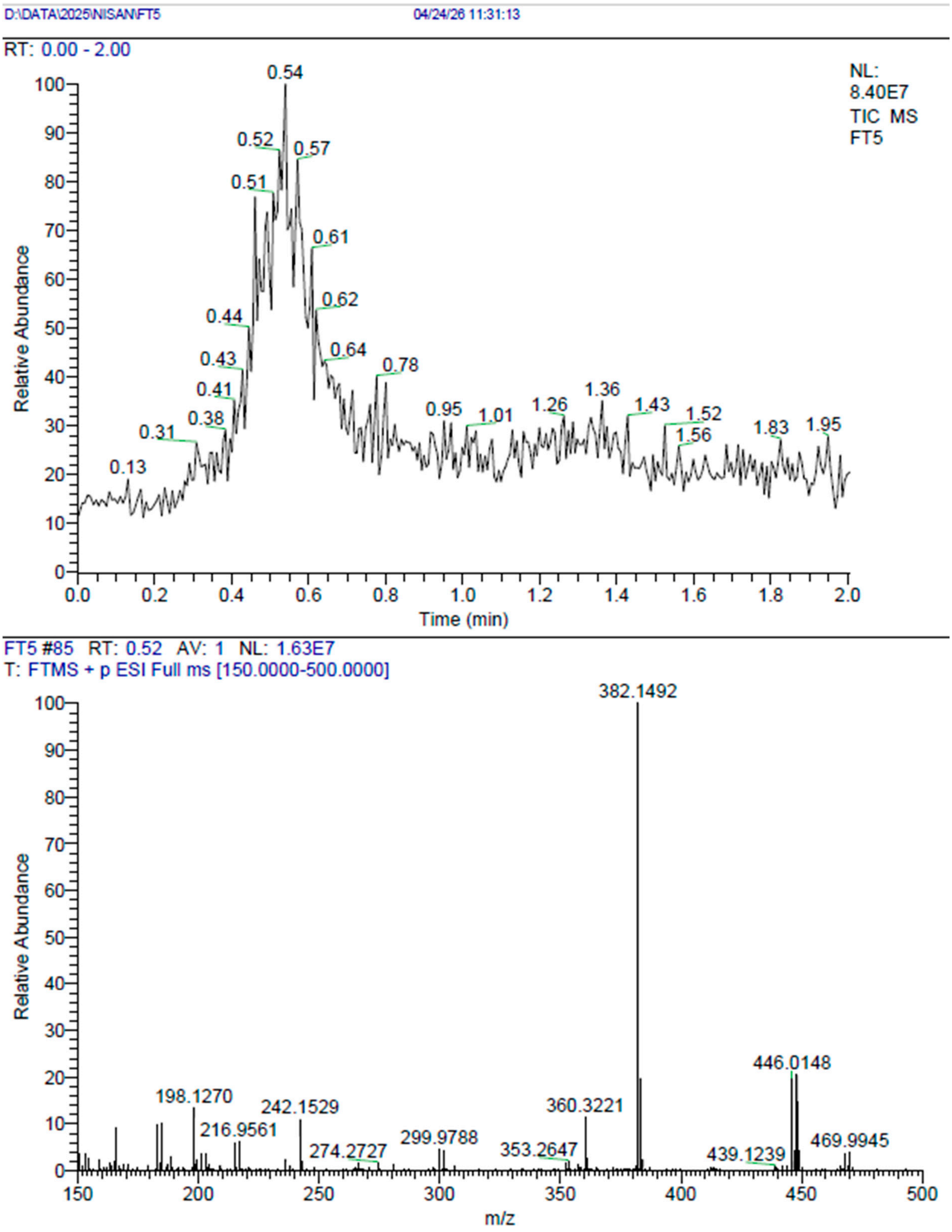


**Figure S20**. LC-HRMS spectrum for **4**

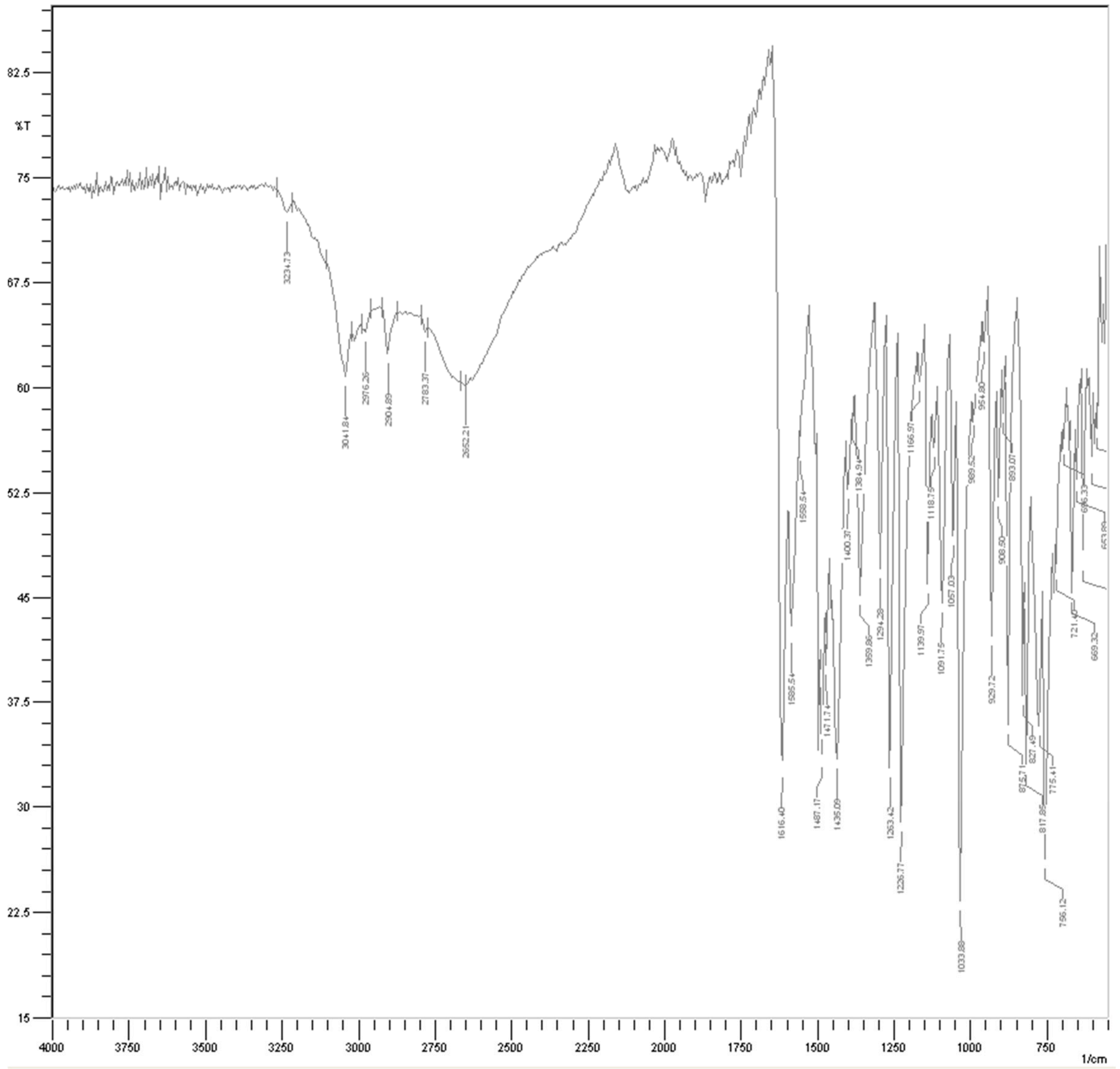

**Figure S21**. IR spectrum for **5**

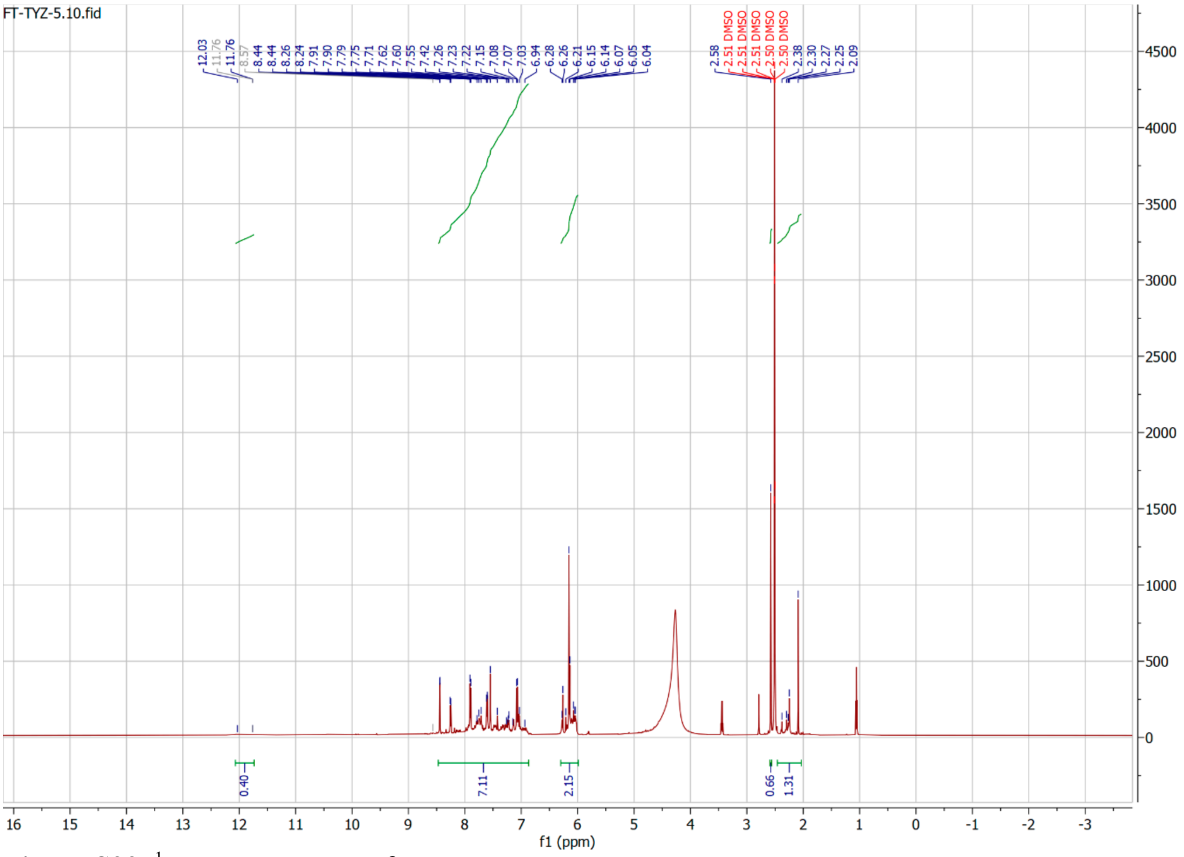


**Figure S22**. $^{1}$H-NMR spectrum for **5**

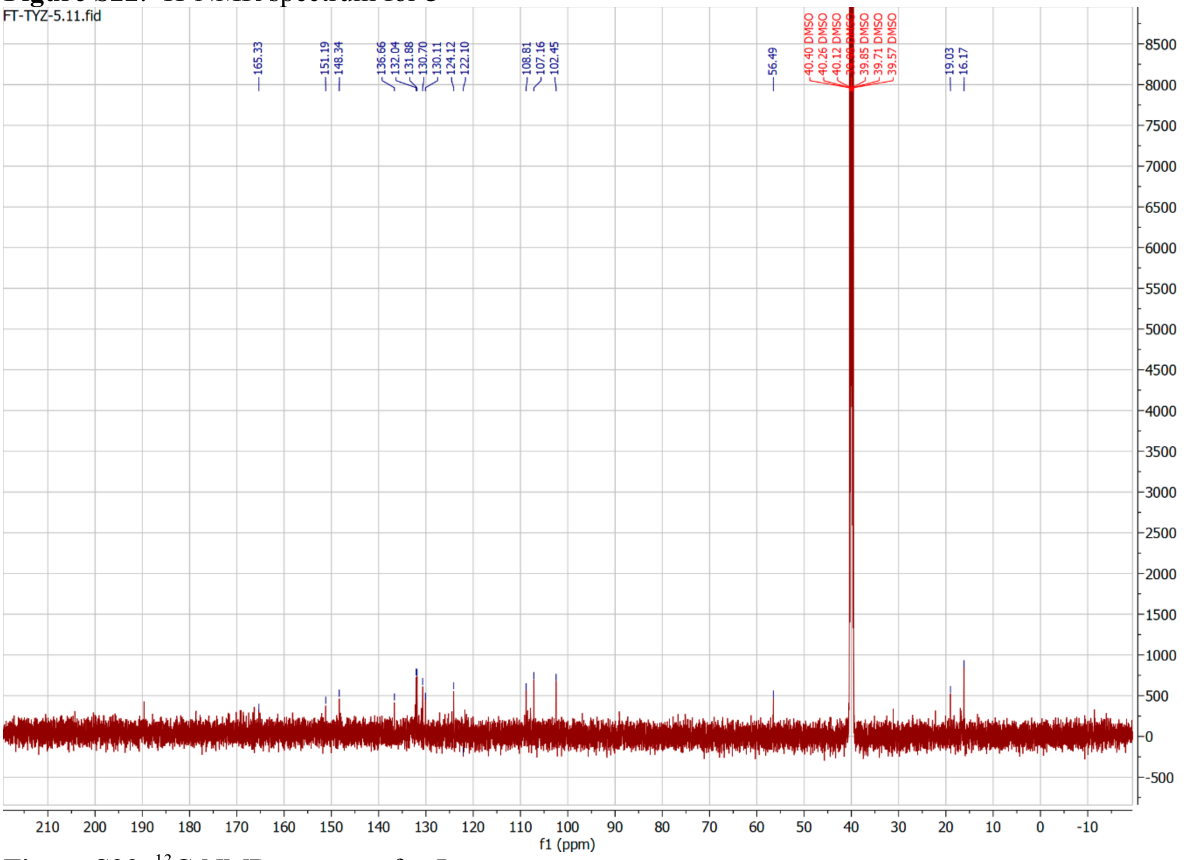


**Figure S23**. $^{13}$C-NMR spectrum for **5**

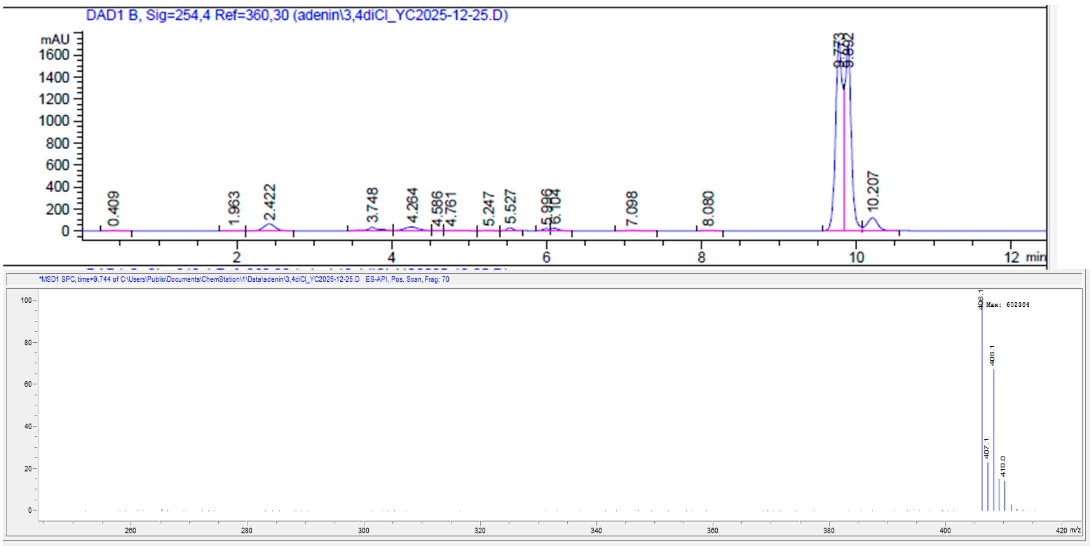


**Figure S24**. LC-MS spectrum for **5**

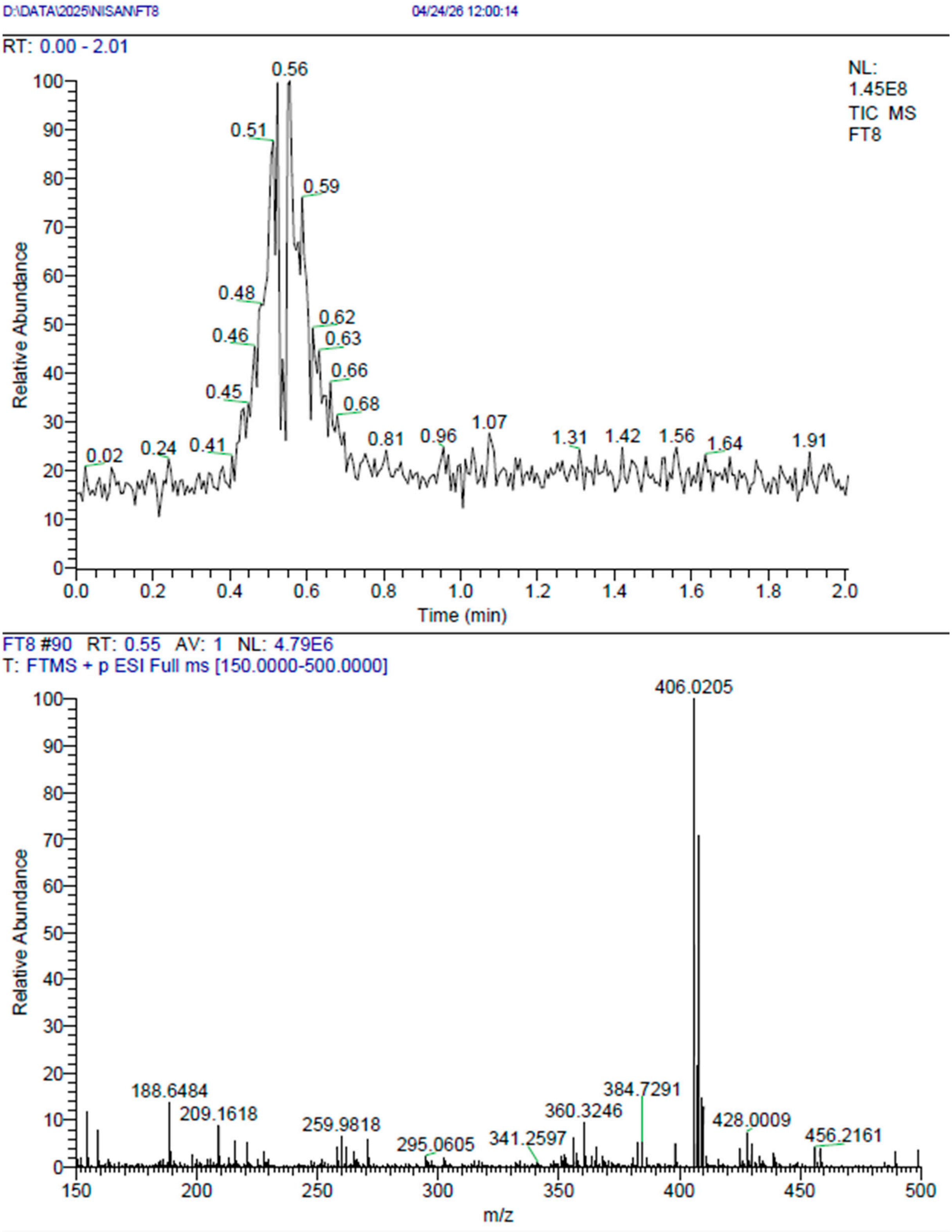


**Figure S25**. LC-HRMS spectrum for **5**

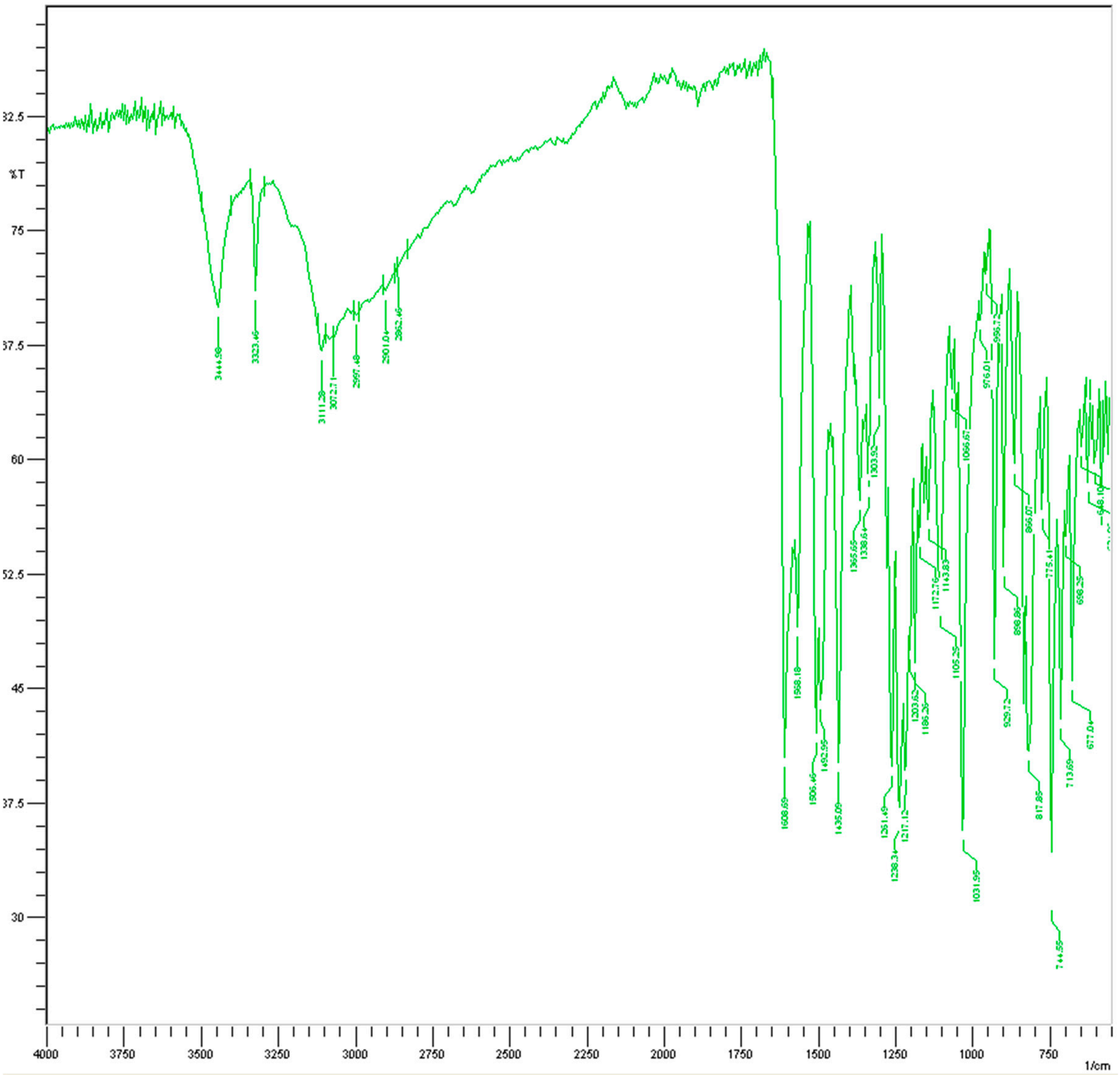

**Figure S26**. IR spectrum for **6**

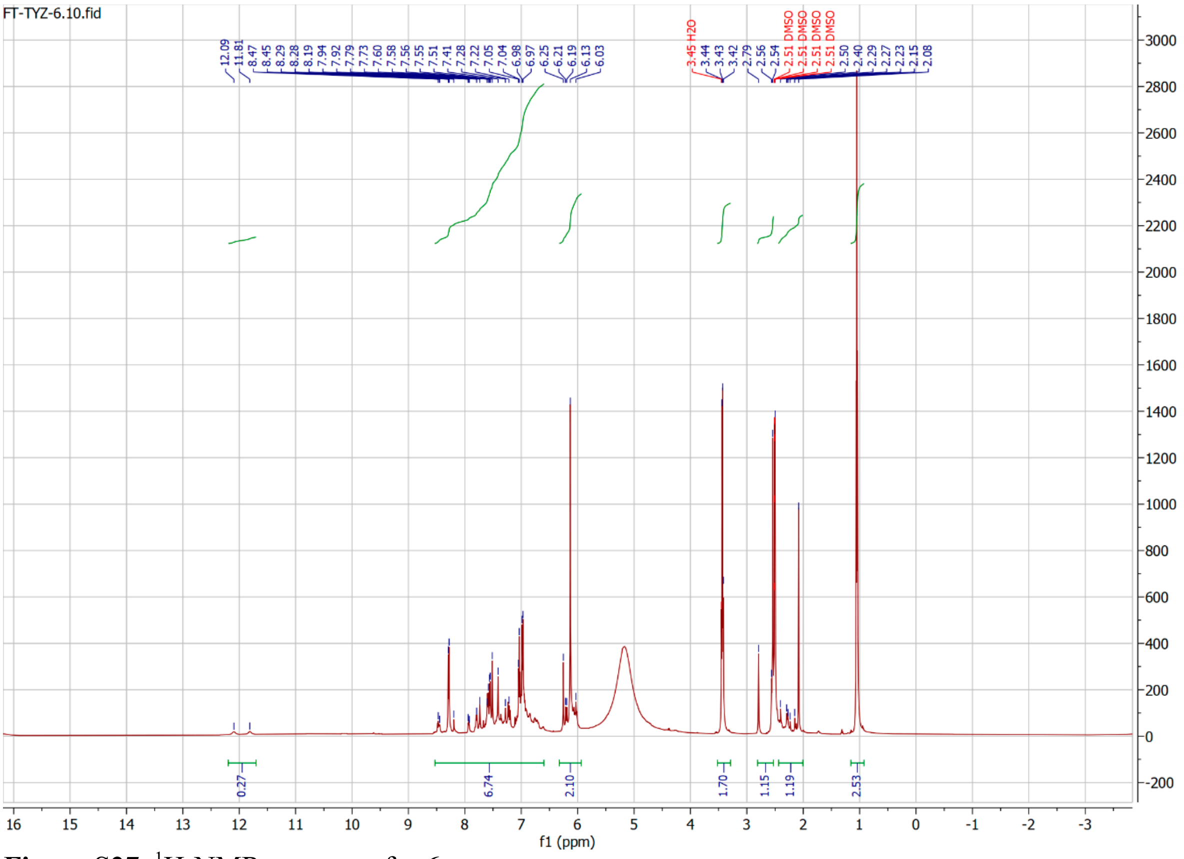


**Figure S27**. $^{1}$H-NMR spectrum for **6**

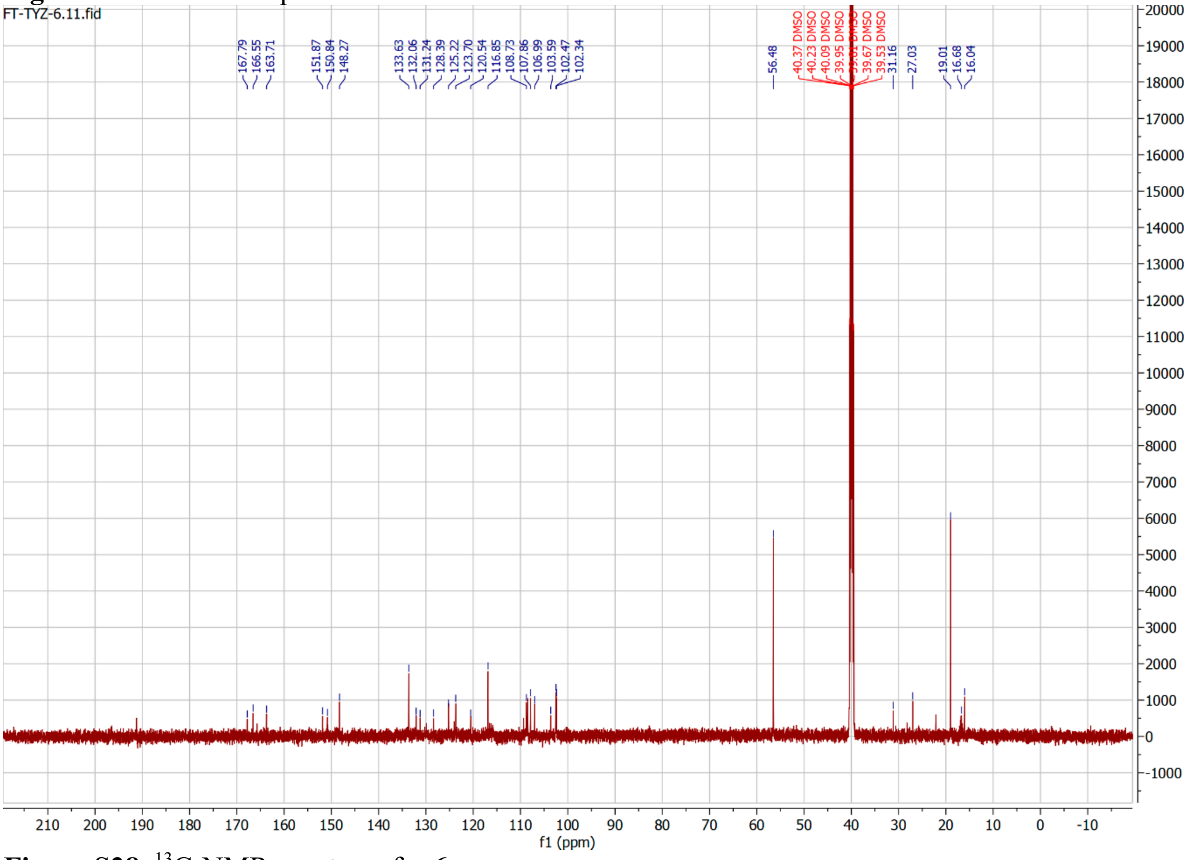


**Figure S28**. $^{13}$C-NMR spectrum for **6**

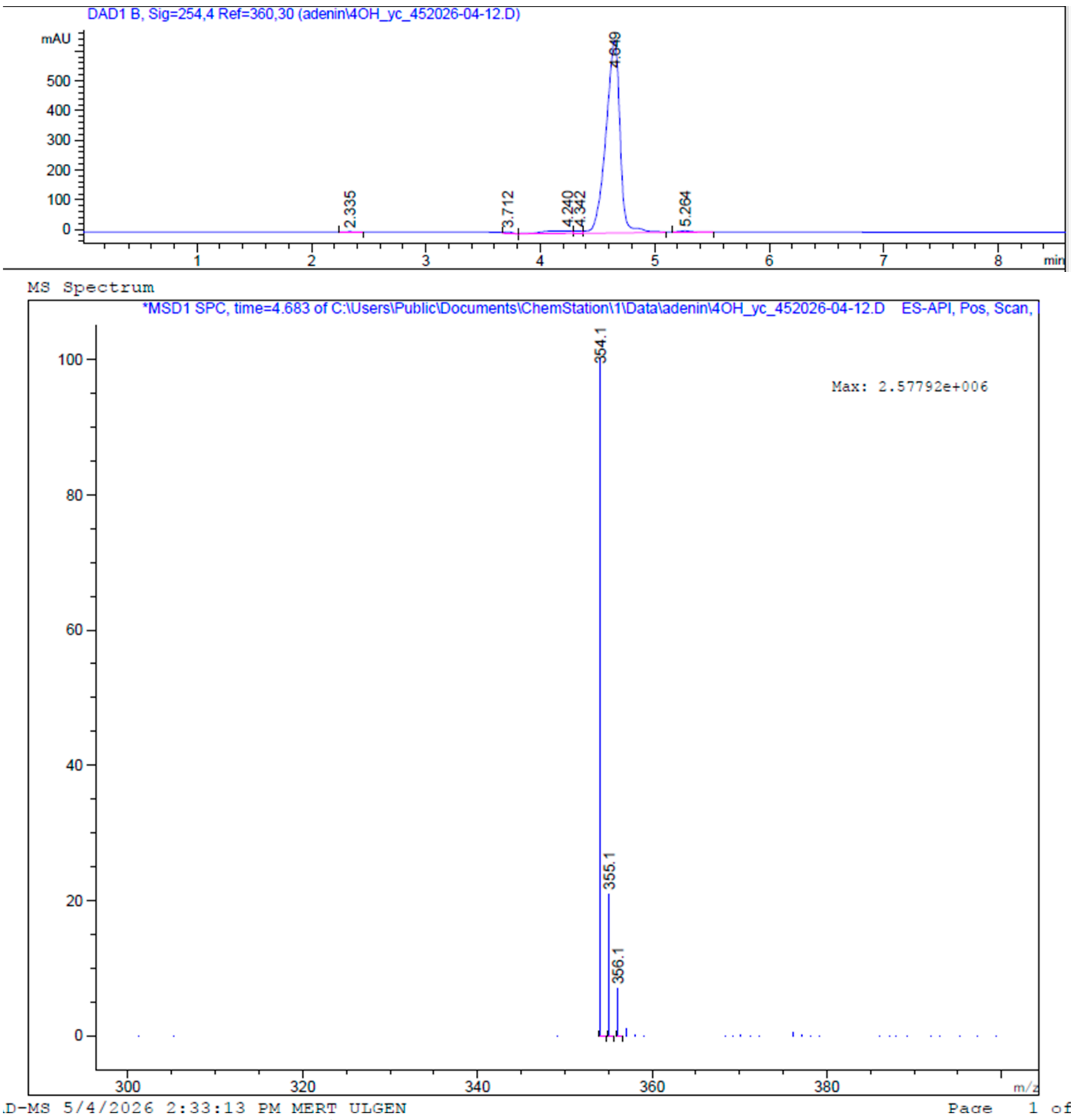


**Figure S29**. LC-MS spectrum for **6**

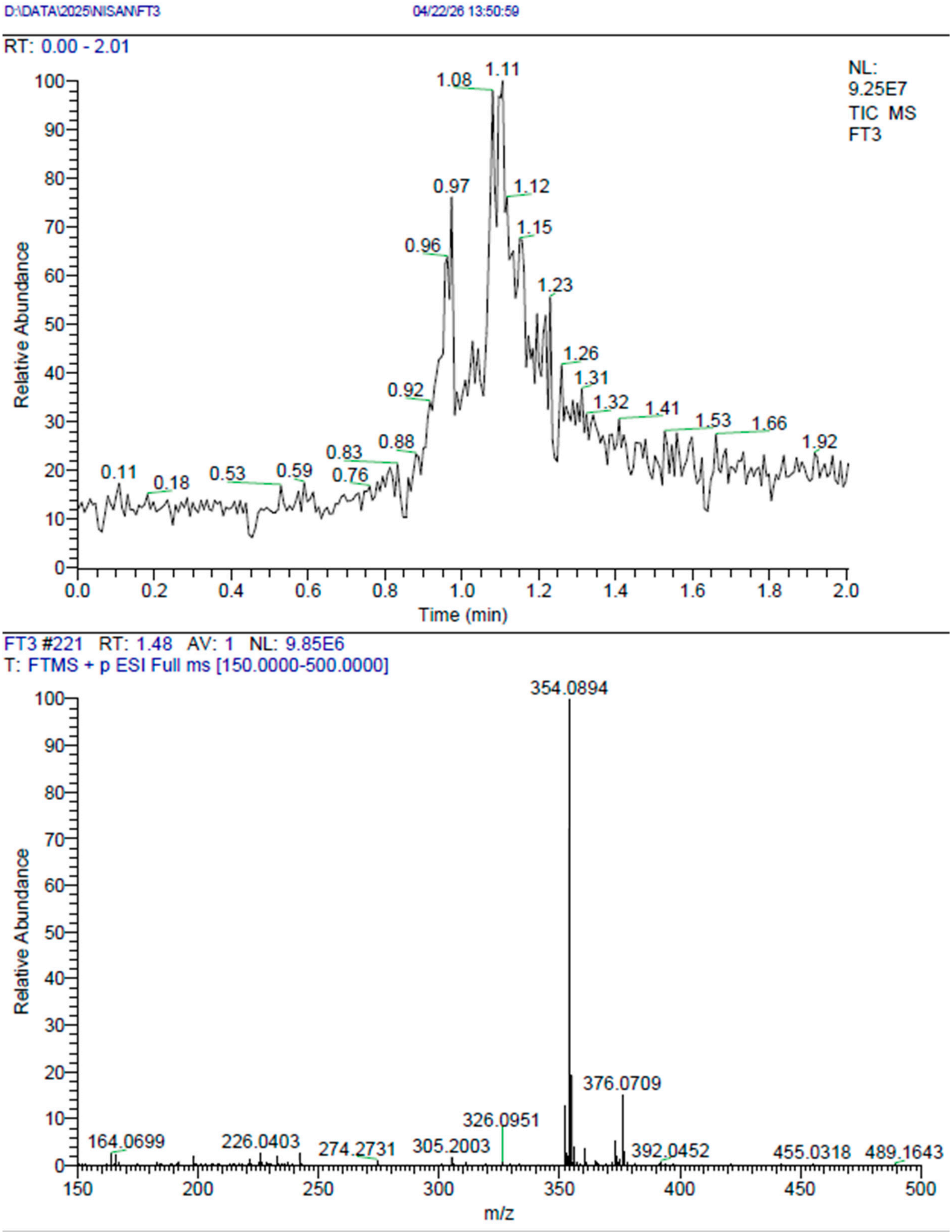


**Figure S30**. LC-HRMS spectrum for **6**

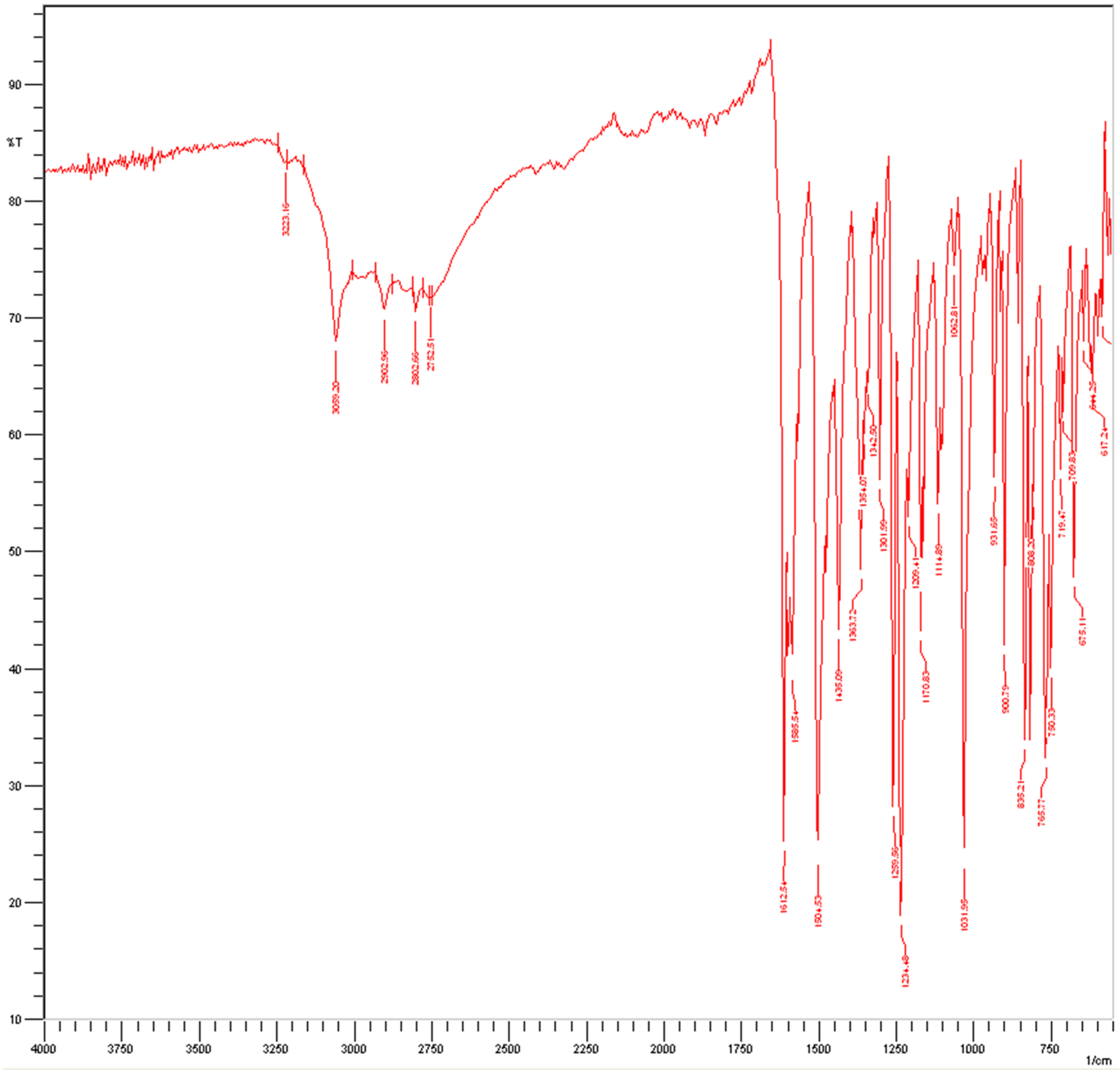

**Figure S31**. IR spectrum for **7**

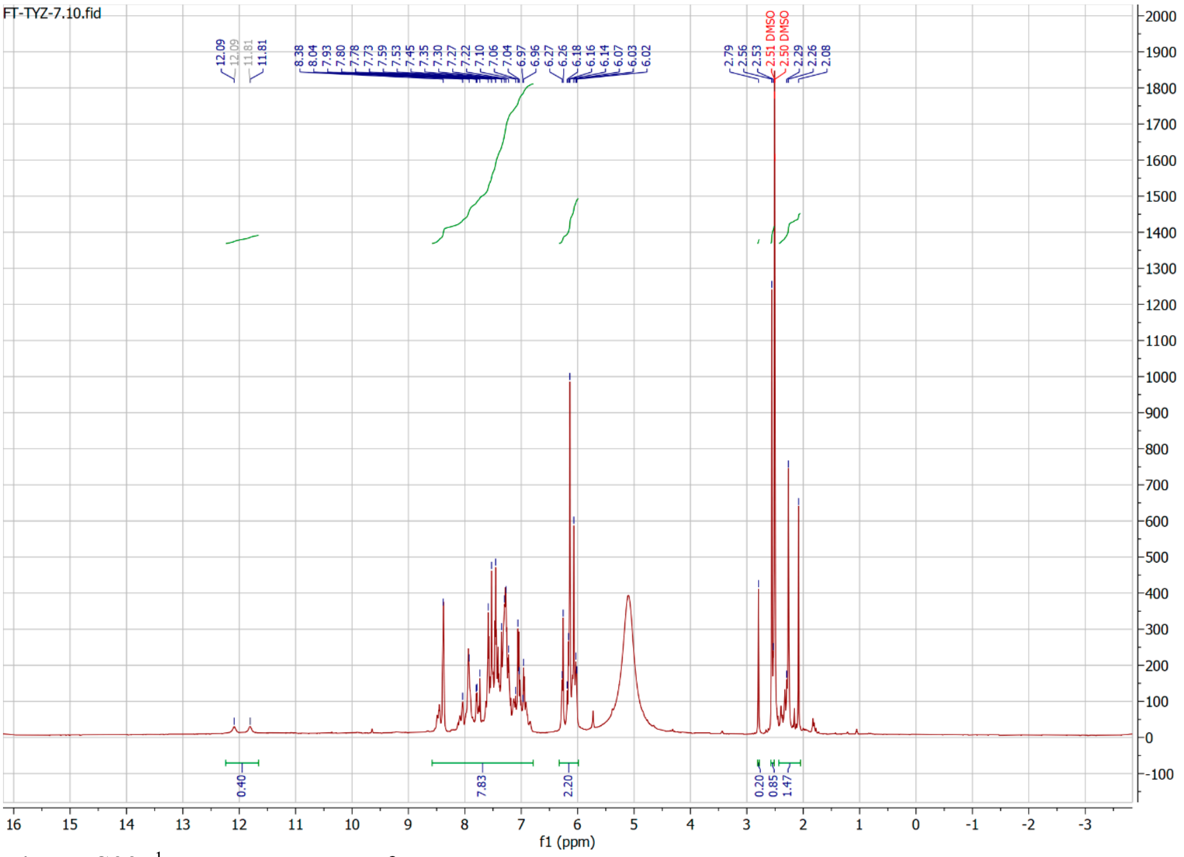

**Figure S32**. $^{1}$H-NMR spectrum for **7**

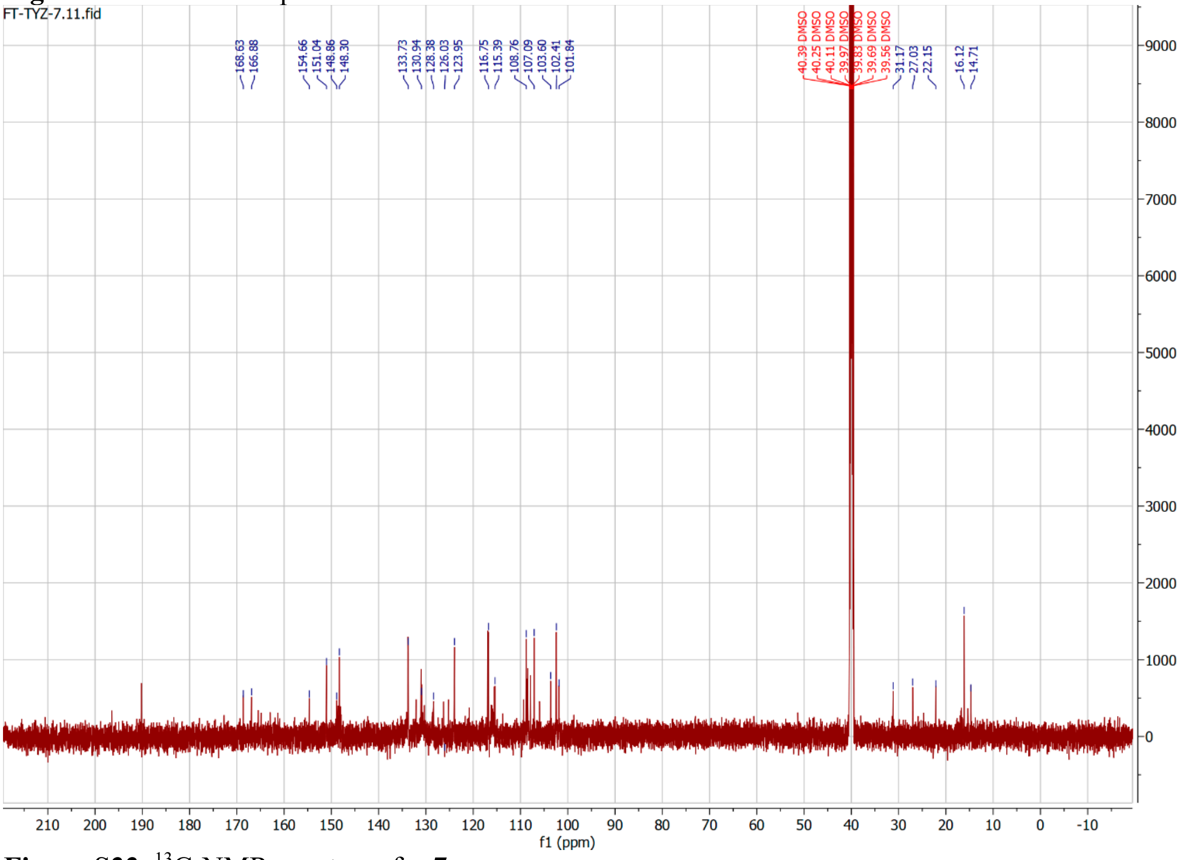

**Figure S33**. $^{13}$C-NMR spectrum for **7**

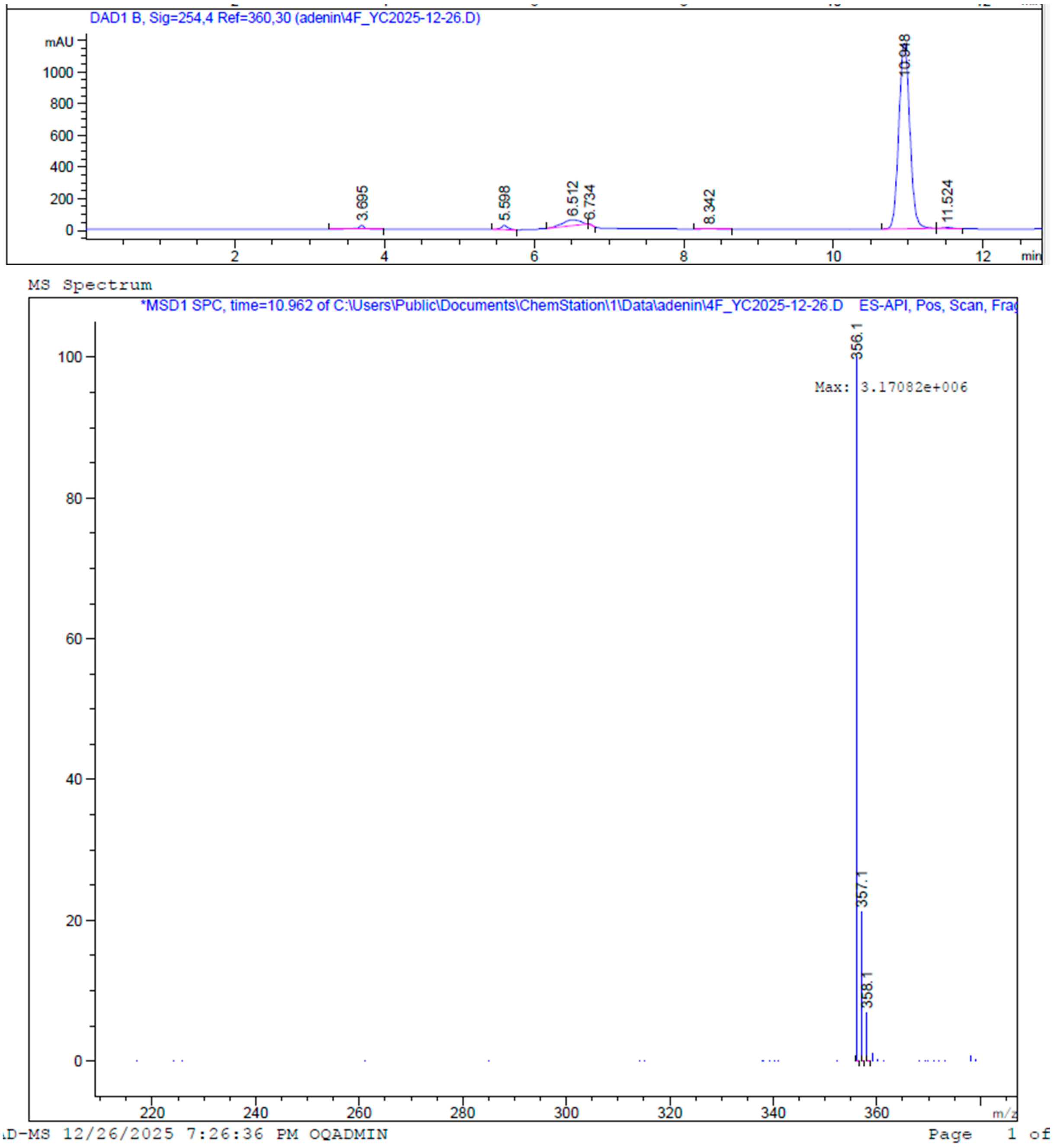


**Figure S34**. LC-MS spectrum for **7**

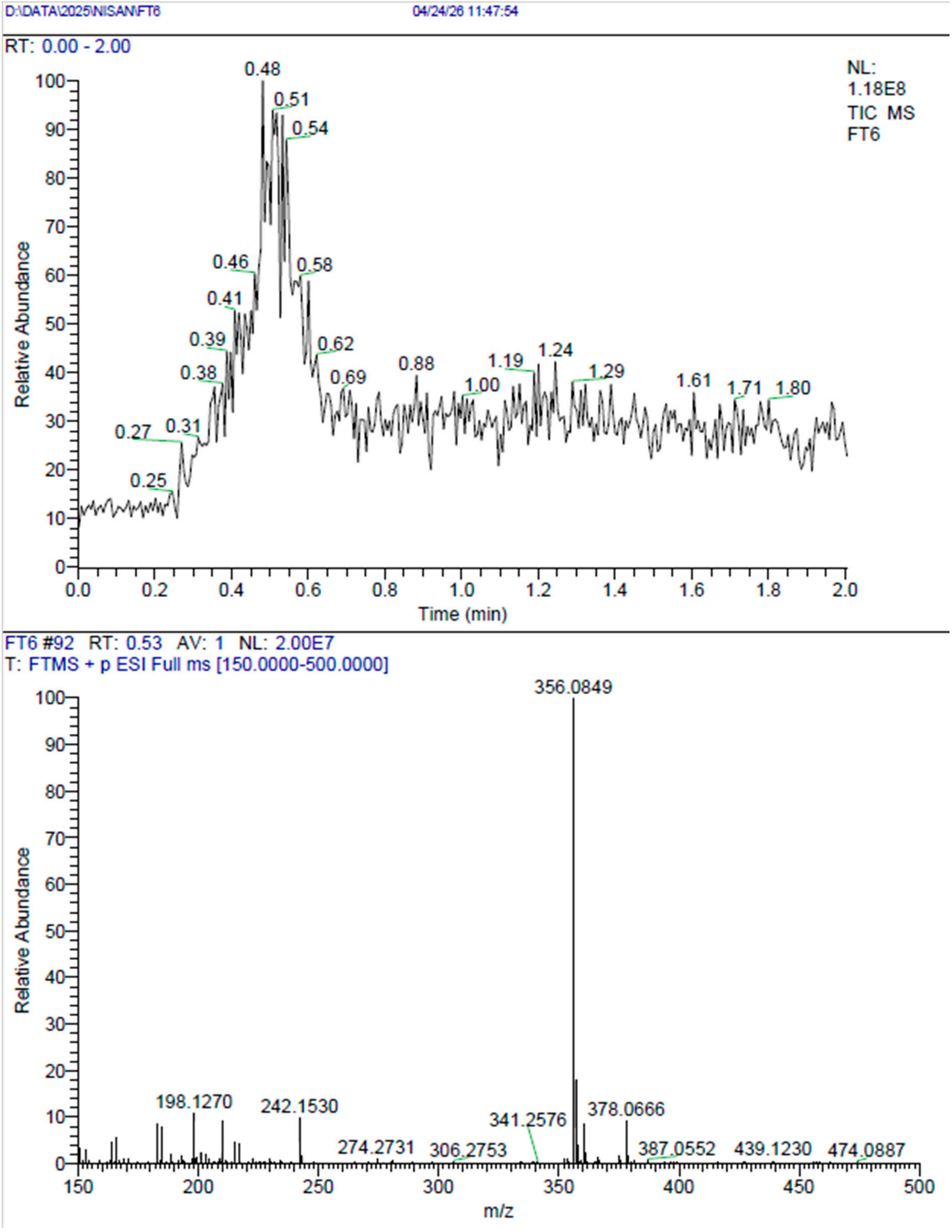


**Figure S35**. LC-HRMS spectrum for **7**

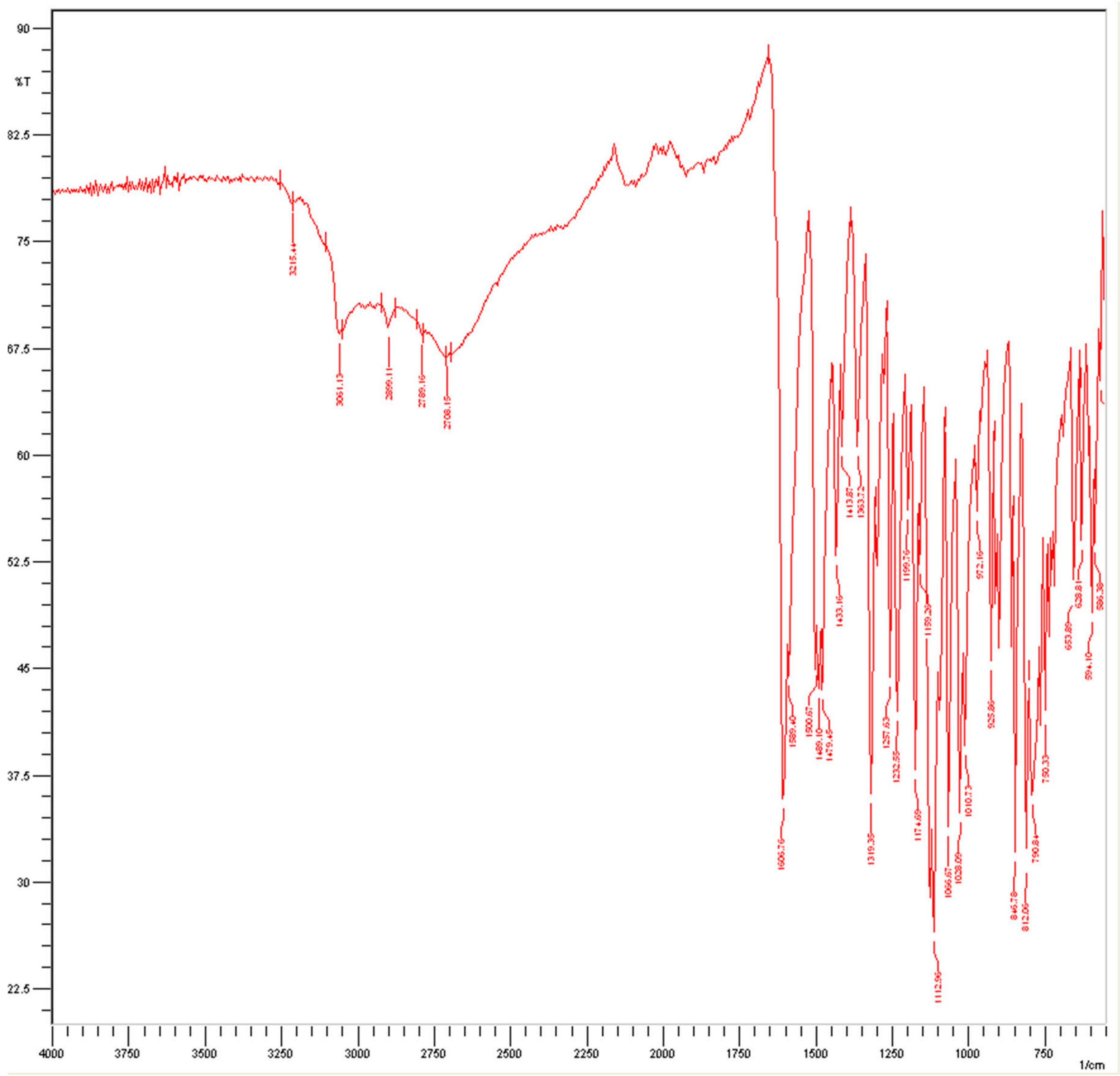


**Figure S36**. IR spectrum for **8**

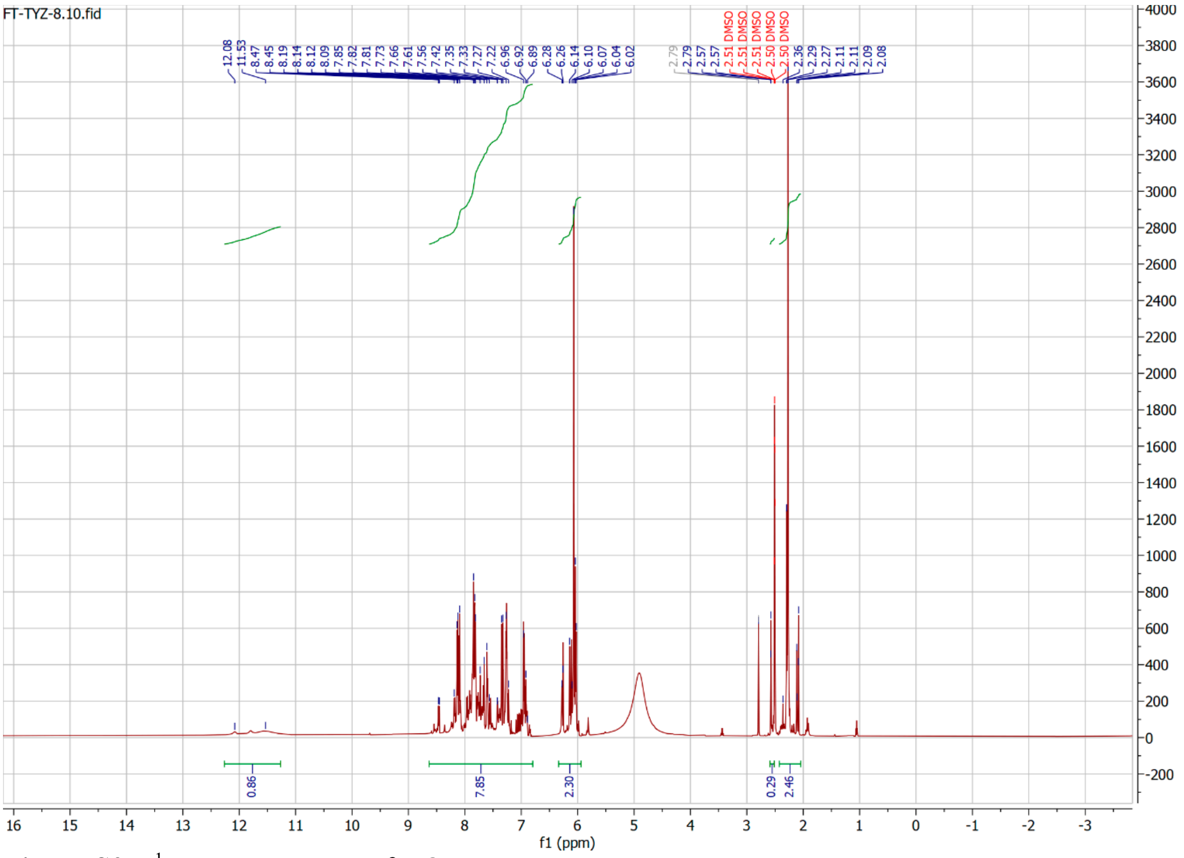


**Figure S37**. $^{1}$H-NMR spectrum for **8**

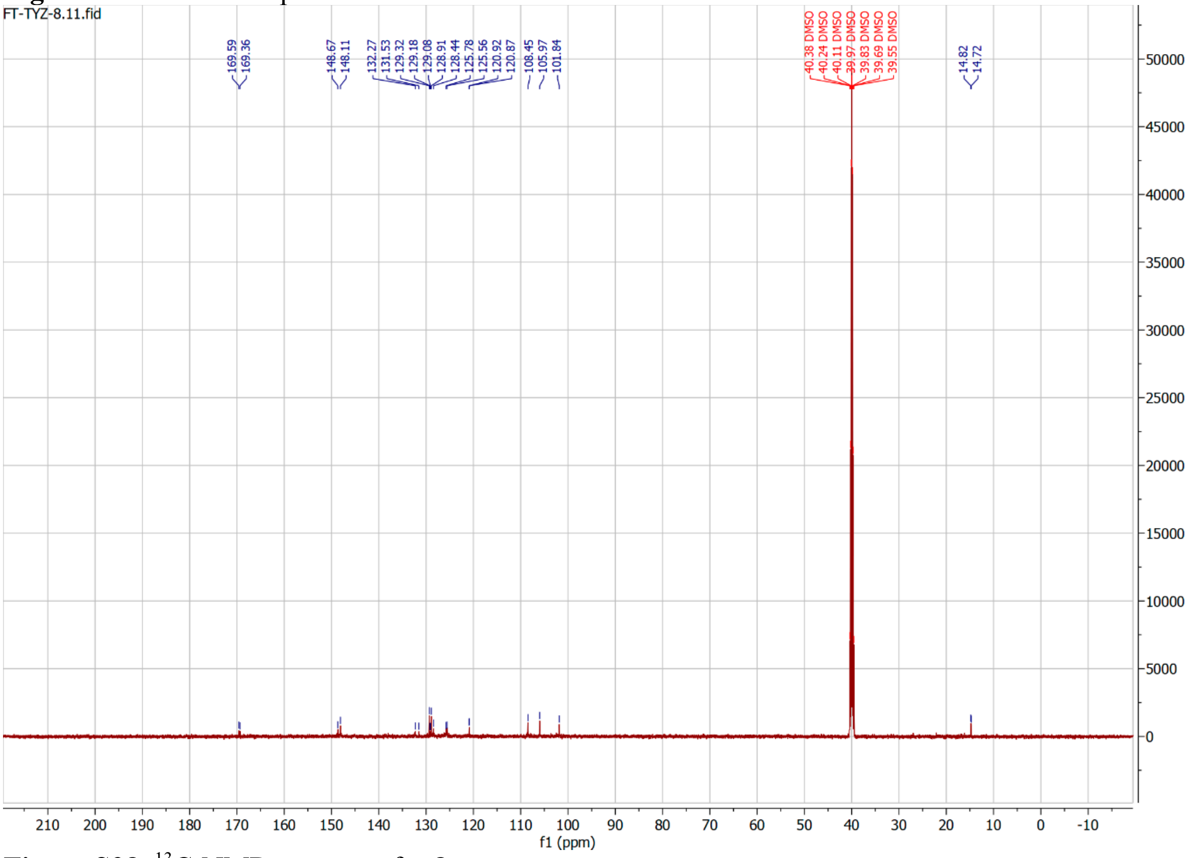


**Figure S38**. $^{13}$C-NMR spectrum for **8**

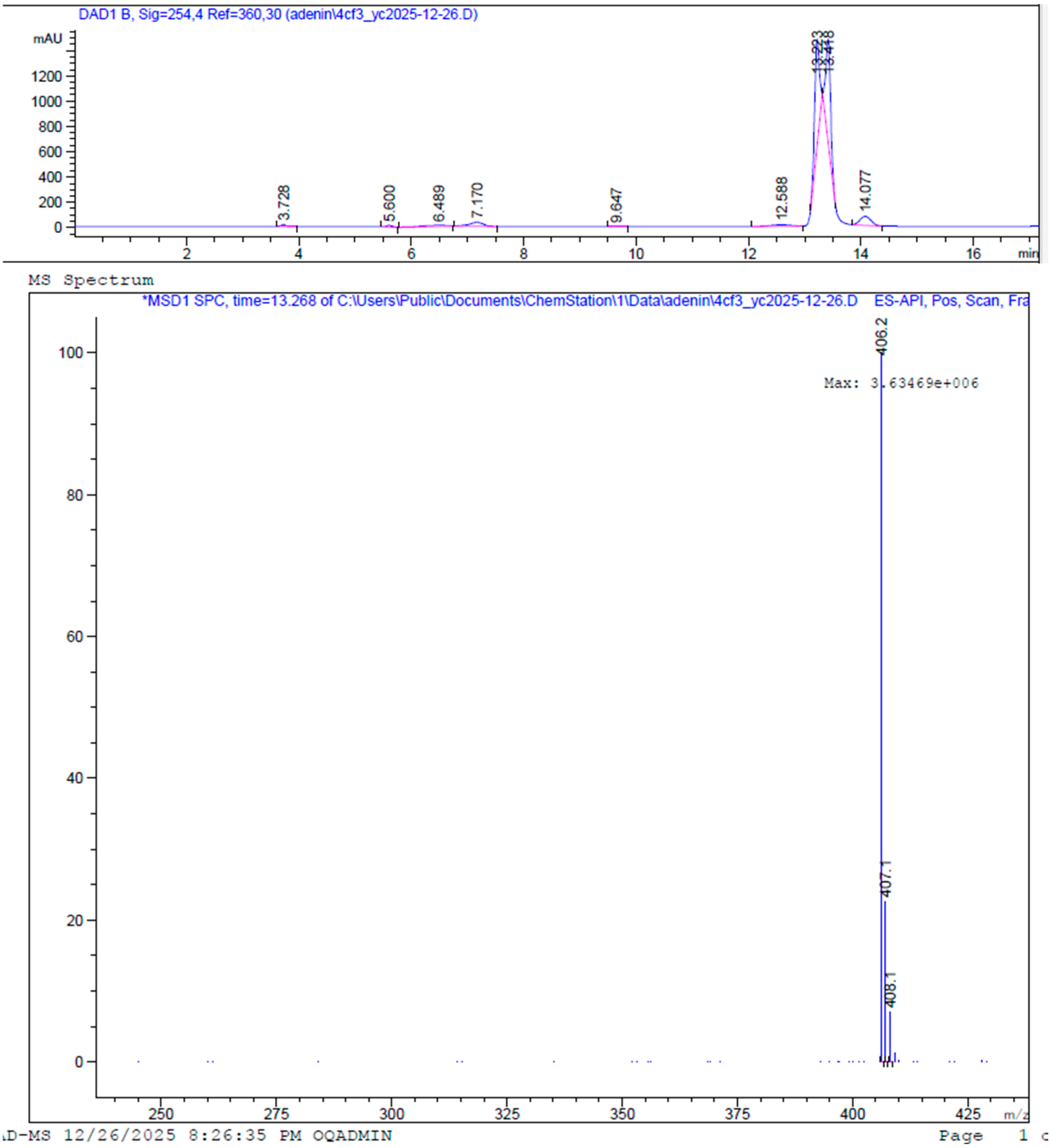

**Figure S39**. LC-MS spectrum for **8**

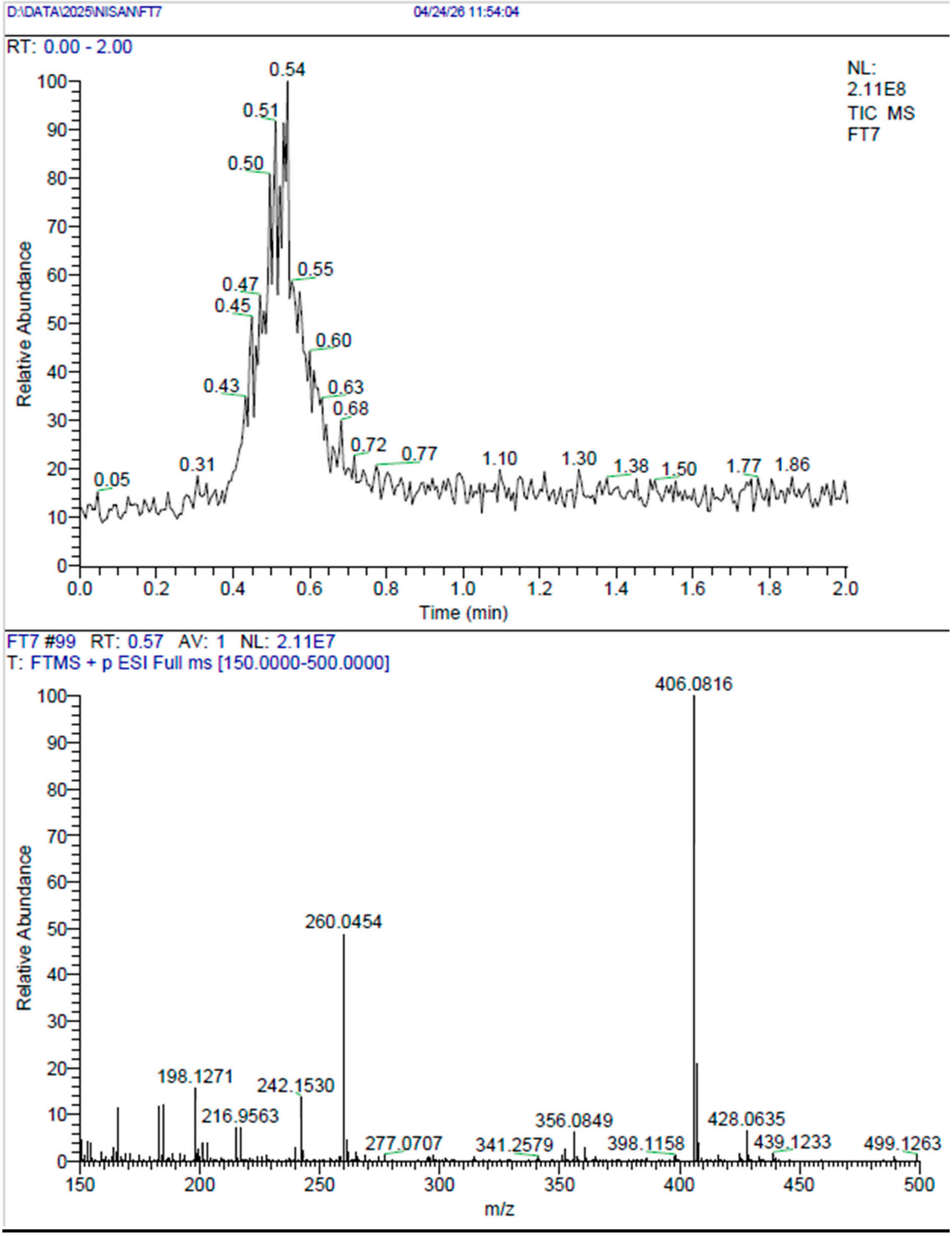


**Figure S40**. LC-HRMS spectrum for **8**

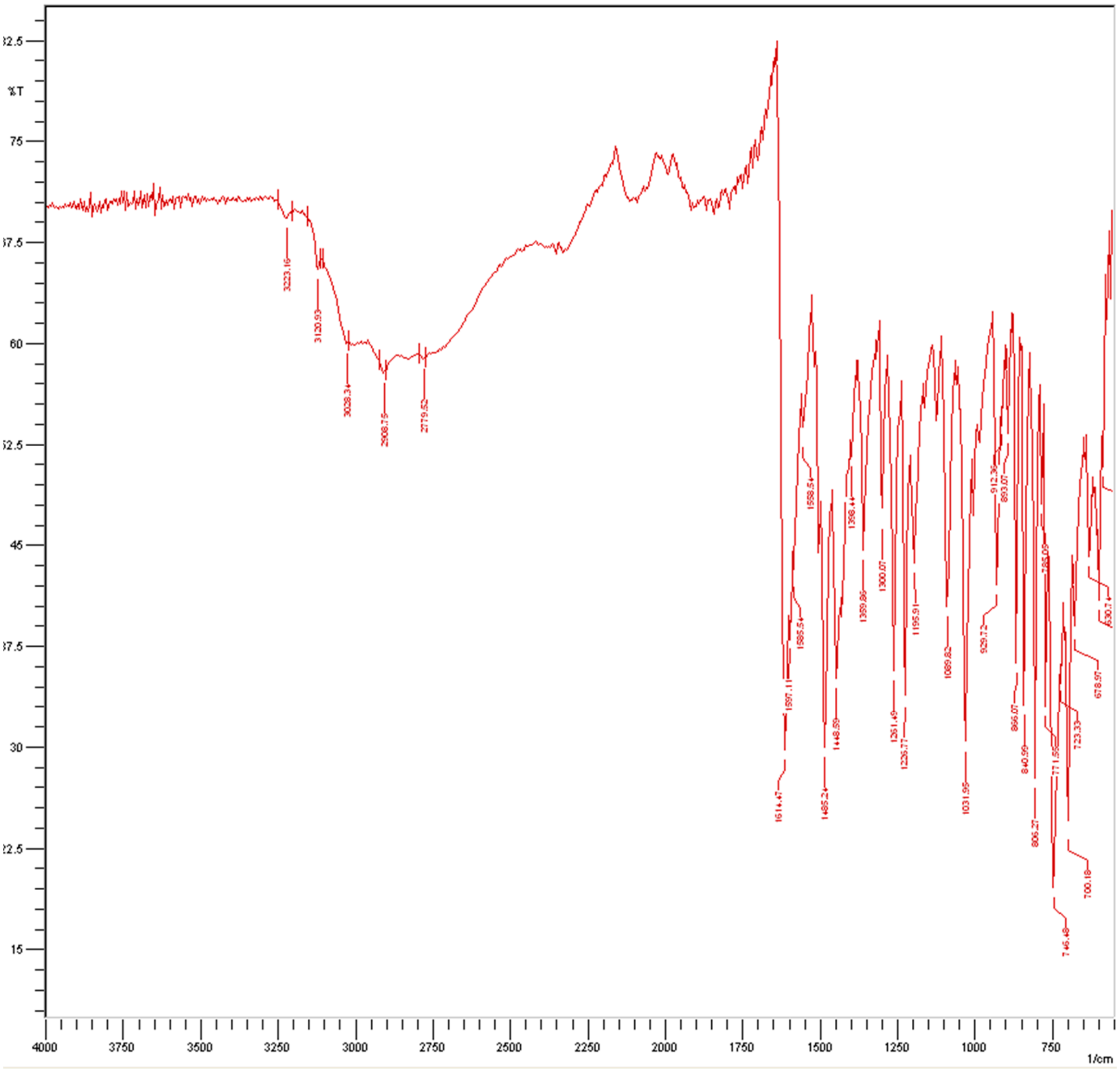


**Figure S41**. IR spectrum for **9**

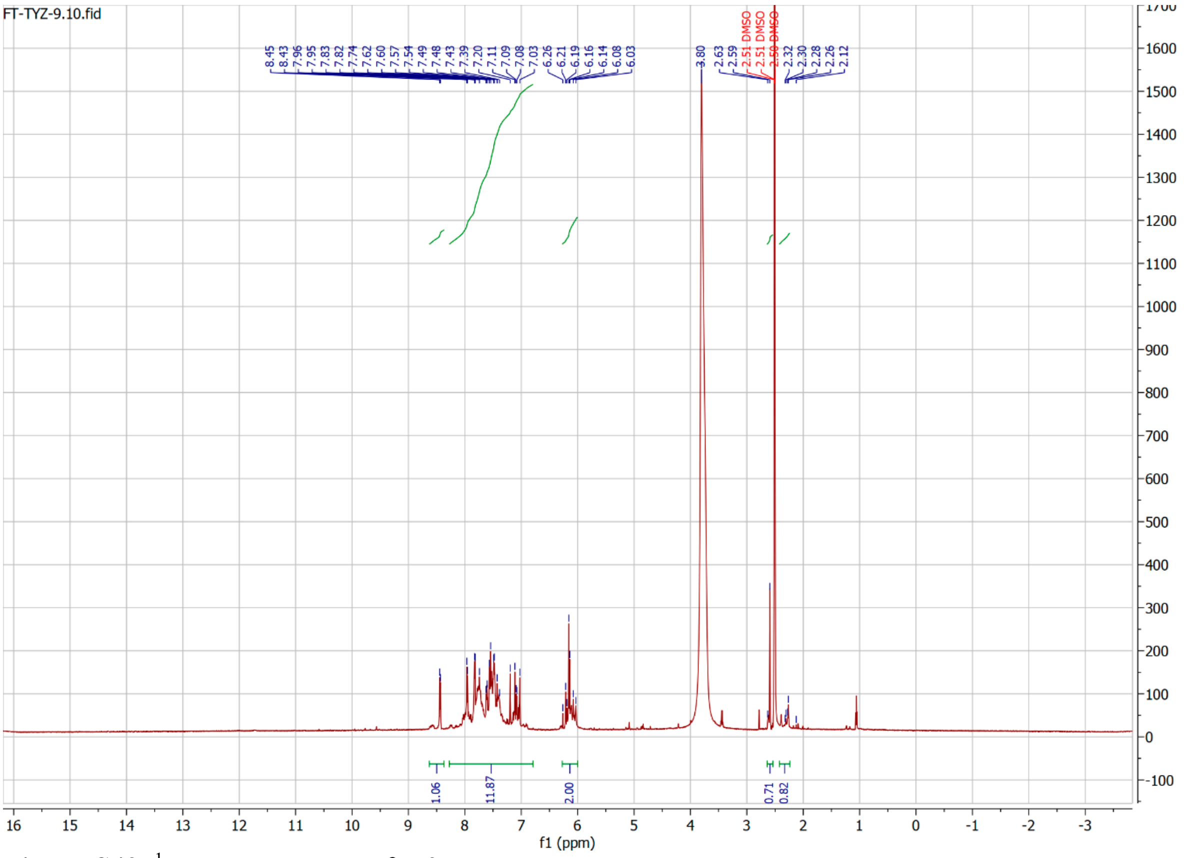

**Figure S42**. $^{1}$H-NMR spectrum for **9**

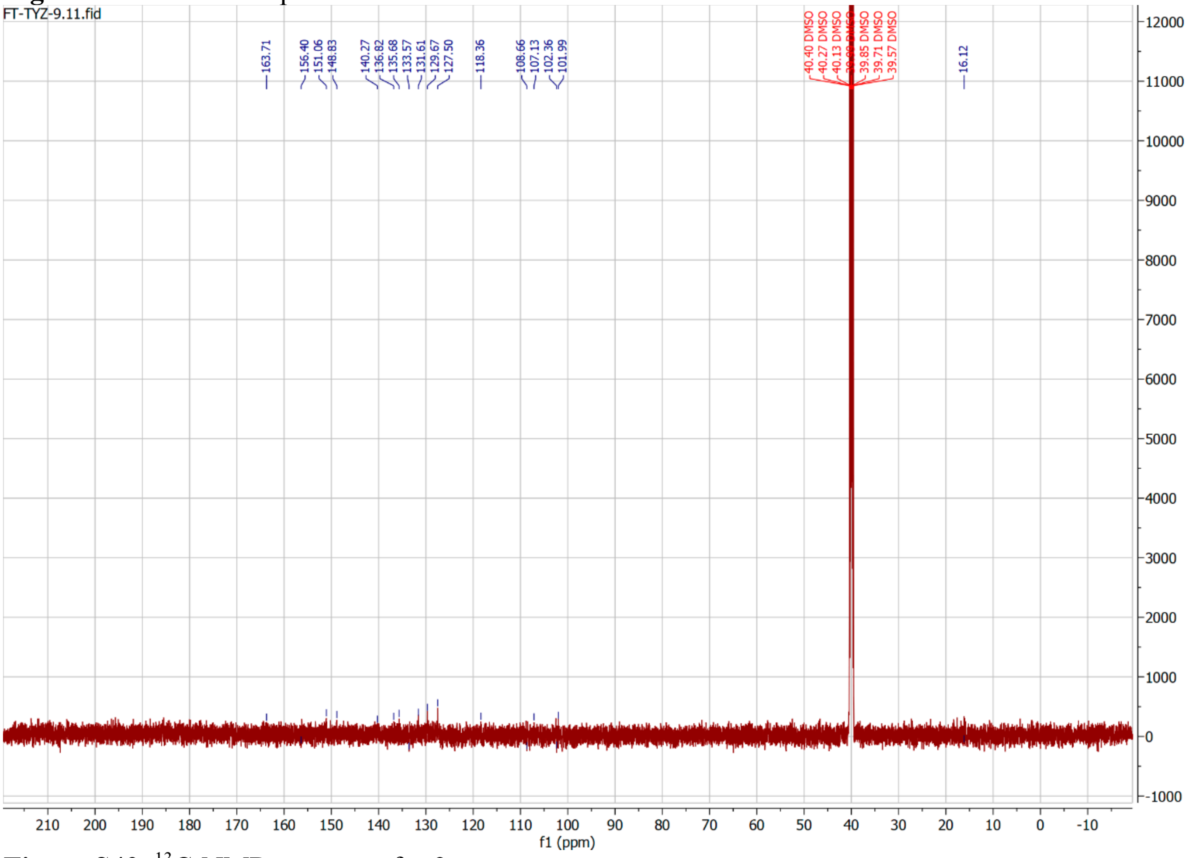

**Figure S43**. $^{13}$C-NMR spectrum for **9**

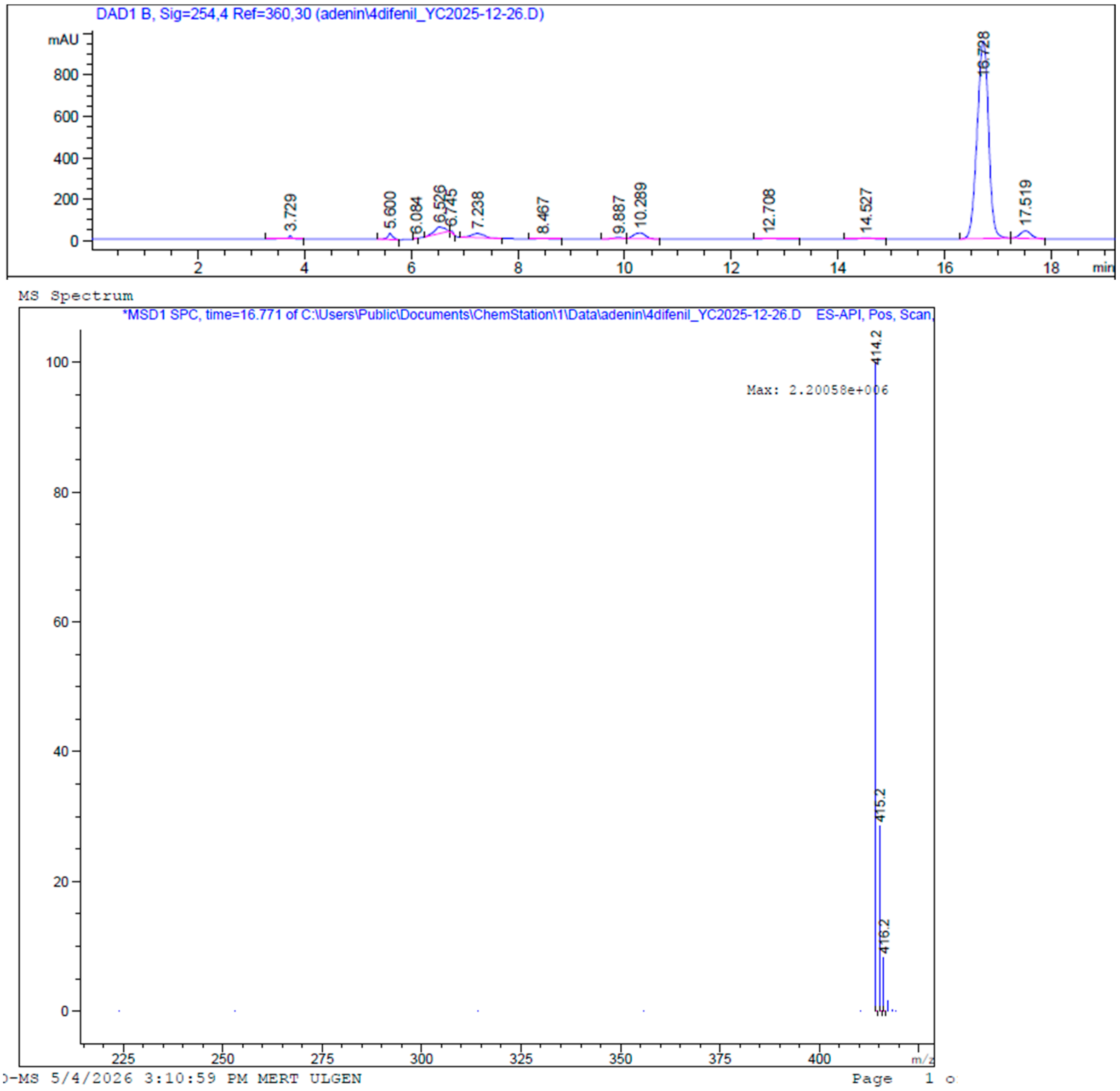

**Figure S44**. LC-MS spectrum for **9**

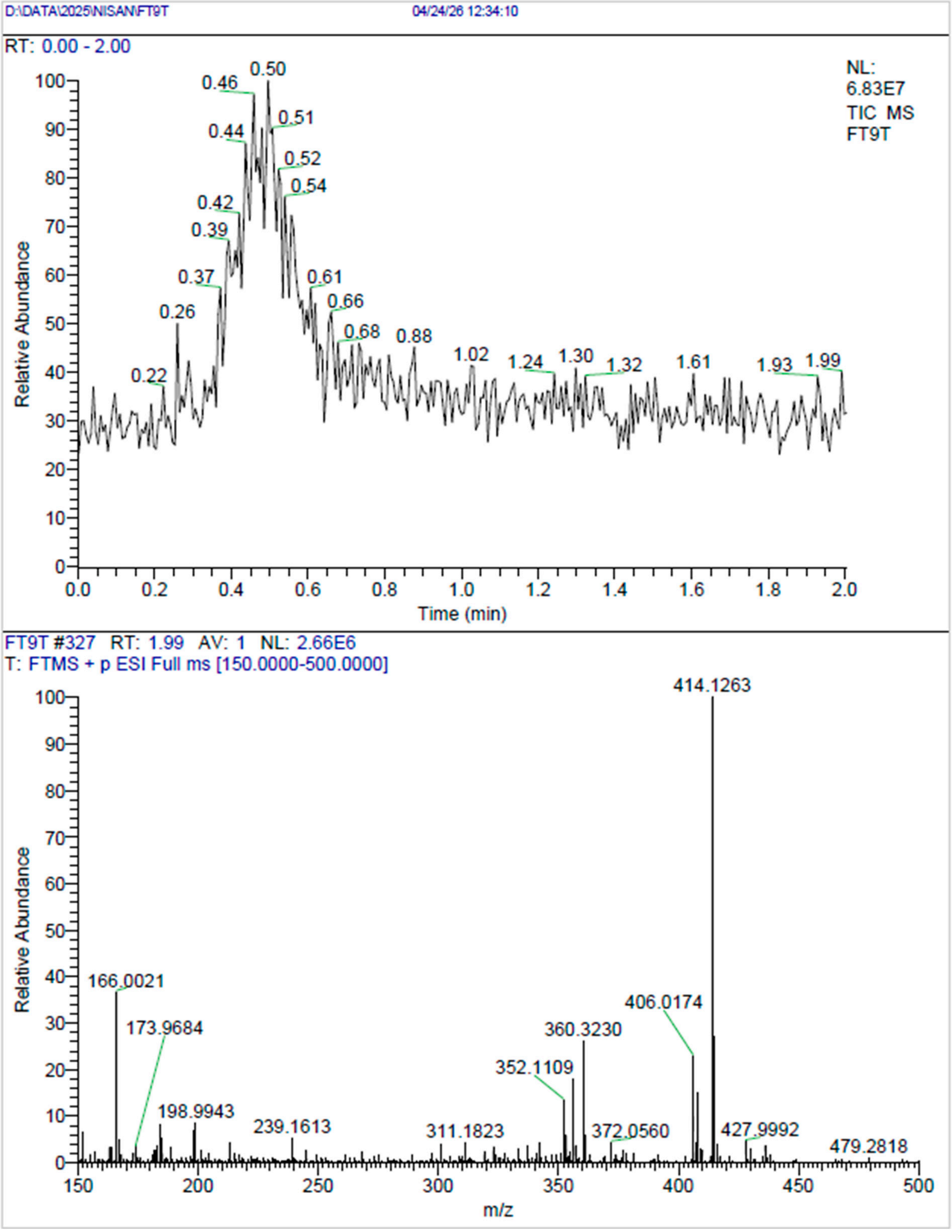


**Figure S45**. LC-HRMS spectrum for **9**

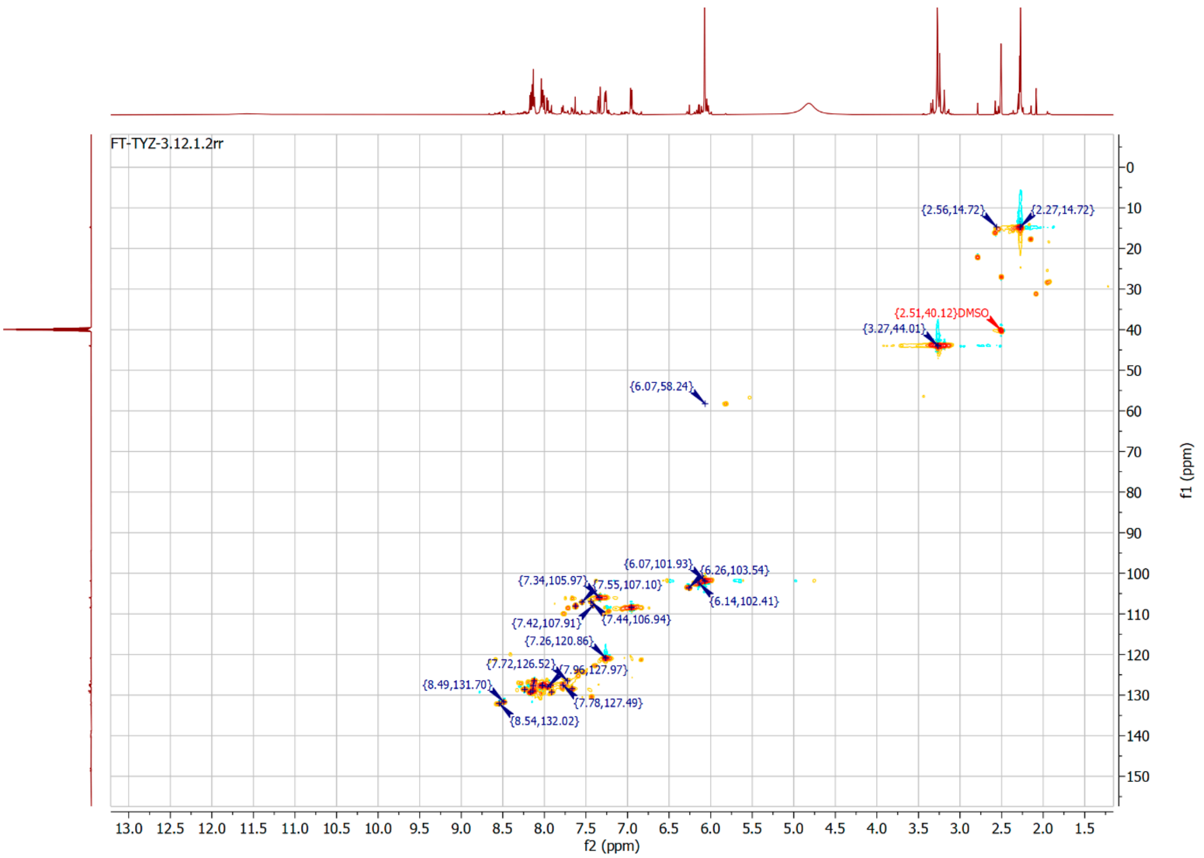


**Figure S46**. HSQC spectrum for **3**

| **Name of complex** | **2d** | **3d** |
|---|---|---|
| 4M0E-3 | 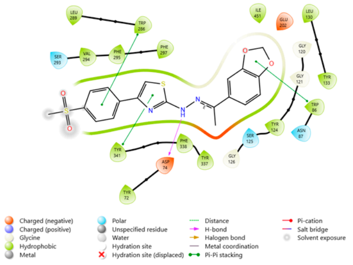 | 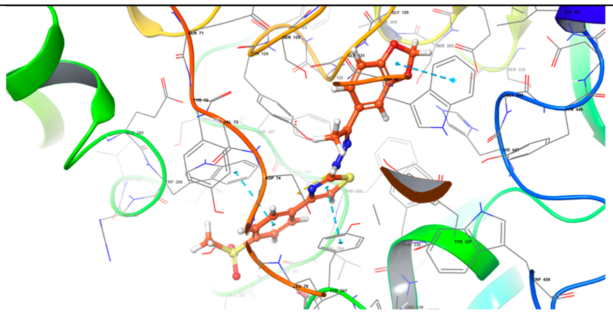 |
| 4M0E-5 | 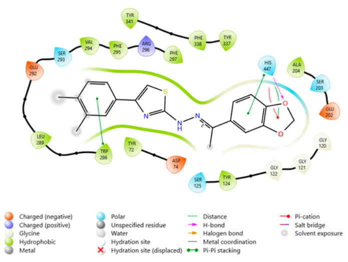 | 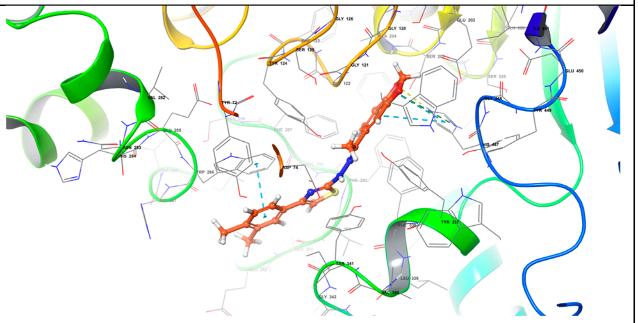 |

| | | |
|---|---|---|
| 4M0E-Galantamin | 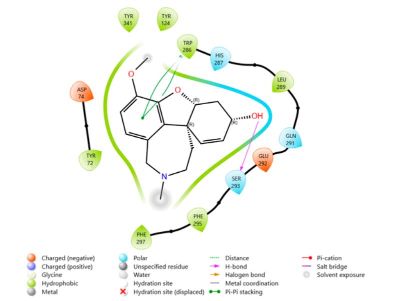 | 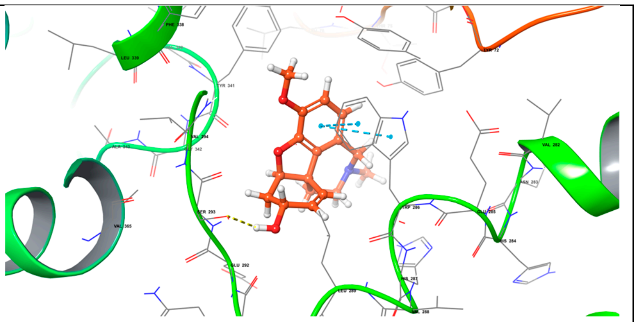 |
| 4M0E-1yl | 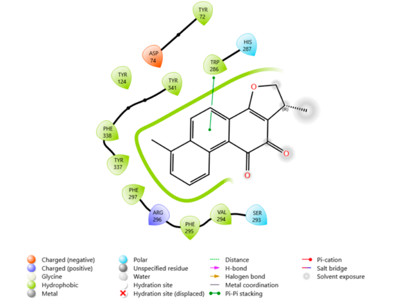 | 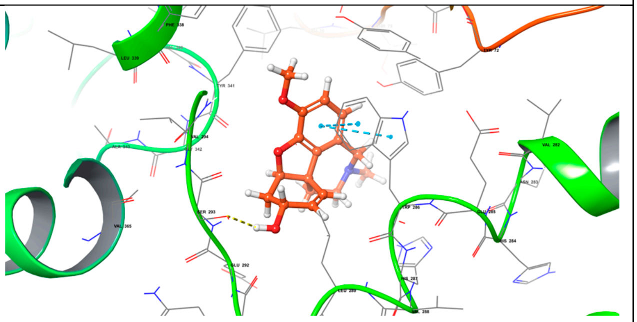 |
| 6QAA-3 | 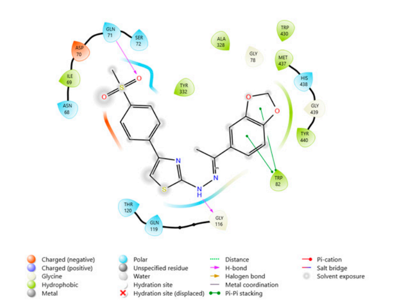 | 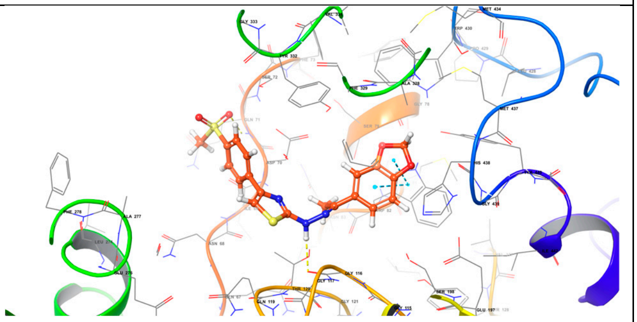 |
| 6QAA-5 | 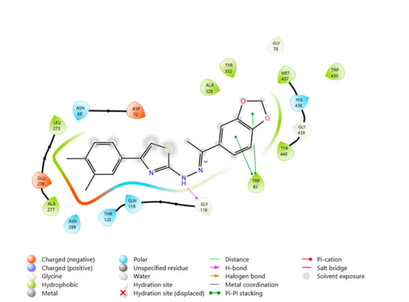 | 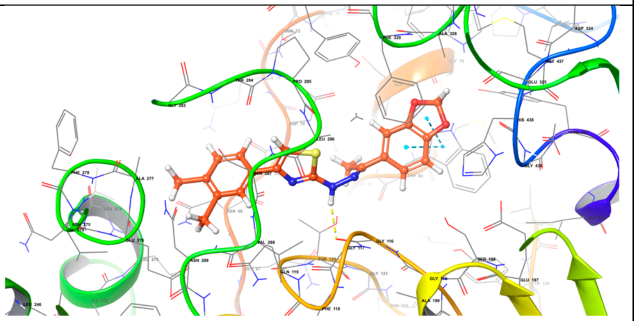 |
| 6QAA-Galantamin | 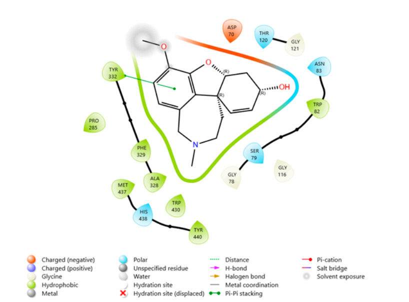 | 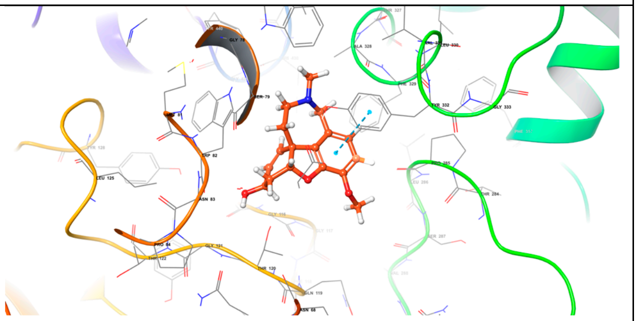 |

| | | |
|---|---|---|
| 6QAA-hun | 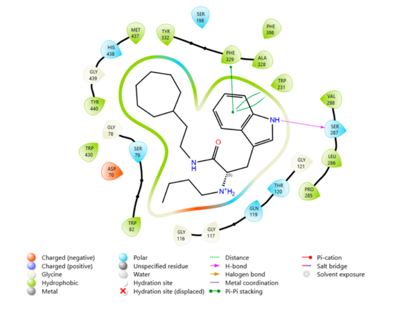 | 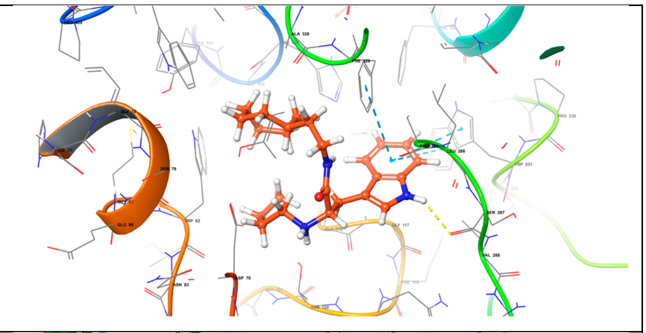 |
| 5NN8-3 | 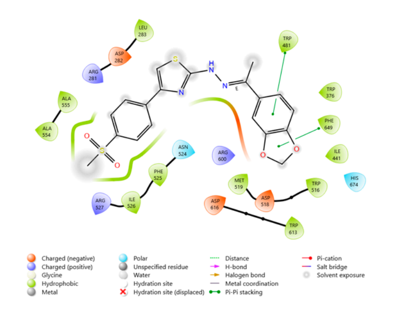 | 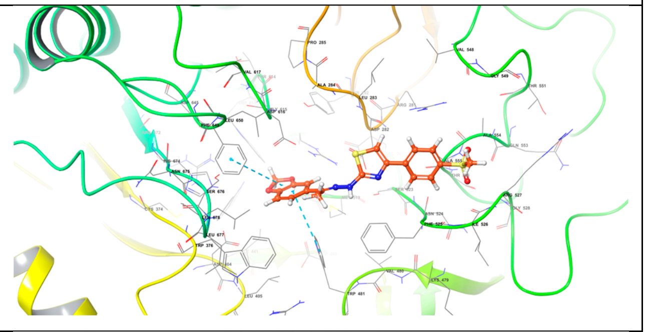 |
| 5NN8-5 | 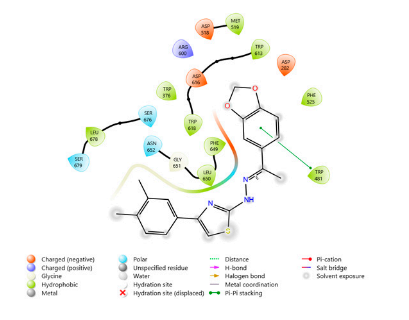 | 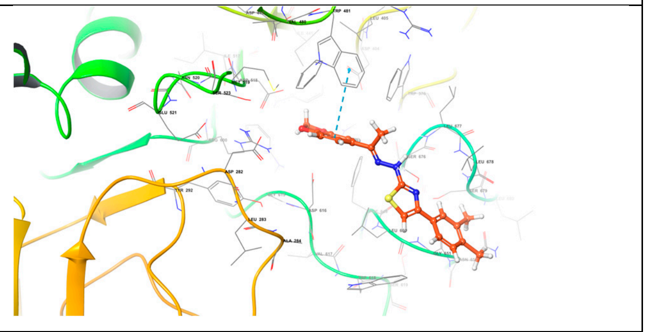 |
| 5NN8-Acarbose | 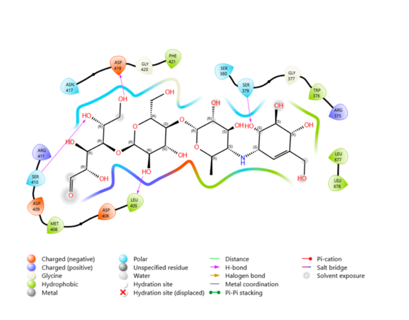 | 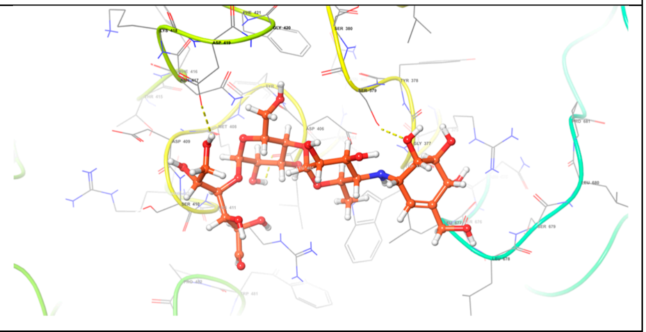 |
| 4GQR-3 | 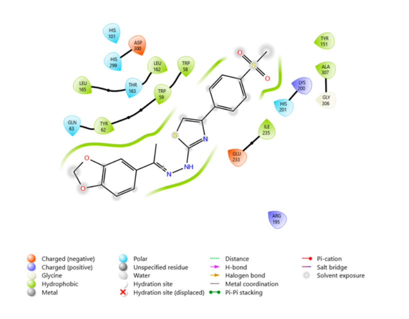 | 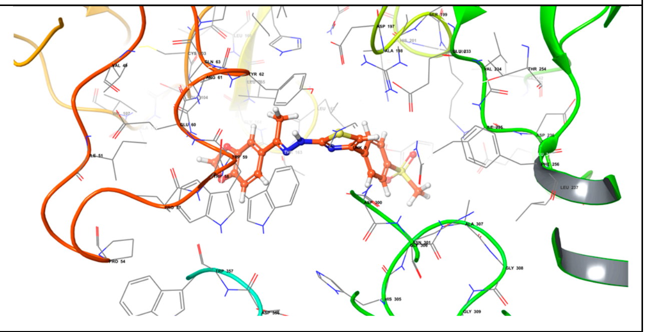 |

| | | |
|---|---|---|
| 4GQR-5 | 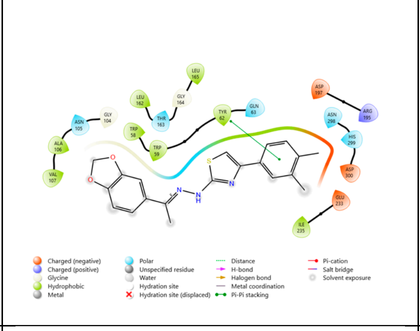 | 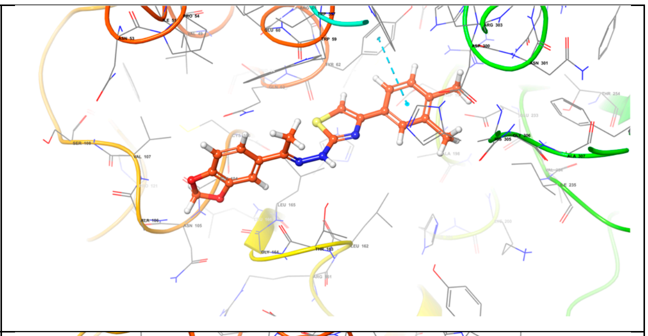 |
| 4GQR-Acarbose | 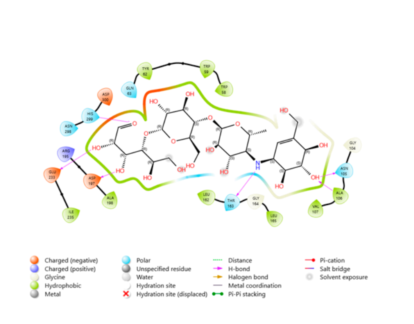 | 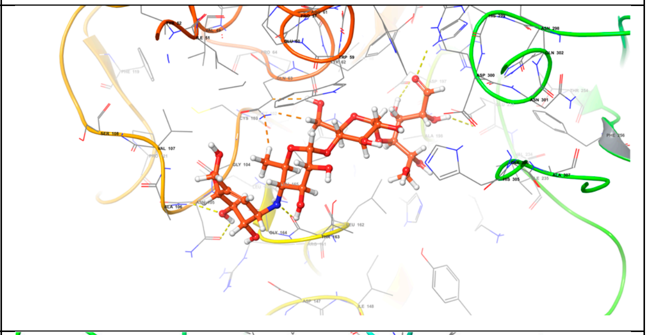 |
| 4GQR-myc | 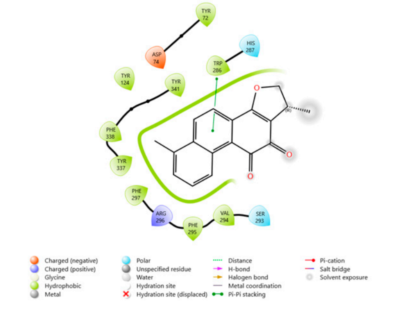 | 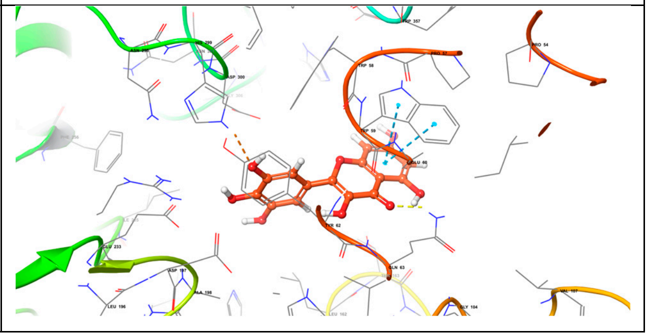 |

**Figure S47**. 2D and 3D interactions between ligands and protein 5D41.

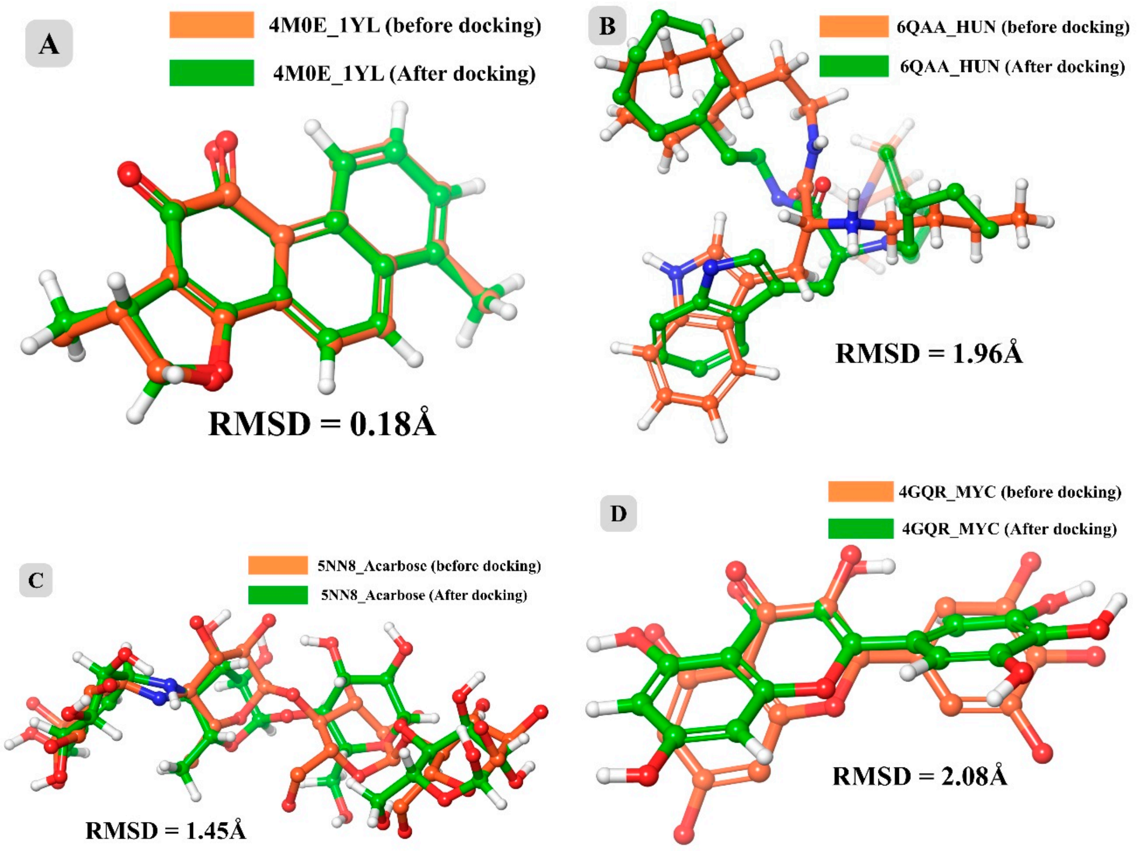


**Figure S48**. Validation of docking protocol through redocking of co-crystal ligand in binding pocket of respective targets. Superimposition of experimental co-crystal ligand pose (Orange) before docking and re-docked pose (Green) after docking **(A)** 4M0E–1YL (acetylcholinesterase), RMSD = 0.18 Å; **(B)** 6QAA–HUN (butyrylcholinesterase), RMSD = 1.96 Å; **(C)** 5NN8–Acarbose (lysosomal acid α-glucosidase), RMSD = 1.45 Å; **(D)** 4GQR–MYC (pancreatic α-amylase), RMSD = 2.08 Å.
